# The Headphone and Loudspeaker Test–Part II: A comprehensive method for playback device screening in Internet experiments

**DOI:** 10.3758/s13428-022-02048-3

**Published:** 2023-01-17

**Authors:** Yves Wycisk, Kilian Sander, Benedetto Manca, Reinhard Kopiez, Friedrich Platz

**Affiliations:** 1 Hanover Music Lab, Hanover University of Music, Drama and Media, Neues Haus 1, 30175 Hannover, Germany; 2 Department of Mathematics and Computer Science, University of Cagliari, Cagliari, Italy; 3 State University of Music and Performing Arts Stuttgart, Stuttgart, Germany

**Keywords:** Controlling confounding variables, Combining screening tests, Utility, Headphone prevalence, Predictive values, Split convince compare

## Abstract

HALT (The Headphone and Loudspeaker Test) Part II is a continuation of HALT Part I. The main goals of this study (HALT Part II) were (a) to develop screening tests and strategies to discriminate headphones from loudspeakers, (b) to come up with a methodological approach to combine more than two screening tests, and (c) to estimate data quality and required sample sizes for the application of screening tests. Screening Tests A and B were developed based on psychoacoustic effects. In a first laboratory study (*N* = 40), the two tests were evaluated with four different playback devices (circumaural and intra-aural headphones; external and laptop loudspeakers). In a final step, the two screening tests A and B and a previously established test C were validated in an Internet-based study (*N* = 211). Test B showed the best single-test performance (sensitivity = 80.0%, specificity = 83.2%, AUC = .844). Following an epidemiological approach, the headphone prevalence (17.67%) was determined to calculate positive and negative predictive values. For a user-oriented, parameter-based selection of suitable screening tests and the simple application of screening strategies, an online tool was programmed. HALT Part II is assumed to be a reliable procedure for planning and executing screenings to detect headphone and loudspeaker playback. Our methodological approach can be used as a generic technique for optimizing the application of any screening tests in psychological research. HALT Part I and II complement each other to form a comprehensive overall concept to control for playback conditions in Internet experiments.

**Supplementary Information:**

The online version contains supplementary material available at 10.3758/s13428-022-02048-3.

## Introduction

### Scope of the study

A high degree of control over the playback situation is important in conducting experiments on auditory perception. Maximum control is primarily possible in laboratory experiments. However, if a large sample size is needed, an Internet experiment is usually the method of choice. In this situation, having a high number of participants and, at the same time, maintaining a high level of control seems mutually exclusive. With the Headphone and Loudspeaker Test (HALT) Part I and Part II, we wanted to provide a tool to counter this predicament by remote testing playback device characteristics in Internet experiments. In the previous study, HALT Part I (Wycisk et al., 2022), we suggested a procedure to standardize level adjustments, detect stereo/mono playback and assess lower frequency limits of the playback devices. Subsequently, in HALT Part II, we focused on the identification of playback device types. A comprehensive concept to distinguish between headphones and loudspeakers will be suggested. HALT Part I and Part II together form the complete HALT procedure that can help improve the quality of Internet experiments on auditory perception.

In general, there can be various reasons to control for the type of sound reproduction device. First, playback device types, such as headphones or loudspeakers, can have an impact on how participants perceive stimuli. For example, Zelechowska et al. (2020) investigated the effects of headphones and loudspeaker playback on spontaneous body movement to rhythmic music. The authors found a “significant higher mean velocity of the head and body motion” (p. 14) in the headphone condition compared to the loudspeaker condition. For this reason, headphones or loudspeaker playback can be regarded to as confounding factors that should be controlled for. Second, a control procedure may be necessary due to the use of special audio samples, such as 3D binaural headphone mixes. As the 3D impression of such stimuli would be lost in case of loudspeaker reproduction, it must be ensured that the participants use headphones.

Existing playback device screening tests are a promising possibility to control for either loudspeaker or headphone playback. However, it is a major challenge to assess, compare, and select tests for a specific application. This study aimed to overcome these challenges. To compare the quality and capability of screening tests in general, several parameters must be determined. Those parameters help in selecting a screening test and screening strategy for a specific use case. In the current study, we used signal detection theory (SDT; Macmillan & Creelman, 2005; Treat & Viken, 2012) as a paradigm for evaluating screening tests. The detection of headphone or loudspeaker playback is logically similar to the detection of a disease. In both cases a screening test can be used to check whether a characteristic is present or absent. Mathematical and statistical methods and standards from disease detection can be transferred. For that reason, we expanded the analysis by using an epidemiological approach. In the following, we introduce the most important terms and parameters in this context.

### Nomenclature, definitions, and fundamentals of diagnostics

We define a screening test as a procedure with its tasks and stimuli for which a certain sensitivity and specificity can be reported. In contrast, a screening strategy encompasses the initiation, embedding, and targeted application of a certain screening test. A screening test containing more than one task or item requires a threshold or cutoff for classification that is the minimum number of correct responses from which the test result is positive.

“Sensitivity is defined as the ability of a test to detect all those with the disease in the screened population” (Miller, 2014, p. 767). In our application, the presence of headphones is equated with the presence of the disease whereas the absence of headphones is equated with being free of the disease. We decided on this to ensure comparability with parameters from other studies. Theoretically, the assignment can also be inverted. In terms of SDT terminology, headphones are the signal to be detected. A person to whom the tests give a positive result is classified as a headphone user. A person to whom the tests give a negative result is classified as a loudspeaker user.

In our context, a headphone user for whom the screening test yields a positive result is considered a *true positive* (*TP*) case or a *hit* whereas one with a negative test result is considered a *false negative* (*FN*) case or a *miss*. Following from this, the sensitivity or *hit rate* according to SDT expresses the proportion of true headphone users for whom the screening test gave a positive result (*TP*). Formulated as a conditional probability, sensitivity is the probability of a positive test result given the presence of headphones. See Eq. (1) (Miller, 2014, p. 768) for the calculation (*P* for probability).

1 $$\textrm{Sensitivity}= Sen=P\left(\textrm{test}\ \textrm{positive}|\textrm{headphones}\right)=\frac{TP}{TP+ FN}$$

“Specificity is defined as the ability of a test to detect all those free of the disease in the screened population” (Miller, 2014, p. 767). In our application, a loudspeaker user for whom the test gives a negative result is considered a *true negative* (*TN*) case or a *correct rejection* whereas one with a positive result is considered a *false positive* (*FP*) case or a *false alarm*. The specificity or, in terms of SDT, correct rejection rate expresses the proportion of true loudspeaker users for whom the screening test gave a negative result (*TN*). See Table 1 for the confusion matrix regarding true condition and screening test result. Formulated as a conditional probability, specificity is the probability of a negative test result given the absence of headphones, that is, the presence of loudspeakers. See Eq. (2) (Miller, 2014, p. 768) for the calculation.

2 $$\textrm{Specificity}= Spe=P\left(\textrm{test}\ \textrm{negative}|\textrm{loudspeakers}\right)=\frac{TN}{TN+ FP}$$

**Table 1:** Confusion matrix for the classification according to signal detection theory (SDT) and epidemiology

		Screening test states:	
		Headphone user	Loudspeaker user
True condition:	Headphone user	hit or TP	miss or FN
	Loudspeaker user	false alarm or FP	correct rejection or TN

Sensitivity and specificity are measures of intrinsic accuracy to screening tests and are considered constant and independent of prevalence (Zhou et al., 2011). For a test with more than one item or trial, the measures depend on the threshold: A lower threshold increases sensitivity and decreases specificity compared to a higher threshold in general (Treat & Viken, 2012, pp. 727–728).

Prevalence is defined as the proportion of people who have a particular disease or condition at a specific time (Rothman & Greenland, 2014). In our application the prevalence or base rate expresses the proportion of potential headphone users in a population. Let *π* denote the prevalence. In this case, the probability of randomly drawing a headphone user from the respective population of headphone and loudspeaker users equals* π* (see Eq. 3).

3 $$\textrm{Prevalence}=P\left(\textrm{headphones}\right)=\pi =\frac{TP+ FN}{TP+ FN+ TN+ FP}$$

The sensitivity, specificity, and prevalence can be used to calculate the *positive predictive value* (*PPV*) and the *negative predictive value* (*NPV).* Both values describe the conditional probability that the true state matches the respective result given the test result (Kestenbaum, 2019, p. 163; Miller, 2014, p. 768). In our application, the value expresses the probability of headphone usage when the screening test is positive (*PPV*) and the probability of loudspeaker usage when the screening test is negative (*NPV*). The predictive values are not inherent to the screening tests, as they are influenced by the prevalence. See Eq. (4) as well as Eq. (5) for the calculation (Fletcher & Fletcher, 2005, p. 39; Kestenbaum, 2019, p. 164).

4 $$\begin{array}{r}PPV=P\left(\mathrm{headphones}|\mathrm{test}\ \mathrm{positive}\right)=\frac{TP}{TP+ FP}\\=\frac{Sen\times \pi }{Sen\times \pi +\left(1- Spe\right)\times \left(1-\pi \right)\ }\end{array}$$

5 $$\begin{array}{r}NPV=P\left(\textrm{loudspeaker}|\textrm{test}\ \textrm{negative}\right)=\frac{TN}{TN+ FN}\\=\frac{Spe\times \left(1-\pi \right)}{\left(1- Sen\right)\times \pi + Spe\times \left(1-\pi \right)}\end{array}$$

Similar to the predictive values, the *overall utility* can be used to describe the performance of a test for a given prevalence. *Utility* is a “value placed on a specific decision-making outcome” corresponding to its desirability (Treat & Viken, 2012, p. 725). The overall utility (*U*_overall_, see Eq. (6); Treat & Viken, 2012, p. 736) describes “a utilities-weighted sum of the probabilities of the four decision-making outcomes” (Treat & Viken, 2012, p. 725). After choosing appropriate utilities (0 ≤ *U*_TP_, *U*_FN_, *U*_TN_, *U*_FP_ ≤ 1), the value can be used to select the “best” test, the one with the highest overall utility among several tests for the application (one test with different threshold values or different tests).

6 $$\begin{aligned}\begin{array}{c}{U}_{\text{overall}}=P(TP)\times {U}_{\text{TP}}+P(FN)\times {U}_{\text{FN}}\\+P(TN)\times {U}_{\text{TN}}+P(FP)\times {U}_{\text{FP}}\\ {U}_{\text{overall}}=\pi \times Sen\times {U}_{\text{TP}}+\pi \times \left(1- Sen\right)\times {U}_{\text{FN}}\\+\;\left(1-\pi \right)\times Spe\times {U}_{\text{TN}}+\;\left(1-\pi \right)\times \left(1- Spe\right)\times {U}_{\text{FP}}\end{array}\end{aligned}$$

For the common goal of maximizing the percentage of correct classifications, the weights would be *U*_TP_ = *U*_TN_ = 1 for correct classifications and *U*_FN_ = *U*_FP_ = 0 for incorrect classifications (Treat & Viken, 2012, p. 736).

### Existing screening tests

There are only a few screening tests to determine headphones or loudspeaker playback. Woods et al. (2017) developed a now widely used screening test based on destructive interferences. For this test, the used stimuli are based on a 200-Hz sinusoidal tone, which differ in terms of their level (normal level and low level) or phase between the two stereo channels (normal level but phase-shifted). For one trial of the test, all three stimuli are played sequentially. When reproduced via stereo loudspeakers, the level of the phase-shifted sinusoidal tone (one out of the three stimuli) drops compared to the other stimuli due to destructive interferences. The participant’s task is to name the softest one of the three tones. Ideally, when played back over loudspeakers, the phase-shifted tone is selected as the softest. When listening over headphones, the participant should select the low-level tone as the softest. There are a total of six trials for the complete screening procedure. If at least five out of six times the low-level tone was selected, a headphone playback is assumed. A more detailed description of the test can be found in the section *Method – Main Study*. Unfortunately, the study by Woods et al. (2017) lacks an appropriate measurement theory like SDT and, therefore, no information on sensitivity and specificity was reported. The accuracy of the screening process of Woods et al. (2017) remains vague. Moreover, the sample size was relatively small (*N* = 20 for each loudspeaker and headphone group). The characteristics of the screening procedure should be determined with state-of-the-art methods on a larger sample and with a bigger variety of playback devices.

More recently, another approach of screening for headphones playback was developed by Milne et al. (2021). The procedure is based on the perception of dichotic pitch (Huggins Pitch; see Cramer & Huggins, 1958). The stimulus consists of white noise presented on both the left and right channels. On one channel, the white noise is phase-shifted (180°) over a narrow frequency band. A tone embedded in the noise is perceived when played back over headphones but not when played back over loudspeakers. Milne et al. (2021) reported a sensitivity of 85% and a specificity of 70% for a test length of six trials and a threshold of five out of six correct responses. For reasons of comparison, Milne et al. (2021) also collected data on the Woods et al. (2017) screening method and calculated a sensitivity of 86% and specificity of 58% for the same threshold of five out of six correct responses.

### Evaluating data quality after applying screening tests

Sensitivity and specificity are important parameters usually used for the evaluation of screening tests (Kestenbaum, 2019; Newman, 2001). However, for the evaluation of screening results from Internet studies, this approach alone may be insufficient: Sensitivity and specificity are calculated based on a verified proportion of events (e.g., headphones and loudspeakers), but in Internet studies, the base rate of playback devices is unknown for a population. The results of a test should not be considered independent of the prevalence. A short example shows the importance of including prevalence when interpreting screening results: A headphone screening method with a sensitivity of 90% and a specificity of 90% is used to collect a data set with headphones-users only. As soon as 100 cases classified as headphone users have been collected, the study is stopped. Sensitivity (true positive rate) and specificity (true negative rate) are inherent to the screening test. The main question is which proportion of the screening results is caused by errors of the screening and which proportion is caused by using specific playback devices. To adequately assess the data quality, researchers must use a measure such as *PPV* (Eq. 4) and *NPV* (Eq. 5), which includes prevalence (not inherent to the screening test; Kestenbaum, 2019). In the above case of 100 headphone users, we could use *PPV* (Kestenbaum, 2019, p. 164) to reveal the probability that headphones were used given that the screening method states the use of headphones. Assuming that headphones were used by 18% (prevalence) of all participants who took part in the screening test, the PPV would be about 66%.

That means if the test states that headphones were used, the probability that this statement is correct would be 66%. Therefore, the expected value for true headphone users in the hypothetical sample is *n* = 66. The expected value for true loudspeaker users amongst the participants is *n* = 34 even though the screening test identified all subjects as headphone users. This calculation example is extreme since it assumes a blunt screening without requesting headphone usage from participants, resulting in a worst-case scenario for the prevalence. It demonstrates that the prevalence has a dramatic impact on data quality even in screening tests with high sensitivity and specificity. In other words, for any screening method, reliable information on the prevalence of a feature in the target population is of central importance and must always be taken into account for a meaningful interpretation of findings. As a main challenge, knowing the percentage of verified headphone users in a screening test is crucial. To the best of our knowledge, information on the prevalence of playback devices and participants’ behavior when certain playback devices are requested is currently unavailable. Therefore, it cannot be estimated which proportion of a sample was rejected in earlier studies due to loudspeaker use or due to the inherent error of the screening method itself (economics of the screening). For the same reason, no conclusion could be drawn as to how many true headphones-users were in the group of participants who were classified by the test as headphone-users (data quality). At first glance, this approach seems to be counterintuitive as our intention was to use the screening test for the identification of headphone users. Even with information on prevalence of playback devices, there is no easy-to-use strategy for including playback device base rates in the preliminary considerations. However, we will make suggestions for the reliable estimation of the prevalence based on empirical evidence.

### Screening strategies

Screening strategies are application methods that can improve the ability of screening tests. For example, strategies can be used to avoid response bias and to increase the number of potentially suitable participants. When headphones are required in Internet experiments, there are basically two strategies to gain control over the playback device used.

#### Filtering without Request (FWR)

For this screening strategy, the playback device used by the participants has to be recorded either via self-report or screening test. The required playback device for the study has to be concealed to prevent response bias. Based on the self-report or screening result, the participants can be grouped into headphone users (desired playback device – H) and loudspeaker users (undesired playback device – L). If a sample is expected to have a low headphone prevalence, for example, 25%, it is not wise, when screening for headphones, to exclude participants based on self-report as the proportion of available headphone users will never exceed the prevalence. The data quality may be high, but so is the number of excluded subjects. In some cases, it may become impossible to achieve a certain minimum number of participants. When a screening test is used, a low PPV would be expected due to the low prevalence. A hypothetical test with both a high sensitivity and specificity of 90% would lead to PPV of 75%. The data quality can therefore be described as poor.

#### Filtering after Request (FAR)

A more economical strategy is to request a specific device and to screen for compliance. At first, all participants are required to use headphones. It can be expected that some loudspeaker-users switch to headphones. Afterwards, a screening test can be applied. The participants can be filtered based on their screening result. The biggest problem with this method is that the true initial prevalence is unknown. In addition, it is difficult to estimate how many people actually switched to headphones. This seems contradictory, since the point of a playback device screening is to determine the rate of headphone and loudspeaker users. However, to determine the PPV, an estimate of the headphone rate is necessary. Otherwise, the data quality cannot be evaluated.

Both Strategy 1 (FWR) and 2 (FAR) were used in several Internet-based studies (Brown et al., 2018; Lavan et al., 2019; McPherson et al., 2020; Mehr et al., 2018; Niarchou et al., 2022; Ramsay et al., 2019; Tzeng et al., 2021; Woods & McDermott, 2018; Zelechowska et al., 2020).

### Study aims

From the challenges and problems elucidated above, we derived the following main study aims: In a first laboratory pre-study, we developed screening tests to detect headphone and loudspeaker playback. The aim was to check the general function of the tests under controlled laboratory conditions. Based on the knowledge gained, the tests’ length was then to be adjusted if necessary to improve the test characteristics.

In a second Internet-based main study, the improved screening tests were checked on the basis of more data and with a wider variety of playback devices. Furthermore, we wanted to gain reliable data on the Woods et al. (2017) screening test. In addition, we collected information on headphone prevalence. On this basis, parameters were calculated to evaluate screening tests and to develop screening strategies.

We wanted to develop a comprehensive method for planning and conducting playback device screening in Internet experiments by bringing all information and parameters of screening tests together in an online tool. Researchers can use this tool to select suitable screening tests and tailor optimal test combinations and thresholds for a specific use case. The overall approach makes it possible to estimate the required sample sizes and the data quality for the application of screening tests. This has a big advantage over the selection of single screening tests solely on the basis of sensitivity and specificity as this improves both the economics of the study and the knowledge about the data quality. In addition, a method to combine more than two screening tests was developed. Moreover, the screening tests are integrated into a common procedure (HALT Part I and Part II), which enables standardized conditions for testing playback devices.

## Method – Pre-study

### Experimental setup and procedure

As in HALT Part I (Wycisk et al., 2022), HALT Part II was meant to perform in ordinary non-optimized listening environments and with sound devices of diverse quality. For that reason, the laboratory experiment took place in a non-optimized laboratory room of the *Hanover Music Lab* (HML; for details, see Tables S1, S2 and S3 in the Supplemental Material) with a variety of low- to average- and high-quality transducers:

- Beyerdynamic DT 770 Pro 250 Ohm, closed circumaural, high-quality headphones;
- No-name earbuds, open, intra-aural, low-quality headphones;
- A pair of Yamaha HS8M loudspeakers (near field monitor) of average quality;
- Apple MacBook Pro, 13” (Retina, early 2015) low-quality loudspeakers/laptop.

The assigned quality level in this study is only a subjective classification. As in HALT Part I (Wycisk et al., 2022), we used the browser-based survey platform *SoSci Survey* (www.soscisurvey.de; Leiner, 2020) for the data collection in the laboratory. After giving demographic information, participants started with the above-mentioned average-quality loudspeaker condition, followed by the laptop, high-quality headphones, and low-quality headphones (see Fig. S1 in the Supplemental Material for the procedure). During the experiment, the experimenter and the participant were located in two separate rooms. Volume levels were monitored and recorded by the experimenter’s use of a second screen (split screen extension of the participants computer). Each listening session lasted approximately 90 min, including instructions, pauses and retests.

### Stimuli and task development

We developed stimuli and associated tasks to detect headphone and loudspeaker playback. All stimuli were created on an Apple MacBook Pro*,* 13*”* (Mid 2012) using Logic Pro X. In general, researcher-developed stimuli were limited to – 0.5 dBFS (true peak) to avoid clipping through the Gibbs phenomenon (Oppenheim & Schafer, 2014). Two different stimulus types were used in developing screening tests A and B for the identification of headphone and loudspeaker users.

#### Test A

**Fig. 1:**
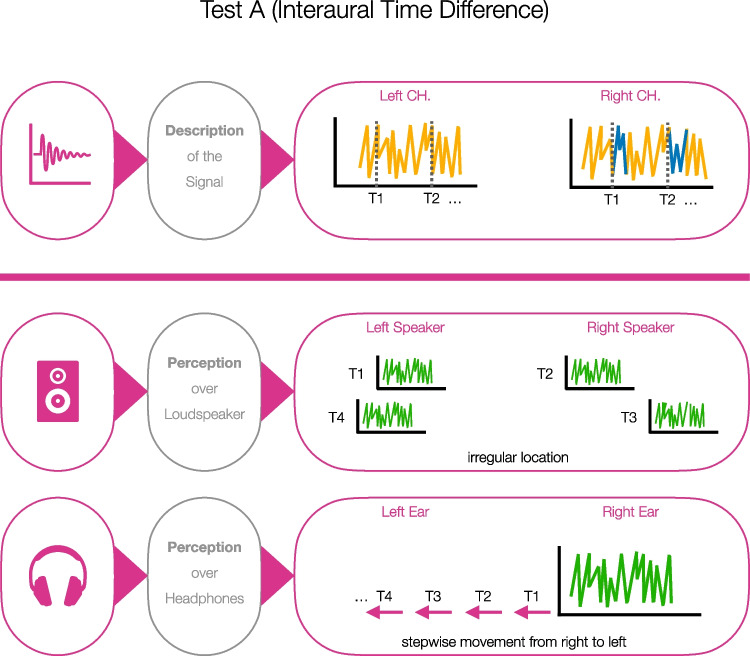
Stimulus description and perceptual correlates for the headphone screening based on interaural time differences (Test A). *Note.* Perception over loudspeaker: Noise jumping irregularly from one side to the other and back again (T1: noise on the left, T2: noise slightly on the right, T3: noise further to the right, T4: noise on the far left). Perception over headphones: Noise that moves stepwise from one side to the other. The example in the figure shows a movement from right to left

The first stimulus was based on *interaural time differences* (ITD) and was extracted from a CD with examples of dichotic pitch (Bilsen & Raatgever, 2002). For an illustration of the basic stimulus construction, see Fig. 1. In this case, there was identical continuous noise on the left and right channel. At the beginning of the stimulus, both channels had a time offset of 40 samples (0.907 ms). The right channel was ahead of the left channel. In other words, a specific section of the noise would first sound on the right channel and after 40 samples (0.907 ms) on the left channel. In intervals of 1 s, the continuous noise on the right channel was gradually but slightly delayed in eight steps (see Fig. 1, T1, T2, …). At each step, a time delay of ten samples (0.227 ms) was added. The resulting gap was filled with noise. Counting the initial offset plus the eight steps of delaying the signal, there are nine different segments within a stimulus. At the end of the stimulus, the right channel was 40 samples behind the left channel (0.907 ms). In general, if the stimulus is played back over headphones, the best-case perceptual correlate is a noise that moves stepwise from right to left (i.e., this response will be classified as a hit in terms of SDT). If the audio sample is played back over loudspeakers, then ideally the impression of a noise jumping irregularly from one side to the other and back again would be generated (i.e., correct rejection). Figure 1 illustrates a possible perception over loudspeaker (T1: noise on the left, T2: noise slightly on the right, T3: noise further to the right, T4: noise on the far left) In order to create an independent but comparable second stimulus, we swapped left and right channels. The final test included four trials to allow thresholds to be set. We labelled this screening approach Test A.

#### Test B

**Fig. 2:**
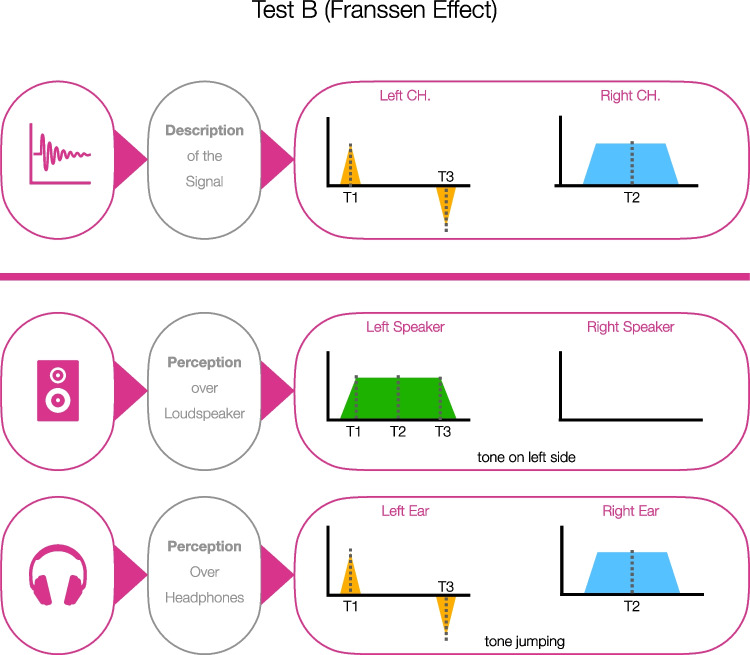
Stimulus description and perceptual correlates for the headphone screening based on the Franssen effect (Test B). *Note.* Perception over loudspeaker: Pure tone on the left side. Perception over headphones: Pure tone jumping from one side to the other and back again

The second stimulus was based on the Franssen effect (Ballou, 2008; Franssen, 1960), an auditory illusion related to the precedence effect (Plack, 2010). In general, the stimulus consisted of two short transient tones on one channel and a sustained tone on the other one. For an illustration of the basic stimulus construction, see Fig. 2. All characteristics of the stimulus were taken from Hartmann and Rakerd (1989). The left channel had a pure tone of 1 kHz with a total duration of 32 ms. During the first 2 ms, the level was constant. Immediately after the constant segment, the tone began to decay exponentially (see Fig. 2, T1). Simultaneously with the beginning decay on the left channel, the same sharp-onset (but sustained) sinusoid tone was begun on the right channel with a fade in of 30 ms (total duration = 1998 ms, Fig. 2, T2). At the end of the sustained tone, it started to decay exponentially over a period of 30 ms while a short sharp onset tone on the other (left) channel (total duration = 30 ms) increased exponentially with a phase shift of 180° over a period of 30 ms (Fig. 2, T3). The total duration of the audio was 2 s. The task was to identify the perceived channel of the pure tone. In the case of headphone usage, ideally the pure tone would jump from one side to the other and back again (i.e., this response is classified as a hit). In the case of loudspeaker use for playback, ideally the sound source of the pure tone would be perceived as being the left speaker (i.e., correct rejection). Here, too, the right and left channels were swapped to create a second comparable stimulus. Thus, the perception was the same – only the sides were changed. Again, the final test included four trials to allow thresholds to be set. We called this screening method Test B.

### Pretest for pre-study

The aim of the pre-study was to check the functionality of the newly developed screening tests. To identify issues regarding the study design of the pre-study, the HALT Part II procedure was pretested by students and laboratory assistants. As a result, we could observe that randomization of the procedure might influence the participants' responses due to the Franssen effect. Presumably, the headphone condition revealed the true nature of the stimulus composition of screening Test B. Thus, we decided against the randomization of the playback conditions. The final playback condition order was loudspeakers (1st), laptop (2nd), high-quality headphones (3rd), and low-quality headphones (4th).

### Participants

The study was conducted in June and July 2020. Participants were acquired through university mailing lists, advertising posters with a QR-Code and social media posts. A total of *N* = 40 participants (mean age = 31.83 years, SD = 13.48, *n* = 15 were males) took part in the study and gave written informed consent. The study was performed in accordance with relevant institutional and national guidelines (Deutsche Gesellschaft für Psychologie, 2016; Hanover University of Music, Drama and Media, 2017) and with the principles expressed in the Declaration of Helsinki. Formal approval of the study by the Ethics Committee of the Hanover University of Music, Drama and Media was not mandatory, as the study adhered to all required regulations.

According to self-disclosure, 35 participants reported normal hearing whereas five participants indicated a hearing loss (e.g., tinnitus, perception of noise). Additional screening to identify hearing loss is not always possible in Internet experiments. Since hearing loss can always be present in participants, we have followed a conservative strategy and decided not to exclude participants with hearing loss. We believe that this approach allows for a more realistic assessment of screening test performance because underachieving people are represented in the data. None of the participants used a hearing aid. Each participant was paid €15 as reimbursement for participation.

### Data analysis

Test A (based on ITD) and Test B (based on the Franssen effect) were used to check for headphones or loudspeaker playback. For the analysis, we examined the properties of individual Tests A and B and their combinations. As combination approaches for parallel tests, we used the A AND B method (which classifies a participant as a headphone user when both tests are positive and results in a decreased sensitivity and increased specificity) and the A OR B method (which classifies a participant as a headphone user when at least one test is positive and results in an increased sensitivity and decreased specificity; Cebul et al., 1982).

In addition to the aforementioned measures of diagnostic accuracy, we used receiver operating characteristic (ROC) curves from SDT in which the hit rate is plotted against the false-alarm rate for each threshold, and the area under the curve (AUC; Treat & Viken, 2012, pp. 731–735) was calculated. The AUC is independent of a chosen threshold and can be interpreted as the probability that a randomly selected pair of a headphone user and a loudspeaker user will be classified correctly by the test. Additionally, score confidence intervals according to Agresti and Coull (1998) were calculated for sensitivity and specificity (see Table 1 for details).

The individual and combined evaluations of the tests provided several parameters. To assess those parameters, we defined general target criteria for a useful test: 1. Data Quality – The probability of correctly detecting loudspeaker users should be high (high specificity). In other words, in order to ensure high data quality, only a small number of loudspeaker users should be classified as headphone users. 2. Economy – The probability of correctly detecting headphone users should be high (high sensitivity). In other words, to ensure the economics of the study, only a small number of headphone users should be classified as loudspeaker users. However, there might be conflicts in the selection of an adequate screening test. For example, a test with the highest data quality could produce many *misses* so that the target number of subjects would not be achieved.

## Results and discussion – Pre-study

The characteristics for Test A and Test B show that only sensitivity or specificity achieved a high value. No test scored high on both parameters at the same time (see Table 2 for details). The thresholds in Table 2 refer to the four trials for each test. The AUC from the ROC analysis was .642 and .735 for Test A and for Test B, respectively. Thus, the discriminative power of the individual tests could be considered mediocre at best.

**Table 2:** Characteristics of the screening procedures depending on different thresholds in the pre-study

Test	Threshold	Sensitivity [95% CI]	Specificity [95% CI]
A	≥ 1	96.3 [89.6–98.7]	16.3 [9.80– 25.9]
	≥ 2	96.3 [89.6–98.7]	22.5 [14.7– 32.8]
	≥ 3	93.8 [86.3–97.3]	28.8 [20.0– 39.5]
	= 4	83.8 [74.2–90.3]	42.5 [32.3– 53.4]
B	≥ 1	57.5 [46.6–67.7]	81.3 [71.4– 88.3]
	≥ 2	51.3 [40.5–61.9]	91.3 [83.1– 95.7]
	≥ 3	43.8 [33.4–54.7]	98.8 [93.3– 99.8]
	= 4	41.3 [31.2–52.2]	100.0 [95.4–100.0]

CI = Confidence interval in %, *N* = 40

To calculate the characteristics of test combinations directly from the characteristics of individual tests, the tests have to be statistically independent conditional on the true state (Cebul et al., 1982; Zhou et al., 2011, p. 409), that is, the device used. The tests are statistically independent conditional on the true playback device if *P*(A = a|B = b, d) = *P*(A = a|d) for all *a*, *b* ∈ {0, 1} and *d* ∈ {headphones, loudspeakers}. Each of the 16 possible combinations of test A and B was checked for conditional independence by using a chi-square test or an exact multinomial test where assumptions of the former were violated (Bortz & Lienert, 2008, pp. 72–76; Bortz & Schuster, 2010, pp. 142–143) at an α-level of .10 (for details, see Table S4). Since the null hypothesis of these tests was of interest, we chose the comparatively higher α-level to protect against the β-error. The characteristics of all combinations of Test A and B could be calculated from their individual characteristics (for details, see Table S5 in the Supplemental Material). In general, either the sensitivity or the specificity of tests A and B as well as their combinations were relatively low. Unfortunately, comparing the mentioned parameters alone was insufficient for the selection of a method. The initial decision, for example, about whether headphones or loudspeakers should be used in a study affects whether sensitivity or specificity become important to evaluate data quality and economics of a screening method. Additionally, the estimated quality of the data would be influenced by the prevalence of the required playback device in the target sampling group. All factors together influence the total number of participants who have to be invited in order to achieve the desired sample size of participants with the verified playback device. We decided to conduct an online study (Main Study) to address those problems by extending the length of the screening procedures (thus, improving test performance) and collecting data on prevalence (evaluating real-life application).

## Method – Main study

To gain more knowledge about the fundamental question of the likely headphone prevalence in a sample and to calculate the characteristics of the screening methods on a larger database and with a wider variety of playback devices, we conducted an Internet study.

### Experimental setup and procedure

SoSci Survey (Leiner, 2020) was used as a browser-based survey platform (www.soscisurvey.de) for collecting the sample’s response data via Internet. HALT Part I (Wycisk et al., 2022) was implemented to control for playback characteristics and standardize loudness adjustments. Each session lasted approximately 15 min. A cover story was used to disguise the purpose of the survey. This strategy was essential so that we could determine the unbiased prevalence of the individual playback devices used. Several safety precautions and screen-out methods were implemented to avoid data confounding. The length of processing was measured for each page. Minimum (5, 10, 12 s) and maximum screen-out criteria (60, 120, 180, 300 s) were defined depending on the questionnaire page. To increase the participants' awareness for the registration of processing time, we implemented a time loop right before the main questionnaire pages started. In case the time loop page was passed too fast, the information was prompted that the criterion for a minimum processing duration was not fulfilled (see flowchart of the exclusion procedure in the Supplementary Material, Fig. S2). The time loop page was then repeated (as often as necessary). Based on this method, we could also prevent the use of rapid autofill scripts (https://help.alchemer.com/help/use-autofill-javascript-to-save-time-taking-surveys; Domagalski, 2020).

An additional strategy to filter for similar scripts was to leave the input field for “age” open to any number of digits. For example, it was possible to enter the age of 999. All participants who entered the questionnaire link were prefiltered by the panel provider regarding their age (18–60 years). Pretests had shown that the aforementioned autofill script could not produce content-appropriate input for a question. Based on these precautions, every age entry that fell below or exceeded our requested range (18–60 years) was filtered. To check for the participants' attention, an instructed response item (Leiner, 2019; “Answer this item with the scale step ‘strongly disagree’”) was embedded into the research items. An incorrect response resulted in a screen out. In addition, we screened out people with hearing aids, self-reported hearing loss, problems with right-left discrimination, users of smartphones/tablets/monitors/TVs as a playback device, and subjects with interchanged stereo channels or mono playback. To control for unknown hearing loss, we used the Quick Hearing Check (QHC; Kochkin & Bentler, 2010).

### Stimuli

The same screening tests A and B were used as in the pre-study. However, for each of the tests, the number of trials was increased to six (before: four) to increase their diagnostic accuracy. Additionally, a complete version of Woods et al.’s (2017) screening procedure (six items) was added, which will be referred to as Test C in the following.

**Fig. 3:**
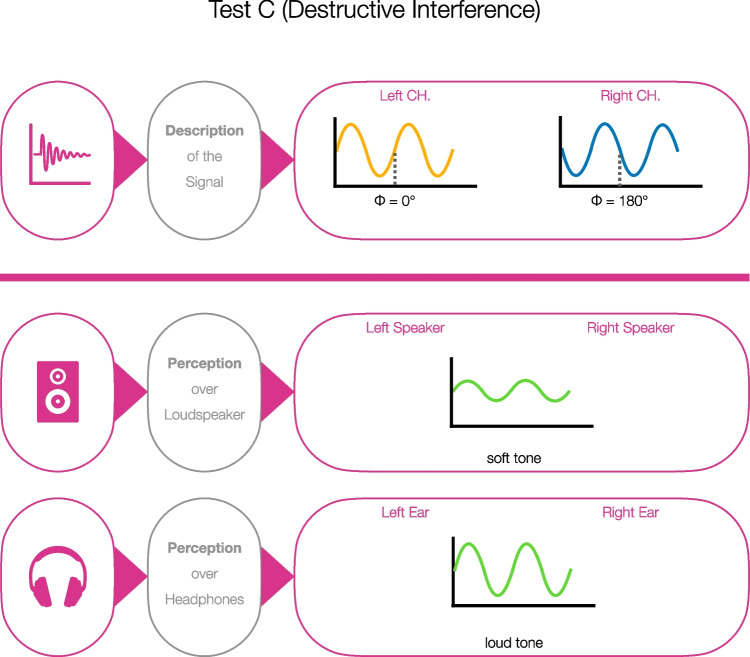
Stimulus description and perception for the headphone screening based on destructive interference (Test C) by Woods et al. (2017). *Note.* Perception over loudspeaker: The tone with phase-shifted channels will be perceived as softest. Perception over headphones: The tone with phase-shifted channels will be perceived as loud

Woods et al.’s (2017) test is based on an intensity-discrimination task (see Fig. 3). Three different stimuli were created by using a 200-Hz sinusoidal tone. The first stimulus was unmodified and, therefore, could be called the standard. A second stimulus used a lower gain (– 6 dB) compared to the first standard stimulus. The third stimulus had the same gain as the first stimulus, but one channel was phase-shifted by 180°. All stimuli differed in terms of their level or the phase between the two stereo channels. After presenting the three stimuli successively, we asked the participants to decide which stimulus (first, second, third) was the softest. In total, the task was presented six times using a randomized stimulus sequence. In the case of headphone usage, the stimulus with the lowered gain (second) would be perceived as the softest (i.e., hit). If the stimuli were reproduced via stereo loudspeakers, the level of the phase-shifted sinusoidal tone (third) would drop compared to the other stimuli (i.e., correct rejection).

### Participants

The study was conducted in November and December, 2020. Participants were invited by an external panel provider (mo'web, https://www.mowebresearch.com). In total, 1,545 people took part in the study. After exclusion of participants according to the various aforementioned filter criteria, *N* = 211 valid cases remained (mean age = 42.40 years, SD = 11.35, *n* = 117 were females; for details on participant exclusion, see Fig. S2 in the Supplemental Material) According to the QHC inventory, *n* = 8 participants reported an unknown moderate to severe hearing loss and *n* = 1 an unknown severe to profound hearing loss. Each participant gave informed written consent and received a small gratuity from the panel provider after successfully finishing the survey.

### Playback devices used by participants

HALT Part I (Wycisk et al., 2022) was used to control characteristics of playback devices used by the participants. As indicated by the self-report, the majority of valid participants (*N* = 211) used a laptop (*n* = 102 [48.34%]) as a playback device, followed by headphones (*n* = 80 [37.91%]) and freestanding loudspeakers (*n* = 29 [13.74%]). The headphone users could be further divided into circumaural (*n* = 20 [9.48%]), intra-aural (*n* = 37 [17.54%], earbuds and in-ears), supra-aural (*n* = 16 [7.58%]), and 7 (3.31%) unknown types. As we excluded all subjects (*n* = 187) using mono playback, swapped channels, and uninterpretable input during the stereo test, 100% of the valid cases (*N* = 211) used in the analysis fulfilled the criteria for stereo playback and correct channel assignment. The test for frequency limits revealed that more than 80% of the participants could hear frequencies of 100 Hz and above. As in the laboratory study, there was a peculiar result among laptop users: Of the participants, *n* = 21 gave correct responses for the counting task at 20 Hz. However, from the physical perspective, this seemed to be an unrealistic finding because frequencies of this very low range cannot be reproduced by laptop speakers. Thus, as in the laboratory study, we assumed that these artifacts were produced by the audio processing and transmission system of the laptops themselves during playback. Details for all playback devices regarding the frequency limits can be found in Table 3.

**Table 3:** Lower frequency limits of the self-reported playback devices in the main study (Internet)

Transducer type	20 Hz	60 Hz	100 Hz	140 Hz
headphones	25 (31.3%)	72 (90.0%)	77 (96.3%)	72 (90.0%)
loudspeakers	8 (27.6%)	22 (75.9%)	27 (93.1%)	24 (82.8%)
laptops	21 (20.6%)	38 (37.3%)	68 (66.7%)	78 (76.5%)
overall	54 (25.6%)	132 (62.6%)	172 (81.5%)	174 (82.5%)

The table shows the absolute and relative frequencies of participants that could hear the frequencies 20/60/100/140 Hz indicated by HALT Part I and segmented by self-reported playback devices. E.g.: 22 (75.9%) of the loudspeaker users could hear the frequency 60 Hz

### Data analysis

#### Combination of screening tests

The main objective was to combine the three screening tests and to calculate the corresponding parameters. The following considerations form the basis of the analysis. As a reminder, each individual test consists of six trials. Threshold values can be set for each test individually. In the following, the threshold values are not considered for the time being, but only the logically possible results. If 1 stands for headphones and 0 for loudspeakers, then combining two screening tests A and B produces four possible outcome pairs regardless of the truth:

$$\begin{aligned}&\left(\textrm{A}=1,\textrm{B}=1\right),\left(\textrm{A}=1,\textrm{B}=0\right),&\\&\left(\textrm{A}=0,\textrm{B}=1\right),\left(\textrm{A}=0,\textrm{B}=0\right)\end{aligned}$$

Combining three screening tests A, B and C produces eight possible outcome triples regardless of the truth:

$$\begin{aligned}\left(\textrm{A}=1,\textrm{B}=1,\textrm{C}=1\right),\left(\textrm{A}=1,\textrm{B}=1,\textrm{C}=0\right),\\\left(\textrm{A}=1,\textrm{B}=0,\textrm{C}=1\right),\left(\textrm{A}=1,\textrm{B}=0,\textrm{C}=0\right),\\\left(\textrm{A}=0,\textrm{B}=1,\textrm{C}=1\right),\left(\textrm{A}=0,\textrm{B}=1,\textrm{C}=0\right),\\\left(\textrm{A}=0,\textrm{B}=0,\textrm{C}=1\right),\left(\textrm{A}=0,\textrm{B}=0,\textrm{C}=0\right)\end{aligned}$$

Using machine learning methods for multiple classifiers (ensemble learning or rather ensemble methods), these pairs or triples can be used to form one global result *G*(*A*, *B*) or *G*(*A*, *B*, *C*) respectively, according to a voting combiner (Brown, 2017). Each screening test in an ensemble is assigned a weight, e.g., *ω*_*A*_ is the weight for Test A. These weights are uniform for a simple vote in which all tests are of equivalent value for the final classification, i.e., *ω*_*A*_ = *ω*_*B*_ = *ω*_*C*_, or non-uniform for a weighted vote (Brown, 2017). *G*(*A*, *B*, *C*) = 1, i.e., the global result is headphones, for *ω*_*A*_ · *A* + *ω*_*B*_ · *B* + *ω*_*C*_ · *C* ≥ 1 and *G*(*A*, *B*, *C*) = 0 otherwise, which stands for loudspeakers.

In a preparatory step, we compiled a list of test combinations, that is, assignments of weights to the three individual tests within a voting combiner. The individual tests, pairwise tests and also three-way tests were included. Only combinations where the final classification made sense were considered. All in all, there were 18 such combinations (3 individual tests, 6 pairwise tests, 9 three-way tests). A number was assigned to each one of them, which we called *evaluation key* (EK; for all combinations, see Table S6 in the Supplemental Material). Besides the weights, each combination can be described by logical statements: for example, “at least two times headphones” would be the statement describing the uniform weights ${\omega}_A={\omega}_B={\omega}_C=\frac{1}{2}$ (EK 11) whereas “(Test A OR Test B) AND Test C” describes the weights ${\omega}_A={\omega}_B=\frac{1}{4}$ and ${\omega}_C=\frac{3}{4}$ (EK 13). Note that “OR” and “AND” are logical operators here and therefore Test C is mandatory and Test A and Test B are not mutually exclusive for EK 13. Considering all possible thresholds for each test (reasonable thresholds ranging from one to six) together with the 18 combinations, there are 2178 screening methods (three individual tests with six thresholds each, six pairwise tests with 6 × 6 thresholds each, nine three-way tests with 6 × 6 × 6 thresholds each).

In a first step, the individual sensitivity and specificity (see Eqs. 1 and 2) as well as the score confidence interval (Agresti & Coull, 1998) were calculated for Tests A, B, and C at threshold values ranging from one to six. When the criterion for conditional independence was met for a test combination the probability of any outcome pair or triple was calculated from the values for the individual tests (Cebul et al., 1982; Zhou et al., 2011) and, thus, the sensitivity and specificity for this combination. Without conditional independence, the characteristics of a test combination were calculated from the data treating the combination as one individual test and using Eqs. (1) and (2).

## Results – Main study

### Prevalence – Empirical determination

In the main study, the prevalence of headphones was determined by trusted and unbiased self-report. This information enables a practical evaluation of possible screening methods. Along the line of Agresti and Coull (1998), we calculated the score confidence interval. Basically, two methods for the calculation of a base rate (Prevalence A and B) for headphones were used. To allow comparability with the results of the laboratory study, participants who used a smartphone, tablet, or monitor/TV for playback were filtered out (Prevalence A). Prevalence A (*N* = 211) was assumed as the unbiased base rate for headphones (37.92%, 95% CI [31.6%, 44.6%], *n* = 80) amongst valid cases after we applied all exclusion criteria to the data set (smartphones, tablets, monitors/TVs were not allowed as playback devices). Prevalence B (*N* = 1,194) was assumed as the unbiased base rate for headphones (17.67%, 95% CI [15.6%, 19.9%], *n* = 211) of all participants who reached the playback device filter (but before we applied the filter criterion to the data set), leaving smartphones, tablets, and monitors/TVs included. Therefore, Prevalence B also consisted of cases that would have been excluded for other reasons in the further course of the questionnaire.

### Evaluation of the screening tests

#### Individual screening tests

**Fig. 4:**
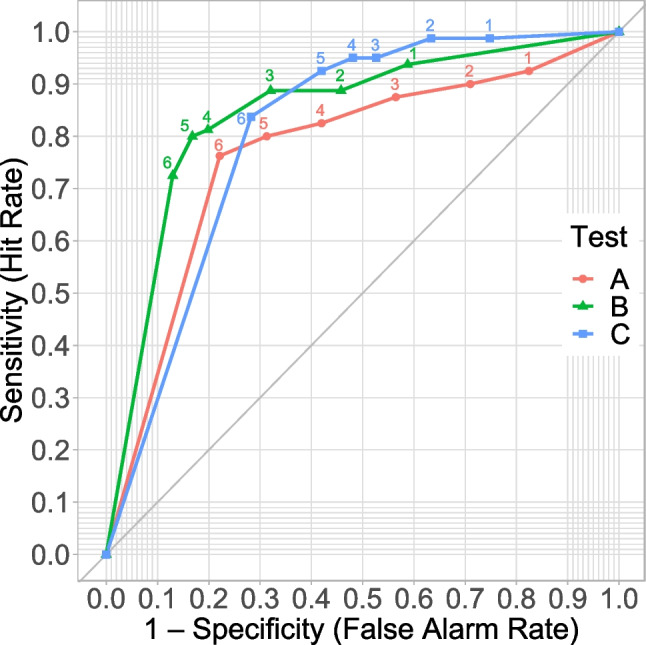
ROC curves. Test performances at all six thresholds (*N* = 211)

**Fig. 5:**
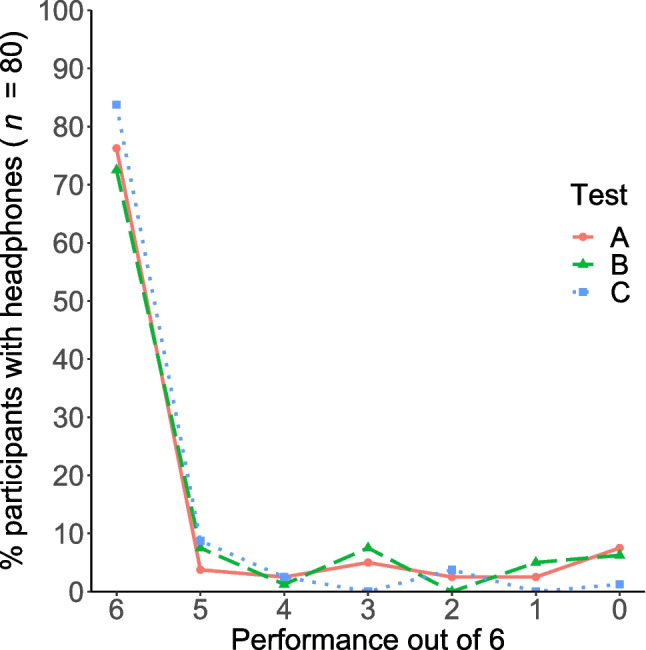
Performance distribution for Test A, B, and C in the self-reported headphone users (*n* = 80)

**Fig. 6:**
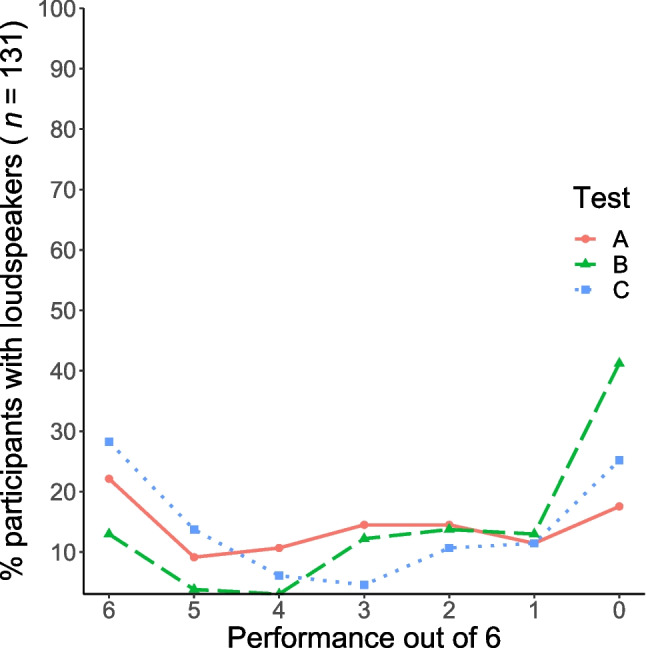
Performance distribution for Test A, B, and C in the self-reported loudspeaker users (*n* = 131)

Three different tests (Test A based on ITD, Test B based on the Franssen effect, and Test C based on destructive interference [Woods et al., 2017]) were used to check for headphone or loudspeaker playback. For all tests, we calculated the sensitivity and specificity. In accordance with Agresti and Coull (1998), we calculated the score confidence interval for both sensitivity and specificity (see Table 4). The ROC curves for the three individual tests (A, B, and C) are shown in Fig. 4. For Test A, we calculated an AUC of .768 and for Test B an AUC of .844. After comparing the AUC values of the pre-study with those of the main study, we successfully increased the discriminative power of Test A and B. For Test C, we calculated an AUC of .807. The performance distribution for each test is shown in Fig. 5 for headphones and in Fig. 6 for loudspeakers. Considering the confidence interval, our determined sensitivity (92.5 %) and specificity (58.0%) of the procedure by Woods et al. (2017) for a threshold of five out of six was similar to the results of Milne et al. (2021), with sensitivity = 86% and specificity = 58%. For comparison, Milne et al. (2021) reported a sensitivity of 85% and a specificity of 70% (test length of six tasks and a threshold of five out of six) for their procedure based on the Huggins Pitch. Additionally, an AUC of .821 was reported. In comparison, Test B showed a lower sensitivity (more misses), better specificity (higher data quality), and a higher AUC value (better overall test performance). However, at this point it would not be appropriate to speak in terms of a test’s superiority: The confidence intervals showed the uncertainties in the determined test characteristics. Rather, it should be mentioned that both Test B and the test by Milne et al. might complement each other very well in the future.

**Table 4:** Characteristics of the screening procedures in dependence of different thresholds (minimum of correct responses required) in the main study. *N* = 211

Test	Threshold	Sensitivity [95% CI]	Specificity [95% CI]
A	≥ 1	92.5 [84.6–96.5]	17.6 [12.0–25.0]
	≥ 2	90.0 [81.5–94.8]	29.0 [21.9–37.3]
	≥ 3	87.5 [78.5–93.1]	43.5 [35.3–52.1]
	≥ 4	82.5 [72.7–89.3]	58.0 [49.4–66.1]
	≥ 5	80.0 [70.0–87.3]	68.7 [60.3–76.0]
	= 6	76.3 [65.9–84.3]	77.9 [70.1–84.2]
B	≥ 1	93.8 [86.3–97.3]	41.2 [33.1–49.8]
	≥ 2	88.8 [80.0–94.0]	54.2 [45.7–62.5]
	≥ 3	88.8 [80.0–94.0]	67.9 [59.5–75.3]
	≥ 4	81.3 [71.4–88.3]	80.2 [72.6–86.1]
	≥ 5	80.0 [70.0–87.3]	83.2 [75.9–88.6]
	= 6	72.5 [61.9–81.1]	87.0 [80.2–91.7]
C	≥1	98.8 [93.3–99.8]	25.2 [18.5–33.3]
	≥ 2	98.8 [93.3–99.8]	36.6 [28.8–45.1]
	≥ 3	95.0 [87.8–98.0]	47.3 [38.9–55.8]
	≥ 4	95.0 [87.8–98.0]	51.9 [43.4–60.3]
	≥ 5	92.5 [84.6–96.5]	58.0 [49.4–66.1]
	= 6	83.8 [74.2–90.3]	71.8 [63.6–78.8]

CI = Confidence Interval

#### Combination of screening tests

In contrast to the pre-study, we now combined Test C (Woods et al., 2017) with Tests A and B to increase the diagnostic accuracy. To decide whether the characteristics of the combination of screening methods could be calculated directly from the individual characteristics of the tests A, B, and C, we checked those tests for conditional independence by using a chi-square test and an exact multinomial test in which assumptions of the former were violated (α = .10). In general, we found that the tests were not conditionally independent for thresholds greater than 2, given the use of headphones (for details, see Tables S7, S8, S9 and S10 in the Supplemental Material). Therefore, we treated each of the combinations as a single screening test and determined their characteristics according to Eqs. (1) and (2). Sensitivity and specificity for the 2160 screening procedures using more than one individual test are tabulated in the Supplemental Material Table S11. Among them are procedures with fairly high sensitivity and specificity. For example, the three-way test with uniform weights (“at least two times headphones”, EK = 11) and thresholds 5, 5, and 6 for tests A, B, and C, respectively, has a sensitivity of 83.75% and a specificity of 83.97%. Another high-valued procedure is the two-way Test B and C (EK = 6) with the thresholds 3 and 5 for Test B and Test C, respectively, which has a sensitivity of 86.25% and a specificity of 84.73%.

### Selection of a screening procedure and a screening strategy

Because sensitivity, specificity, and AUC do not take into account the prevalence of playback devices, and balancing the two values PPV and NPV is not a straightforward task, we suggest using either the overall utility with utility weights for maximizing the percentage of correct classifications or an approach that additionally accounts for the required sample size for the selection of a screening procedure and a screening strategy.

#### Considerations for the Strategies “Filter Without Request” (FWR) and “Filter After Request” (FAR)

Choosing the optimal screening test or combination of tests for the strategies FWR and FAR requires an estimate for the prevalence of headphone users in the target population. The estimate must be made separately for both strategies because a request (FAR) changes the base rate. The first way to select the “best” screening test or combination is to use the overall utility as described in the ‘Nomenclature, Definitions and Fundamentals’ section of the Introduction. Another approach takes the target sample size into account. Let us consider a screening test with known sensitivity and specificity as well as a sample of *n* persons with a positive result from that test. Let *H* denote the number of true headphone users in this sample. When the population from which the sample is drawn is not too small, we can conceptualize *H* as a random variable following a Binomial distribution with size *n* and a probability of success equaling the PPV for the prevalence estimate and the screening test used. Thus, the probability that the true number of headphone users equals *k* is given by Eq. (7) (Jacod & Protter, 2004, pp. 23, 24, 30).

7 $$P\left(H=k\right)=\left(\begin{array}{c}n\\ {}k\end{array}\right)\ {PPV}^k\ {\left(1- PPV\right)}^{n-k}$$

Let us consider the event that *k* or more persons were using headphones and let *ϑ* denote its probability:

8 $$\vartheta :=P\left(H\ge k\right)=\sum_{i=k}^n\left(\begin{array}{c}n\\ {}i\end{array}\right)\ {PPV}^i\ {\left(1- PPV\right)}^{n-i}$$

When we have conducted a study using FWR or FAR and one screening test or screening test combination, we can use Eq. (8) for a post hoc data quality estimation. Data quality would be *H* — or *k* as lower limit of *H* — divided by *n*. For a given *k* and thus a given data quality, we can compute its probability *ϑ*. Similarly, for a given *ϑ* we can use the quantile function of this binomial distribution to determine *k* and state that with a probability of *ϑ*, we have a data quality of at least *k* divided by *n*. Equation (8) also forms the basis for selecting a screening test or combination of tests a priori. Therefore, we set *k* to the desired number of headphone users, e.g., the sample size from an a priori power analysis, and *ϑ* to a minimum probability — or more colloquially a minimum certainty — and determined *n* for which *P*(*H* ≥ *k*) ≥ *ϑ*. To compute this *n,* we used a Normal approximation of the Binomial distribution with a continuity correction based on the De Moivre–Laplace theorem (see for example Georgii, 2004, pp. 129–135). For *ϑ* > 0.5, we get

9 $$n=-\frac{a}{2}+\sqrt{{\left(\frac{a}{2}\right)}^2-b}$$

where *a* = (−*PPV*^−1^(2*k* − 1 + (1 − *PPV*)(*ϕ*^−1^(1 − *ϑ*))^2^)) and *b* = *PPV*^−2^(*k* − 0.5)^2^ with *ϕ*^−1^ denoting the inverse cumulative distribution function of the standard normal distribution (for details, see Section S1 in the Supplemental Material). This can be done for all available screening tests and combinations. We then selected the screening procedure for which the smallest *n* was computed and thus the data quality would be maximized.

#### Considerations for a Split–Convince–Compare strategy

We propose another screening strategy that aims at increasing the number of participants with the target playback device. This is done by reducing the number of misses among participants who start a study with the target device and prompting the remaining participants to switch to the target device. We call this strategy *Split–Convince–Compare* (SCC).

- *Split*: At first, all participants have to be asked what playback device is being used. At this point, the desired device for the study must be concealed to avoid response bias. As conclusions can be drawn about the exclusion criteria when the desired device is disclosed, particular caution is required with paid participants from panel providers. Incentives are usually only paid once the questionnaire has been completed in full. Therefore, it is to be expected that test subjects from panel providers will try to avoid triggering exclusion criteria. After the playback device used was determined by self-report, all participants can be split up into two different groups: participants who were already using the desired playback device (D1) and participants who were not using it (D0). With this method, the unbiased base rate (prevalence) of headphones and loudspeakers in the sample can be estimated. Furthermore, the theoretical prevalence based on self-report for the desired playback device is assumed to be 100% in D1 and 0% in D0. When a large number of test persons, for example with headphones, has to be achieved, it is not always sufficient to only allow people from D1 (in this case headphone users) to take part in the study. We found a headphone prevalence B of 17.67% (95% CI [15.6%, 19.9%]). Therefore, it could be expected that only about 17.67% of all participants who started the questionnaire were using the desired device. Based on that, about 82.33% (100% − 17.67%) of the participants would have to be excluded if only subjects from D1 had been allowed to take part in the study.
- *Convince:* A better alternative is to convince all participants who were not using the correct playback device to use the desired device. The request must be addressed to all participants in group D0. It can be assumed that after the request, some participants will switch the device. Therefore, the prevalence in group D0 will increase to a value greater than 0%.
- *Compare:* After the desired playback device is requested, all participants have to complete a screening test with known sensitivity and specificity. This allows the experimenter to further divide both groups D1 and D0 in test positive and test negative. This results in four different groups: participants from D1 for whom the screening test says that they use headphones (D1^+^) or that they do not use headphones (D1^–^); participants from D0 for whom the test says that they use headphones (D0^+^) or that they do not use headphones (D0^–^). Obviously, these further classifications based on screening tests are subject to error as can be seen in D1^–^ where theoretically all participants are misclassified. But also, in D0^+^ and D0^–^ misclassifications will occur, and both groups will contain participants who switched to the target device and others who did not.

The final sample for the SCC strategy will consist of the group D1 assuming that the self-report was unbiased and the group D0^+^ accepting some misclassifications. Therefore, SCC outperforms FAR theoretically since the final FAR sample consists of the groups D1^+^ and D0^+^ whereas D1^–^ would be missing.

To select the “best” screening test or combination of tests for SCC, we need an estimate for the probability that a participant who indicates the use of a playback device other than headphones actually switches to headphones. Let *ς* denote this probability or switching prevalence for the target population and let $\hat{\varsigma}$ be its estimate. Since all participants in D1 will be included in the final sample, the “best” test or test combination has to optimally perform in group D0 where the prevalence is *ς*. Therefore, we now calculate the overall utility by replacing *π* with $\hat{\varsigma}$ in Eq. (6) and select the test with the maximum value. For the post hoc estimation of data quality when SCC was used, the two groups D1 and D0^+^ are first considered separately. D1 is assumed to have a data quality of 100%. For D0^+^ we calculate a PPV by replacing *π* with $\hat{\varsigma}$ in Eq. (4) and use Eq. (8) to estimate the data quality in this group. The data quality for the whole sample, specifically, D1 and D0^+^, is then the sum of the number of participants in D1 and *k* from the estimation for D0^+^ from Eq. (8) divided by the number of participants in D1 and D0^+^, and has a probability of *ϑ*. For an a priori sample size-based selection of a screening test or test combination within SCC, we use a similar approach to FWR and FAR. Again, let *H* be the number of true headphone users and be conceptualized as a random variable with Binomial distribution. The size of this distribution is then the number of participants in D1 and D0^+^ and has to be determined. The probability of success $\overset{\sim }{p}$ is the probability that a participant used headphones given that they indicated the use of headphones (member of group D1) or that they got a positive test result (member of groups D1^+^ or D0^+^). Therefore, estimates for the prevalence of the target device and the switching prevalence are required: $\hat{\pi}$ and $\hat{\varsigma}$, respectively. Again, we assume an unbiased self-report. Thus, the probability that a participant uses headphones and indicates this is $\hat{\pi}$. The probability that a participant uses headphones after being prompted is $\left(1-\hat{\pi}\right)\times \hat{\varsigma}$. Therefore, the probability of success is

10 $$\overset{\sim }{p}=P\left(\textrm{headphones}\ |\ \textrm{D}1\ \textrm{or}\ \textrm{D}{0}^{+}\right)=\frac{\hat{\pi}+\left(1-\hat{\pi}\right)\times \hat{\varsigma}\times Sen}{\hat{\pi}+\left(1-\hat{\pi}\right)\left(\hat{\varsigma}\times Sen+\left(1-\hat{\varsigma}\right)\times \left(1- Spe\right)\right)}$$

To compute *n*, the number of participants in D1 and D0^+^, we use Eq. (9) and substitute the PPV with the new probability of success $\overset{\sim }{p}$ (for details, see Section S2 and Fig. S4 in the Supplemental Material).

#### Online tool

To facilitate the selection of the best combinations of screening methods, a calculator was programmed that is available online (http://testing.musikpsychologie.de/HALTConfig/) and part of the HALT R package (the package can be retrieved from https://github.com/KilianSander/HALT). By entering the desired playback device and an estimated prevalence (amongst others), the calculator can select a screening method to either maximize the data quality (fewer false alarms) or the economics (fewer misses). Both a priori and post hoc calculations are possible. Additional information on how to use HALT and the calculator can be found on https://osf.io/3tks7/.

At this point we would like to give an application example. The best screening procedure should be found for the following requirements and preconditions:

- Screening strategy: SCC
- Target device: HP
- Minimum number of target device users *k* = 70
- Minimum probability (certainty) *ϑ* = 0.80
- Prevalence estimate $\hat{\pi}$ = 17.67% (Prevalence B)
- Switching prevalence estimate $\hat{\varsigma}$  = 55%

The online tool outputs the following test procedure as the best:

Test combination “all HP” (EK 12) with thresholds 6, 2, and 6 for Test A, Test B, and Test C, respectively, yields $\overset{\sim }{p}$ = 0.9614 and a sample size of 74 participants who either reported the use of headphones or got a positive test result after being prompted to use headphones. With a probability of *ϑ* = 0.80, at least 70 out of those 74 participants actually used headphones.

## Discussion

The results of our studies revealed three main components for the successful application of a screening test: a screening test with a high accuracy, information on prevalence of the required equipment (in our case headphones or loudspeakers), and a reliable screening strategy. In a first laboratory pre-study, we successfully developed two new playback device screening Tests A and B to control for headphone and loudspeaker usage. Based on an Internet survey (Main Study), we improved both tests, compared them to existing headphone screening procedures and collected data on headphone prevalence. Widespread screening strategies were discussed, and a new, superior strategy (Split Convince Compare – SCC) was suggested. Finally, due to the combination of the (a) newly developed screening calculator in conjunction with the (b) screening strategy and (c) inclusion of information on the prevalence of headphone use, we could provide valid tools for the control of playback devices in Internet studies.

Still, there are a number of issues to be considered. In general, more information is required on the biased prevalence (the true use of a certain playback device after it was requested) of terminal devices. Currently, it seems unclear how participants truly behave when they are asked to use a certain playback device. Furthermore, the screening tests need to be evaluated with a wider variety of devices that use built-in speakers. Tablets and smartphones are especially important in this context. Due to the size of the devices, the spatial proximity of the built-in stereo speakers is small. The properties of Tests A and B can, therefore, suffer. Built-in sound processing can also cause a confounding of test results. Test C (Woods et al., 2017), for example, may have been prone to certain sound processing. In devices with bass management and a crossover frequency of over 200 Hz, a complete cancellation can occur before the stimulus can be heard as an airborne sound. The perception component of the screening test would be lost as the stimulus is not physically present. This can lead to an overestimation of Test C’s properties. Additionally, dynamic level interventions of devices could falsify the level standardization with HALT.

In the future, the integration of the screening test by Milne et al. could help increase the overall performance of the combined screening tests. Finally, we hope that the suggested HALT procedures will contribute to improved data quality, efficiency, and overall study performance in Internet experiments on auditory perception. Data comparable to the quality of laboratory settings are the prerequisite for the future acceptance of Internet listening experiments.

## Summary

In this study (HALT – Part II), we developed a comprehensive screening procedure to detect headphones and loudspeakers. The complete HALT procedure with a duration of about 8 min consists of both Part I (Wycisk et al., 2022) and Part II. The procedure allows the standardization of loudness adjustments, the detection of stereo/mono playback, the assessment of lower frequency limits of playback devices, and the detection of headphone and loudspeaker playback (see Fig. S3 in the Supplementary Materials for the sequence plan of the complete HALT procedure). For a standalone demo version of HALT, please visit http://testing.musikpsychologie.de/HALT. For the use of HALT for research purposes, either the HALT R package can be downloaded from GitHub (https://github.com/KilianSander/HALT) or the DGM (Deutsche Gesellschaft für Musikpsychologie) DOTS (DGM Online Testing) ready-made online version is available (http://testing.musikpsychologie.de/dots_home/). To adapt the HALT to the needs of a specific study, the procedure can easily be set using a web interface (http://testing.musikpsychologie.de/HALTConfig/).

### Supplementary information

ESM 1 (PDF 539 kb)
