# Supplementary material for: The Headphone and Loudspeaker Test–Part II: A comprehensive method for playback device screening in Internet experiments
Source: Behav Res Methods. 2023 Jan 17;56(1):362–78. doi: 10.3758/s13428-022-02048-3 (PMC10794391; doi:10.3758/s13428-022-02048-3)
Supplement: Supplementary file 1 — (PDF 539 kb) [file 13428_2022_2048_MOESM1_ESM.pdf]

The Headphone and Loudspeaker Test–Part II: A Comprehensive Playback Device Screening Method in Internet Experiments

https://doi.org/10.3758/s13428-022-02048-3

Supplementary Material

Yves Wycisk[1], Kilian Sander[1], Benedetto Manca[2], Reinhard Kopiez[1], Friedrich Platz[3]

[1] Hanover University of Music, Drama, and Media, Institute for Musicology, Hanover, 30175, Germany
[2] Department of Mathematics and Computer Science, University of Cagliari, Cagliari, 09124, Italy
[3] University of Music and the Performing Arts Stuttgart, Institute for Musicology, Music Pedagogy and Aesthetic, Stuttgart, 70182, Germany

Table of Contents

Table S1 . . . 2
Table S2 . . . 2
Table S3 . . . 2
Table S4 . . . 3
Table S5 . . . 4
Table S6 . . . 5
Table S7 . . . 9
Table S8 . . . 9
Table S9 . . . 9
Table S10 . . . 9
Table S11 . . . 13
Figure S1 . . . 55
Figure S2 . . . 56
Figure S3 . . . 57
Figure S4 . . . 59
Section S1 Normal Approximation for a priori considerations for FWR and FAR. . . . 59
Section S2 Probabilities and a priori estimation for SCC . . . 60

Table S1

Dimensions of the Laboratory Room of the Pre-Study

Width	Length	Heigth
4.26 m	5.38 m	2.69 m

Table S2

Background Noise in the Laboratory Room of the Pre-Study

L_{Aeq}	L_{Ceq}	L_{Zeq}	L_{AFmax}	L_{ZFmax}
30.1 dB	38.1 dB	42.1 dB	42.9 dB	51.9 dB

Table S3

Reverberation Time in the Laboratory Room of the Pre-Study

Band [Hz]	RT60 (T30) [s]	Measurement Uncertainty [%]
50	0.27	38.22
63	0.78	20.06
80	0.37	25.72
100	0.51	19.71
125	0.74	14.64
160	0.63	14.03
200	0.75	11.50
250	0.79	10.04
315	0.76	9.08
400	0.70	8.44
500	0.62	8.00
630	0.58	7.35
800	0.53	6.82
1000	0.53	6.14
1250	0.57	5.27
1600	0.55	4.75
2000	0.56	4.22
2500	0.55	3.81
3150	0.53	3.45
4000	0.52	3.09
5000	0.50	2.82
6300	0.46	2.61
8000	0.43	2.41
10000	0.35	2.37

Note. An NTi Audio XL2 device was used with an NTi Audio M4260 microphone (sensitivity $33.4\,mV\,Pa^{-1}$). Measurement was conducted in "Full mode" with a resolution of 1/3 octave and from 40 to 140 dB. RT60 values are average results for three measurements. Overall average reverberation time was 0.57 seconds.

Table S4

Statistical Tests for Conditional Independence in the Pre-Study

Threshold		Headphones ($n = 80$)		Loudspeakers ($n = 80$)	
Test A	Test B	Test	p	Test	p
1	1	Multinomial	0.5319	Multinomial	0.2738
1	2	Multinomial	0.3797	Multinomial	0.7310
1	3	Multinomial	0.8456	Multinomial	0.2606
1	4	Multinomial	0.7770	Multinomial	1.0000
2	1	Multinomial	0.5319	Multinomial	0.1197
2	2	Multinomial	0.3797	Multinomial	0.5471
2	3	Multinomial	0.8456	Multinomial	0.3421
2	4	Multinomial	0.7770	Multinomial	1.0000
3	1	Multinomial	0.2566	Multinomial	0.1557
3	2	Multinomial	0.1540	Multinomial	0.7976
3	3	Multinomial	0.9824	Multinomial	0.4554
3	4	Multinomial	1.0000	Multinomial	1.0000
4	1	χ^2	0.1976	χ^2	0.2203
4	2	χ^2	0.2514	Multinomial	0.8573
4	3	χ^2	0.9813	Multinomial	0.6049
4	4	χ^2	0.9971	Multinomial	1.0000

Note. Screening tests were assumed to be conditionally independent when the p-value exceeded the α-level of .10. The file `HALT_pre-study_conditional_independence_tests.txt` at https://osf.io/43gva/ contains the observed and expected frequencies used for the statistical tests in addition to their results.

Table S5

Sensitivity and Specificity for all Combinations of Test A and Test B From the Pre-Study

	Threshold			95% CI			95% CI	
Combination	Test A	Test B	Sensitivity	Lower	Upper	Specificity	Lower	Upper
A OR B	1	1	0.9841	0.9271	0.9967	0.1320	0.07449	0.2233
A OR B	1	2	0.9817	0.9235	0.9958	0.1483	0.08664	0.2422
A OR B	1	3	0.9789	0.9192	0.9947	0.1605	0.09593	0.2561
A OR B	1	4	0.9780	0.9178	0.9944	0.1625	0.09750	0.2584
A OR B	2	1	0.9841	0.9271	0.9967	0.1828	0.11335	0.2813
A OR B	2	2	0.9817	0.9235	0.9958	0.2053	0.13130	0.3063
A OR B	2	3	0.9789	0.9192	0.9947	0.2222	0.14503	0.3248
A OR B	2	4	0.9780	0.9178	0.9944	0.2250	0.14733	0.3279
A OR B	3	1	0.9734	0.9111	0.9924	0.2336	0.15441	0.3372
A OR B	3	2	0.9695	0.9054	0.9906	0.2623	0.17844	0.3680
A OR B	3	3	0.9648	0.8987	0.9884	0.2839	0.19679	0.3908
A OR B	3	4	0.9633	0.8965	0.9876	0.2875	0.19987	0.3946
A OR B	4	1	0.9309	0.8534	0.9689	0.3453	0.25038	0.4544
A OR B	4	2	0.9208	0.8406	0.9624	0.3878	0.28853	0.4974
A OR B	4	3	0.9086	0.8254	0.9543	0.4197	0.31767	0.5291
A OR B	4	4	0.9045	0.8204	0.9516	0.4250	0.32257	0.5343
A AND B	1	1	0.5534	0.4445	0.6574	0.8430	0.74781	0.9067
A AND B	1	2	0.4933	0.3866	0.6006	0.9267	0.84806	0.9663
A AND B	1	3	0.4211	0.3190	0.5305	0.9895	0.93583	0.9984
A AND B	1	4	0.3970	0.2969	0.5066	1.0000	0.95418	1.0000
A AND B	2	1	0.5534	0.4445	0.6574	0.8547	0.76127	0.9156
A AND B	2	2	0.4933	0.3866	0.6006	0.9322	0.85504	0.9697
A AND B	2	3	0.4211	0.3190	0.5305	0.9903	0.93712	0.9986
A AND B	2	4	0.3970	0.2969	0.5066	1.0000	0.95418	1.0000
A AND B	3	1	0.5391	0.4306	0.6440	0.8664	0.77488	0.9244
A AND B	3	2	0.4805	0.3744	0.5883	0.9377	0.86210	0.9731
A AND B	3	3	0.4102	0.3089	0.5196	0.9911	0.93841	0.9988
A AND B	3	4	0.3867	0.2875	0.4963	1.0000	0.95418	1.0000
A AND B	4	1	0.4816	0.3755	0.5894	0.8922	0.80544	0.9430
A AND B	4	2	0.4292	0.3265	0.5385	0.9497	0.87796	0.9802
A AND B	4	3	0.3664	0.2692	0.4758	0.9928	0.94131	0.9992
A AND B	4	4	0.3455	0.2505	0.4546	1.0000	0.95418	1.0000

Note. The values are calculated based on the characteristics of the individual tests.

Table S6

Evaluation Keys for all Screening Tests and Their Combinations

Test A result			1				0			
Test B result			1		0		1		0	
Test C result			1	0	1	0	1	0	1	0
logical expression	(w_A, w_b, w_C)	EK	$G\,(A, B, C)$							
Test A HP	$(1, 0, 0)$	1	1	1	1	1	0	0	0	0
Test B HP	$(0, 1, 0)$	2	1	1	0	0	1	1	0	0
Test C HP	$(0, 0, 1)$	3	1	0	1	0	1	0	1	0
Test A AND Test B HP	$\left(\frac{1}{2}, \frac{1}{2}, 0\right)$	4	1	1	0	0	0	0	0	0
Test A OR Test B HP	$(1, 1, 0)$	5	1	1	1	1	1	1	0	0
Test B AND Test C HP	$\left(0, \frac{1}{2}, \frac{1}{2}\right)$	6	1	0	0	0	1	0	0	0
Test B OR Test C HP	$(0, 1, 1)$	7	1	1	1	0	1	1	1	0
Test A AND Test C HP	$\left(\frac{1}{2}, 0, \frac{1}{2}\right)$	8	1	0	1	0	0	0	0	0
Test A OR Test C HP	$(1, 0, 1)$	9	1	1	1	1	1	0	1	0
at least 1 HP / Test A OR Test B OR Test C	$(1, 1, 1)$	10	1	1	1	1	1	1	1	0
at least 2 HP / (Test A AND Test B) OR (Test B AND Test C) OR (Test A AND Test C)	$\left(\frac{1}{2}, \frac{1}{2}, \frac{1}{2}\right)$	11	1	1	1	0	1	0	0	0
all HP / Test A AND Test B AND Test C	$\left(\frac{1}{3}, \frac{1}{3}, \frac{1}{3}\right)$	12	1	0	0	0	0	0	0	0
(Test A OR Test B) AND Test C HP	$\left(\frac{1}{4}, \frac{1}{4}, \frac{3}{4}\right)$	13	1	0	1	0	1	0	0	0
(Test B OR Test C) AND Test A HP	$\left(\frac{3}{4}, \frac{1}{4}, \frac{1}{4}\right)$	14	1	1	1	0	0	0	0	0
(Test A OR Test C) AND Test B HP	$\left(\frac{1}{4}, \frac{3}{4}, \frac{1}{4}\right)$	15	1	1	0	0	1	0	0	0
(Test A AND Test B) OR Test C HP	$\left(\frac{1}{2}, \frac{1}{2}, 1\right)$	16	1	1	1	0	1	0	1	0
(Test B AND Test C) OR Test A HP	$\left(1, \frac{1}{2}, \frac{1}{2}\right)$	17	1	1	1	1	1	0	0	0
(Test A AND Test C) OR Test B HP	$\left(\frac{1}{2}, 1, \frac{1}{2}\right)$	18	1	1	1	0	1	1	0	0

Note. 1 = headphones; 0 = loudspeakers; EK = Evaluation key; HP = headphones; $G\,(A, B, C) = 1$ for $w_a \cdot A + w_B \cdot B + w_C \cdot C \geq 1$ and $G\,(A, B, C) = 0$ otherwise; w_A, w_b and w_C denote weights for test A, B, and C, repectively, for which $G\,(A, B, C)$ yiedls a classification equivalent to the logical expressions.

Table S7

Statistical Tests for Conditional Independence in the Main Study for Test A and Test B

Threshold		Headphones ($n = 80$)		Loudspeakers ($n = 131$)	
Test A	Test B	Test	p	Test	p
1	1	Multinomial	0.5606	χ^2	0.7197
1	2	Multinomial	0.0607	χ^2	0.9974
1	3	Multinomial	0.0607	χ^2	0.9924
1	4	Multinomial	0.0427	Multinomial	0.9859
1	5	Multinomial	0.0051	Multinomial	0.8844
1	6	Multinomial	0.0015	Multinomial	1.0000
2	1	Multinomial	0.1830	χ^2	0.9994
2	2	Multinomial	0.0196	χ^2	0.8342
2	3	Multinomial	0.0196	χ^2	0.9712
2	4	Multinomial	0.0254	χ^2	0.9953
2	5	Multinomial	0.0035	χ^2	0.9175
2	6	Multinomial	0.0024	Multinomial	0.7766
3	1	Multinomial	0.0421	χ^2	0.9625
3	2	Multinomial	0.0056	χ^2	0.9848
3	3	Multinomial	0.0056	χ^2	0.9997
3	4	Multinomial	0.0136	χ^2	0.9530
3	5	Multinomial	0.0021	χ^2	0.4175
3	6	Multinomial	0.0026	χ^2	0.1500
4	1	Multinomial	0.0118	χ^2	0.9996
4	2	Multinomial	0.0040	χ^2	0.9938
4	3	Multinomial	0.0040	χ^2	0.9964
4	4	Multinomial	0.0002	χ^2	0.3496
4	5	Multinomial	0.0000	χ^2	0.0589
4	6	Multinomial	0.0002	χ^2	0.0229
5	1	Multinomial	0.0213	χ^2	1.0000
5	2	Multinomial	0.0094	χ^2	0.9754
5	3	Multinomial	0.0094	χ^2	0.9053
5	4	Multinomial	0.0001	χ^2	0.0533
5	5	Multinomial	0.0000	χ^2	0.0050
5	6	Multinomial	0.0000	χ^2	0.0029
6	1	Multinomial	0.0427	χ^2	1.0000
6	2	Multinomial	0.0270	χ^2	0.9996
6	3	Multinomial	0.0270	χ^2	0.9918
6	4	Multinomial	0.0008	χ^2	0.4025
6	5	Multinomial	0.0002	Multinomial	0.1611
6	6	χ^2	0.0001	Multinomial	0.0891

Note. Screening tests were assumed to be conditionally independent when the p-value exceeded the α-level of .10. The file `HALT_main-study_conditional_independence_AB.txt` at https://osf.io/43gva/ contains the observed and expected frequencies used for the statistical tests in addition to their results.

Table S8

Statistical Tests for Conditional Independence in the Main Study for Test A and Test C

Threshold		Headphones ($n = 80$)		Loudspeakers ($n = 131$)	
Test A	Test C	Test	p	Test	p
1	1	Multinomial	0.5606	χ^2	0.7197
1	2	Multinomial	0.0607	χ^2	0.9974
1	3	Multinomial	0.0607	χ^2	0.9924
1	4	Multinomial	0.0427	χ^2	0.9859
1	5	Multinomial	0.0051	χ^2	0.8844
1	6	Multinomial	0.0015	χ^2	1.0000
2	1	Multinomial	0.1830	χ^2	0.9994
2	2	Multinomial	0.0196	χ^2	0.8342
2	3	Multinomial	0.0196	χ^2	0.9712
2	4	Multinomial	0.0254	χ^2	0.9953
2	5	Multinomial	0.0035	χ^2	0.9175
2	6	Multinomial	0.0024	χ^2	0.7766
3	1	Multinomial	0.0421	χ^2	0.9625
3	2	Multinomial	0.0056	χ^2	0.9848
3	3	Multinomial	0.0056	χ^2	0.9997
3	4	Multinomial	0.0136	χ^2	0.9530
3	5	Multinomial	0.0021	χ^2	0.4175
3	6	Multinomial	0.0026	χ^2	0.1500
4	1	Multinomial	0.0118	χ^2	0.9996
4	2	Multinomial	0.0040	χ^2	0.9938
4	3	Multinomial	0.0040	χ^2	0.9964
4	4	Multinomial	0.0002	χ^2	0.3496
4	5	Multinomial	0.0000	χ^2	0.0589
4	6	Multinomial	0.0002	χ^2	0.0229
5	1	Multinomial	0.0213	χ^2	1.0000
5	2	Multinomial	0.0094	χ^2	0.9754
5	3	Multinomial	0.0094	χ^2	0.9053
5	4	Multinomial	0.0001	χ^2	0.0533
5	5	Multinomial	0.0000	χ^2	0.0050
5	6	Multinomial	0.0000	χ^2	0.0029
6	1	Multinomial	0.0427	χ^2	1.0000
6	2	Multinomial	0.0270	χ^2	0.9996
6	3	Multinomial	0.0270	χ^2	0.9918
6	4	Multinomial	0.0008	χ^2	0.4025
6	5	Multinomial	0.0002	χ^2	0.1611
6	6	Multinomial	0.0001	χ^2	0.0891

Note. Screening tests were assumed to be conditionally independent when the p-value exceeded the α-level of .10. The file `HALT_main-study_conditional_independence_AC.txt` at https://osf.io/43gva/ contains the observed and expected frequencies used for the statistical tests in addition to their results.

Table S9

Statistical Tests for Conditional Independence in the Main Study for Test B and Test C

Threshold		Headphones ($n = 80$)		Loudspeakers ($n = 131$)	
Test B	Test C	Test	p	Test	p
1	1	Multinomial	0.5606	χ^2	0.7197
1	2	Multinomial	0.0607	χ^2	0.9974
1	3	Multinomial	0.0607	χ^2	0.9924
1	4	Multinomial	0.0427	χ^2	0.9859
1	5	Multinomial	0.0051	χ^2	0.8844
1	6	Multinomial	0.0015	χ^2	1.0000
2	1	Multinomial	0.1830	χ^2	0.9994
2	2	Multinomial	0.0196	χ^2	0.8342
2	3	Multinomial	0.0196	χ^2	0.9712
2	4	Multinomial	0.0254	χ^2	0.9953
2	5	Multinomial	0.0035	χ^2	0.9175
2	6	Multinomial	0.0024	χ^2	0.7766
3	1	Multinomial	0.0421	χ^2	0.9625
3	2	Multinomial	0.0056	χ^2	0.9848
3	3	Multinomial	0.0056	χ^2	0.9997
3	4	Multinomial	0.0136	χ^2	0.9530
3	5	Multinomial	0.0021	χ^2	0.4175
3	6	Multinomial	0.0026	χ^2	0.1500
4	1	Multinomial	0.0118	χ^2	0.9996
4	2	Multinomial	0.0040	χ^2	0.9938
4	3	Multinomial	0.0040	χ^2	0.9964
4	4	Multinomial	0.0002	χ^2	0.3496
4	5	Multinomial	0.0000	χ^2	0.0589
4	6	Multinomial	0.0002	χ^2	0.0229
5	1	Multinomial	0.0213	χ^2	1.0000
5	2	Multinomial	0.0094	χ^2	0.9754
5	3	Multinomial	0.0094	χ^2	0.9053
5	4	Multinomial	0.0001	χ^2	0.0533
5	5	Multinomial	0.0000	χ^2	0.0050
5	6	Multinomial	0.0000	χ^2	0.0029
6	1	Multinomial	0.0427	Multinomial	1.0000
6	2	Multinomial	0.0270	χ^2	0.9996
6	3	Multinomial	0.0270	χ^2	0.9918
6	4	Multinomial	0.0008	χ^2	0.4025
6	5	Multinomial	0.0002	χ^2	0.1611
6	6	Multinomial	0.0001	Multinomial	0.0891

Note. Screening tests were assumed to be conditionally independent when the p-value exceeded the α-level of .10. The file `HALT_main-study_conditional_independence_BC.txt` at https://osf.io/43gva/ contains the observed and expected frequencies used for the statistical tests in addition to their results.

Table S10

Statistical Tests for Conditional Independence in the Main Study for Test A, Test B, and Test C

Thresholds			headphones ($n = 80$)		loudspeakers ($n = 131$)	
Test A	Test B	Test C	Test	p	Test	p
1	1	1	Multinomial	0.1407	Multinomial	0.2767
1	1	2	Multinomial	0.1407	Multinomial	0.4794
1	1	3	Multinomial	0.0007	χ^2	0.0400
1	1	4	Multinomial	0.0007	χ^2	0.0371
1	1	5	Multinomial	0.0007	χ^2	0.0774
1	1	6	Multinomial	0.0108	Multinomial	0.2452
1	2	1	Multinomial	0.0356	Multinomial	0.7076
1	2	2	Multinomial	0.0356	Multinomial	0.8425
1	2	3	Multinomial	0.0002	Multinomial	0.4225
1	2	4	Multinomial	0.0002	χ^2	0.3762
1	2	5	Multinomial	0.0003	χ^2	0.5444
1	2	6	Multinomial	0.0051	Multinomial	0.4267
1	3	1	Multinomial	0.0356	Multinomial	0.9082
1	3	2	Multinomial	0.0356	Multinomial	0.9975
1	3	3	Multinomial	0.0002	Multinomial	0.8458
1	3	4	Multinomial	0.0002	Multinomial	0.7938
1	3	5	Multinomial	0.0003	Multinomial	0.8396
1	3	6	Multinomial	0.0051	Multinomial	0.7591
1	4	1	Multinomial	0.0480	Multinomial	0.6727
1	4	2	Multinomial	0.0480	Multinomial	0.9333
1	4	3	Multinomial	0.0015	Multinomial	0.6624
1	4	4	Multinomial	0.0015	Multinomial	0.4709
1	4	5	Multinomial	0.0001	Multinomial	0.4598
1	4	6	Multinomial	0.0015	Multinomial	0.2554
1	5	1	Multinomial	0.0093	Multinomial	0.5019
1	5	2	Multinomial	0.0093	Multinomial	0.7249
1	5	3	Multinomial	0.0003	Multinomial	0.4680
1	5	4	Multinomial	0.0003	Multinomial	0.3154
1	5	5	Multinomial	0.0000	Multinomial	0.3626
1	5	6	Multinomial	0.0002	Multinomial	0.1304
1	6	1	Multinomial	0.0054	Multinomial	0.3618
1	6	2	Multinomial	0.0054	Multinomial	0.7687
1	6	3	Multinomial	0.0004	Multinomial	0.5566
1	6	4	Multinomial	0.0004	Multinomial	0.4169
1	6	5	Multinomial	0.0000	Multinomial	0.1769
1	6	6	Multinomial	0.0002	Multinomial	0.0864
2	1	1	Multinomial	0.0517	χ^2	0.2329
2	1	2	Multinomial	0.0517	χ^2	0.4192
2	1	3	Multinomial	0.0004	χ^2	0.0294
2	1	4	Multinomial	0.0004	χ^2	0.0357
2	1	5	Multinomial	0.0005	χ^2	0.0663
2	1	6	Multinomial	0.0014	χ^2	0.1099
2	2	1	Multinomial	0.0141	Multinomial	0.2529
2	2	2	Multinomial	0.0141	χ^2	0.3728
2	2	3	Multinomial	0.0002	χ^2	0.0657
2	2	4	Multinomial	0.0002	χ^2	0.0632
2	2	5	Multinomial	0.0002	χ^2	0.1673
2	2	6	Multinomial	0.0004	χ^2	0.1144
2	3	1	Multinomial	0.0141	Multinomial	0.5041
2	3	2	Multinomial	0.0141	Multinomial	0.7711

Thresholds			headphones ($n = 80$)		loudspeakers ($n = 131$)	
Test A	Test B	Test C	Test	p	Test	p
2	3	3	Multinomial	0.0002	χ^2	0.3388
2	3	4	Multinomial	0.0002	χ^2	0.3690
2	3	5	Multinomial	0.0002	χ^2	0.5026
2	3	6	Multinomial	0.0004	χ^2	0.3595
2	4	1	Multinomial	0.0320	Multinomial	0.2490
2	4	2	Multinomial	0.0320	Multinomial	0.5296
2	4	3	Multinomial	0.0008	Multinomial	0.3115
2	4	4	Multinomial	0.0008	Multinomial	0.2460
2	4	5	Multinomial	0.0000	Multinomial	0.2026
2	4	6	Multinomial	0.0001	χ^2	0.0761
2	5	1	Multinomial	0.0072	Multinomial	0.2816
2	5	2	Multinomial	0.0072	Multinomial	0.6361
2	5	3	Multinomial	0.0002	Multinomial	0.3280
2	5	4	Multinomial	0.0002	Multinomial	0.3122
2	5	5	Multinomial	0.0000	Multinomial	0.3314
2	5	6	Multinomial	0.0000	Multinomial	0.0873
2	6	1	Multinomial	0.0079	Multinomial	0.1290
2	6	2	Multinomial	0.0079	Multinomial	0.5044
2	6	3	Multinomial	0.0004	Multinomial	0.2845
2	6	4	Multinomial	0.0004	Multinomial	0.2949
2	6	5	Multinomial	0.0000	Multinomial	0.0935
2	6	6	Multinomial	0.0000	Multinomial	0.0361
3	1	1	Multinomial	0.0168	χ^2	0.2676
3	1	2	Multinomial	0.0168	χ^2	0.6014
3	1	3	Multinomial	0.0001	χ^2	0.1735
3	1	4	Multinomial	0.0001	χ^2	0.1455
3	1	5	Multinomial	0.0002	χ^2	0.1491
3	1	6	Multinomial	0.0007	χ^2	0.2232
3	2	1	Multinomial	0.0055	χ^2	0.3182
3	2	2	Multinomial	0.0055	χ^2	0.5291
3	2	3	Multinomial	0.0001	χ^2	0.2095
3	2	4	Multinomial	0.0001	χ^2	0.1088
3	2	5	Multinomial	0.0001	χ^2	0.1101
3	2	6	Multinomial	0.0002	χ^2	0.1723
3	3	1	Multinomial	0.0055	Multinomial	0.5631
3	3	2	Multinomial	0.0055	χ^2	0.7702
3	3	3	Multinomial	0.0001	χ^2	0.4626
3	3	4	Multinomial	0.0001	χ^2	0.4894
3	3	5	Multinomial	0.0001	χ^2	0.2342
3	3	6	Multinomial	0.0002	χ^2	0.2698
3	4	1	Multinomial	0.0144	Multinomial	0.1621
3	4	2	Multinomial	0.0144	Multinomial	0.5718
3	4	3	Multinomial	0.0002	χ^2	0.4392
3	4	4	Multinomial	0.0002	χ^2	0.2396
3	4	5	Multinomial	0.0000	χ^2	0.2168
3	4	6	Multinomial	0.0001	Multinomial	0.1423
3	5	1	Multinomial	0.0039	Multinomial	0.0602
3	5	2	Multinomial	0.0039	Multinomial	0.3326
3	5	3	Multinomial	0.0001	Multinomial	0.2533
3	5	4	Multinomial	0.0001	Multinomial	0.1347
3	5	5	Multinomial	0.0000	χ^2	0.0651
3	5	6	Multinomial	0.0000	Multinomial	0.0642
3	6	1	Multinomial	0.0055	Multinomial	0.0436

Thresholds			headphones ($n = 80$)		loudspeakers ($n = 131$)	
Test A	Test B	Test C	Test	p	Test	p
3	6	2	Multinomial	0.0055	Multinomial	0.2432
3	6	3	Multinomial	0.0002	Multinomial	0.1823
3	6	4	Multinomial	0.0002	Multinomial	0.1085
3	6	5	Multinomial	0.0000	Multinomial	0.0303
3	6	6	Multinomial	0.0000	Multinomial	0.0222
4	1	1	Multinomial	0.0083	χ^2	0.4028
4	1	2	Multinomial	0.0083	χ^2	0.6477
4	1	3	Multinomial	0.0000	χ^2	0.3154
4	1	4	Multinomial	0.0000	χ^2	0.1706
4	1	5	Multinomial	0.0000	χ^2	0.2615
4	1	6	Multinomial	0.0000	χ^2	0.3150
4	2	1	Multinomial	0.0052	χ^2	0.4284
4	2	2	Multinomial	0.0052	χ^2	0.6296
4	2	3	Multinomial	0.0001	χ^2	0.4234
4	2	4	Multinomial	0.0001	χ^2	0.2048
4	2	5	Multinomial	0.0000	χ^2	0.3246
4	2	6	Multinomial	0.0000	χ^2	0.2968
4	3	1	Multinomial	0.0052	Multinomial	0.5805
4	3	2	Multinomial	0.0052	χ^2	0.8511
4	3	3	Multinomial	0.0001	χ^2	0.7344
4	3	4	Multinomial	0.0001	χ^2	0.5181
4	3	5	Multinomial	0.0000	χ^2	0.4424
4	3	6	Multinomial	0.0000	χ^2	0.2016
4	4	1	Multinomial	0.0009	Multinomial	0.1151
4	4	2	Multinomial	0.0009	Multinomial	0.3593
4	4	3	Multinomial	0.0000	χ^2	0.1740
4	4	4	Multinomial	0.0000	χ^2	0.0683
4	4	5	Multinomial	0.0000	χ^2	0.0736
4	4	6	Multinomial	0.0000	Multinomial	0.0416
4	5	1	Multinomial	0.0002	Multinomial	0.0242
4	5	2	Multinomial	0.0002	Multinomial	0.1112
4	5	3	Multinomial	0.0000	Multinomial	0.0769
4	5	4	Multinomial	0.0000	Multinomial	0.0351
4	5	5	Multinomial	0.0000	χ^2	0.0105
4	5	6	Multinomial	0.0000	Multinomial	0.0089
4	6	1	Multinomial	0.0008	Multinomial	0.0192
4	6	2	Multinomial	0.0008	Multinomial	0.0745
4	6	3	Multinomial	0.0000	Multinomial	0.0500
4	6	4	Multinomial	0.0000	Multinomial	0.0242
4	6	5	Multinomial	0.0000	Multinomial	0.0072
4	6	6	Multinomial	0.0000	Multinomial	0.0043
5	1	1	Multinomial	0.0135	χ^2	0.1132
5	1	2	Multinomial	0.0135	χ^2	0.4572
5	1	3	Multinomial	0.0001	χ^2	0.3639
5	1	4	Multinomial	0.0001	χ^2	0.3088
5	1	5	Multinomial	0.0000	χ^2	0.5476
5	1	6	Multinomial	0.0000	χ^2	0.5075
5	2	1	Multinomial	0.0108	Multinomial	0.1667
5	2	2	Multinomial	0.0108	χ^2	0.6609
5	2	3	Multinomial	0.0002	χ^2	0.5482
5	2	4	Multinomial	0.0002	χ^2	0.3216
5	2	5	Multinomial	0.0000	χ^2	0.5685
5	2	6	Multinomial	0.0000	χ^2	0.4225

Thresholds			headphones ($n = 80$)		loudspeakers ($n = 131$)	
Test A	Test B	Test C	Test	p	Test	p
5	3	1	Multinomial	0.0108	Multinomial	0.2890
5	3	2	Multinomial	0.0108	Multinomial	0.8973
5	3	3	Multinomial	0.0002	χ^2	0.7793
5	3	4	Multinomial	0.0002	χ^2	0.5466
5	3	5	Multinomial	0.0000	χ^2	0.4974
5	3	6	Multinomial	0.0000	χ^2	0.1288
5	4	1	Multinomial	0.0004	Multinomial	0.0161
5	4	2	Multinomial	0.0004	Multinomial	0.1329
5	4	3	Multinomial	0.0000	Multinomial	0.0859
5	4	4	Multinomial	0.0000	Multinomial	0.0359
5	4	5	Multinomial	0.0000	Multinomial	0.0400
5	4	6	Multinomial	0.0000	χ^2	0.0009
5	5	1	Multinomial	0.0001	Multinomial	0.0024
5	5	2	Multinomial	0.0001	Multinomial	0.0259
5	5	3	Multinomial	0.0000	Multinomial	0.0148
5	5	4	Multinomial	0.0000	Multinomial	0.0057
5	5	5	Multinomial	0.0000	Multinomial	0.0072
5	5	6	Multinomial	0.0000	Multinomial	0.0025
5	6	1	Multinomial	0.0001	Multinomial	0.0020
5	6	2	Multinomial	0.0001	Multinomial	0.0206
5	6	3	Multinomial	0.0000	Multinomial	0.0138
5	6	4	Multinomial	0.0000	Multinomial	0.0064
5	6	5	Multinomial	0.0000	Multinomial	0.0015
5	6	6	Multinomial	0.0000	Multinomial	0.0016
6	1	1	Multinomial	0.0244	Multinomial	0.2975
6	1	2	Multinomial	0.0244	χ^2	0.6501
6	1	3	Multinomial	0.0002	χ^2	0.4127
6	1	4	Multinomial	0.0002	χ^2	0.2953
6	1	5	Multinomial	0.0001	χ^2	0.4898
6	1	6	Multinomial	0.0000	Multinomial	0.5743
6	2	1	Multinomial	0.0258	Multinomial	0.4252
6	2	2	Multinomial	0.0258	Multinomial	0.8377
6	2	3	Multinomial	0.0006	χ^2	0.5862
6	2	4	Multinomial	0.0006	χ^2	0.4464
6	2	5	Multinomial	0.0000	χ^2	0.4987
6	2	6	Multinomial	0.0000	Multinomial	0.5307
6	3	1	Multinomial	0.0258	Multinomial	0.6956
6	3	2	Multinomial	0.0258	Multinomial	0.9798
6	3	3	Multinomial	0.0006	Multinomial	0.6553
6	3	4	Multinomial	0.0006	Multinomial	0.6130
6	3	5	Multinomial	0.0000	χ^2	0.3726
6	3	6	Multinomial	0.0000	χ^2	0.1226
6	4	1	Multinomial	0.0028	Multinomial	0.2831
6	4	2	Multinomial	0.0028	Multinomial	0.4982
6	4	3	Multinomial	0.0000	Multinomial	0.1429
6	4	4	Multinomial	0.0000	Multinomial	0.1018
6	4	5	Multinomial	0.0000	Multinomial	0.0684
6	4	6	Multinomial	0.0000	Multinomial	0.0374
6	5	1	Multinomial	0.0009	Multinomial	0.1441
6	5	2	Multinomial	0.0009	Multinomial	0.2590
6	5	3	Multinomial	0.0000	Multinomial	0.0626
6	5	4	Multinomial	0.0000	Multinomial	0.0440
6	5	5	Multinomial	0.0000	Multinomial	0.0320

Thresholds			headphones ($n = 80$)		loudspeakers ($n = 131$)	
Test A	Test B	Test C	Test	p	Test	p
6	5	6	Multinomial	0.0000	Multinomial	0.0154
6	6	1	Multinomial	0.0012	Multinomial	0.0659
6	6	2	Multinomial	0.0012	Multinomial	0.1799
6	6	3	Multinomial	0.0000	Multinomial	0.0612
6	6	4	Multinomial	0.0000	Multinomial	0.0435
6	6	5	Multinomial	0.0000	Multinomial	0.0148
6	6	6	Multinomial	0.0000	Multinomial	0.0146

Note. Screening tests were assumed to be conditionally independent when the p-value exceeded the α-level of .10 for the two- and three-way combinations. The file `HALT_main-study_conditional_independence_ABC.txt` at https://osf.io/43gva/ contains the observed and expected frequencies used for the statistical tests in addition to their results.

Table S11

Sensitivity and Specificity for the Combinations of Test A, B, and C from the Main Study

Combination	EK	Threshold			Sensitivity	95% CI		Specificity	95% CI	
		A	B	C		Lower	Upper		Lower	Upper
Test A HP	1	1	0	0	0.9250	0.8459	0.9652	0.1756	0.1199	0.2497
Test A HP	1	2	0	0	0.9000	0.8149	0.9485	0.2901	0.2192	0.3729
Test A HP	1	3	0	0	0.8750	0.7850	0.9307	0.4351	0.3533	0.5207
Test A HP	1	4	0	0	0.8250	0.7274	0.8928	0.5802	0.4945	0.6612
Test A HP	1	5	0	0	0.8000	0.6995	0.8730	0.6870	0.6032	0.7601
Test A HP	1	6	0	0	0.7625	0.6586	0.8424	0.7786	0.7002	0.8412
Test B HP	2	0	1	0	0.9375	0.8619	0.9730	0.4122	0.3316	0.4978
Test B HP	2	0	2	0	0.8875	0.7998	0.9397	0.5420	0.4567	0.6249
Test B HP	2	0	3	0	0.8875	0.7998	0.9397	0.6794	0.5953	0.7532
Test B HP	2	0	4	0	0.8125	0.7134	0.8829	0.8015	0.7251	0.8608
Test B HP	2	0	5	0	0.8000	0.6995	0.8730	0.8321	0.7588	0.8864
Test B HP	2	0	6	0	0.7250	0.6186	0.8108	0.8702	0.8020	0.9174
Test C HP	3	0	0	1	0.9875	0.9325	0.9978	0.2519	0.1854	0.3326
Test C HP	3	0	0	2	0.9875	0.9325	0.9978	0.3664	0.2888	0.4516
Test C HP	3	0	0	3	0.9500	0.8784	0.9804	0.4733	0.3898	0.5583
Test C HP	3	0	0	4	0.9500	0.8784	0.9804	0.5191	0.4342	0.6029
Test C HP	3	0	0	5	0.9250	0.8459	0.9652	0.5802	0.4945	0.6612
Test C HP	3	0	0	6	0.8375	0.7416	0.9025	0.7176	0.6351	0.7876
Test A AND Test B HP	4	1	1	0	0.8750	0.7850	0.9307	0.5344	0.4492	0.6176
Test A AND Test B HP	4	1	2	0	0.8500	0.7559	0.9121	0.6260	0.5406	0.7041
Test A AND Test B HP	4	1	3	0	0.8500	0.7559	0.9121	0.7405	0.6593	0.8079
Test A AND Test B HP	4	1	4	0	0.7875	0.6858	0.8629	0.8397	0.7673	0.8927
Test A AND Test B HP	4	1	5	0	0.7875	0.6858	0.8629	0.8702	0.8020	0.9174
Test A AND Test B HP	4	1	6	0	0.7250	0.6186	0.8108	0.8931	0.8286	0.9353
Test A AND Test B HP	4	2	1	0	0.8625	0.7703	0.9215	0.5802	0.4945	0.6612
Test A AND Test B HP	4	2	2	0	0.8375	0.7416	0.9025	0.6565	0.5718	0.7323
Test A AND Test B HP	4	2	3	0	0.8375	0.7416	0.9025	0.7634	0.6837	0.8280
Test A AND Test B HP	4	2	4	0	0.7750	0.6721	0.8527	0.8550	0.7846	0.9051
Test A AND Test B HP	4	2	5	0	0.7750	0.6721	0.8527	0.8702	0.8020	0.9174

		Threshold				95% CI			95% CI	
Combination	EK	A	B	C	Sensitivity	Lower	Upper	Specificity	Lower	Upper
Test A AND Test B HP	4	2	6	0	0.7125	0.6054	0.8001	0.8931	0.8286	0.9353
Test A AND Test B HP	4	3	1	0	0.8500	0.7559	0.9121	0.6794	0.5953	0.7532
Test A AND Test B HP	4	3	2	0	0.8250	0.7274	0.8928	0.7328	0.6512	0.8012
Test A AND Test B HP	4	3	3	0	0.8250	0.7274	0.8928	0.8168	0.7419	0.8737
Test A AND Test B HP	4	3	4	0	0.7625	0.6586	0.8424	0.8779	0.8108	0.9234
Test A AND Test B HP	4	3	5	0	0.7625	0.6586	0.8424	0.8779	0.8108	0.9234
Test A AND Test B HP	4	3	6	0	0.7000	0.5923	0.7894	0.8931	0.8286	0.9353
Test A AND Test B HP	4	4	1	0	0.8125	0.7134	0.8829	0.7557	0.6756	0.8213
Test A AND Test B HP	4	4	2	0	0.7875	0.6858	0.8629	0.8015	0.7251	0.8608
Test A AND Test B HP	4	4	3	0	0.7875	0.6858	0.8629	0.8702	0.8020	0.9174
Test A AND Test B HP	4	4	4	0	0.7500	0.6452	0.8319	0.8855	0.8197	0.9294
Test A AND Test B HP	4	4	5	0	0.7500	0.6452	0.8319	0.8855	0.8197	0.9294
Test A AND Test B HP	4	4	6	0	0.6875	0.5793	0.7785	0.9008	0.8376	0.9411
Test A AND Test B HP	4	5	1	0	0.7875	0.6858	0.8629	0.8168	0.7419	0.8737
Test A AND Test B HP	4	5	2	0	0.7625	0.6586	0.8424	0.8473	0.7759	0.8989
Test A AND Test B HP	4	5	3	0	0.7625	0.6586	0.8424	0.8855	0.8197	0.9294
Test A AND Test B HP	4	5	4	0	0.7375	0.6318	0.8214	0.8931	0.8286	0.9353
Test A AND Test B HP	4	5	5	0	0.7375	0.6318	0.8214	0.8931	0.8286	0.9353
Test A AND Test B HP	4	5	6	0	0.6875	0.5793	0.7785	0.9084	0.8467	0.9468
Test A AND Test B HP	4	6	1	0	0.7500	0.6452	0.8319	0.8702	0.8020	0.9174
Test A AND Test B HP	4	6	2	0	0.7250	0.6186	0.8108	0.9008	0.8376	0.9411
Test A AND Test B HP	4	6	3	0	0.7250	0.6186	0.8108	0.9237	0.8652	0.9580
Test A AND Test B HP	4	6	4	0	0.7000	0.5923	0.7894	0.9313	0.8746	0.9634
Test A AND Test B HP	4	6	5	0	0.7000	0.5923	0.7894	0.9313	0.8746	0.9634
Test A AND Test B HP	4	6	6	0	0.6500	0.5408	0.7455	0.9389	0.8841	0.9687
Test A OR Test B HP	5	1	1	0	0.9875	0.9325	0.9978	0.0534	0.0261	0.1062
Test A OR Test B HP	5	1	2	0	0.9625	0.8955	0.9872	0.0916	0.0532	0.1533
Test A OR Test B HP	5	1	3	0	0.9625	0.8955	0.9872	0.1145	0.0706	0.1803
Test A OR Test B HP	5	1	4	0	0.9500	0.8784	0.9804	0.1374	0.0887	0.2068
Test A OR Test B HP	5	1	5	0	0.9375	0.8619	0.9730	0.1374	0.0887	0.2068
Test A OR Test B HP	5	1	6	0	0.9250	0.8459	0.9652	0.1527	0.1011	0.2241
Test A OR Test B HP	5	2	1	0	0.9750	0.9134	0.9931	0.1221	0.0766	0.1892
Test A OR Test B HP	5	2	2	0	0.9500	0.8784	0.9804	0.1756	0.1199	0.2497
Test A OR Test B HP	5	2	3	0	0.9500	0.8784	0.9804	0.2061	0.1457	0.2833
Test A OR Test B HP	5	2	4	0	0.9375	0.8619	0.9730	0.2366	0.1720	0.3163
Test A OR Test B HP	5	2	5	0	0.9250	0.8459	0.9652	0.2519	0.1854	0.3326
Test A OR Test B HP	5	2	6	0	0.9125	0.8302	0.9570	0.2672	0.1988	0.3488
Test A OR Test B HP	5	3	1	0	0.9625	0.8955	0.9872	0.1679	0.1136	0.2412
Test A OR Test B HP	5	3	2	0	0.9375	0.8619	0.9730	0.2443	0.1787	0.3244
Test A OR Test B HP	5	3	3	0	0.9375	0.8619	0.9730	0.2977	0.2261	0.3809
Test A OR Test B HP	5	3	4	0	0.9250	0.8459	0.9652	0.3588	0.2817	0.4439
Test A OR Test B HP	5	3	5	0	0.9125	0.8302	0.9570	0.3893	0.3101	0.4748
Test A OR Test B HP	5	3	6	0	0.9000	0.8149	0.9485	0.4122	0.3316	0.4978
Test A OR Test B HP	5	4	1	0	0.9500	0.8784	0.9804	0.2366	0.1720	0.3163
Test A OR Test B HP	5	4	2	0	0.9250	0.8459	0.9652	0.3206	0.2468	0.4047
Test A OR Test B HP	5	4	3	0	0.9250	0.8459	0.9652	0.3893	0.3101	0.4748
Test A OR Test B HP	5	4	4	0	0.8875	0.7998	0.9397	0.4962	0.4119	0.5807
Test A OR Test B HP	5	4	5	0	0.8750	0.7850	0.9307	0.5267	0.4417	0.6102
Test A OR Test B HP	5	4	6	0	0.8625	0.7703	0.9215	0.5496	0.4642	0.6322
Test A OR Test B HP	5	5	1	0	0.9500	0.8784	0.9804	0.2824	0.2124	0.3649
Test A OR Test B HP	5	5	2	0	0.9250	0.8459	0.9652	0.3817	0.3030	0.4671
Test A OR Test B HP	5	5	3	0	0.9250	0.8459	0.9652	0.4809	0.3971	0.5658
Test A OR Test B HP	5	5	4	0	0.8750	0.7850	0.9307	0.5954	0.5098	0.6756

		Threshold				95% CI			95% CI	
Combination	EK	A	B	C	Sensitivity	Lower	Upper	Specificity	Lower	Upper
Test A OR Test B HP	5	5	5	0	0.8625	0.7703	0.9215	0.6260	0.5406	0.7041
Test A OR Test B HP	5	5	6	0	0.8375	0.7416	0.9025	0.6489	0.5639	0.7253
Test A OR Test B HP	5	6	1	0	0.9500	0.8784	0.9804	0.3206	0.2468	0.4047
Test A OR Test B HP	5	6	2	0	0.9250	0.8459	0.9652	0.4198	0.3388	0.5055
Test A OR Test B HP	5	6	3	0	0.9250	0.8459	0.9652	0.5344	0.4492	0.6176
Test A OR Test B HP	5	6	4	0	0.8750	0.7850	0.9307	0.6489	0.5639	0.7253
Test A OR Test B HP	5	6	5	0	0.8625	0.7703	0.9215	0.6794	0.5953	0.7532
Test A OR Test B HP	5	6	6	0	0.8375	0.7416	0.9025	0.7099	0.6271	0.7808
Test B AND Test C HP	6	0	1	1	0.9375	0.8619	0.9730	0.5267	0.4417	0.6102
Test B AND Test C HP	6	0	1	2	0.9375	0.8619	0.9730	0.5878	0.5022	0.6684
Test B AND Test C HP	6	0	1	3	0.9250	0.8459	0.9652	0.6336	0.5484	0.7112
Test B AND Test C HP	6	0	1	4	0.9250	0.8459	0.9652	0.6565	0.5718	0.7323
Test B AND Test C HP	6	0	1	5	0.9000	0.8149	0.9485	0.7023	0.6191	0.7739
Test B AND Test C HP	6	0	1	6	0.8125	0.7134	0.8829	0.7939	0.7167	0.8543
Test B AND Test C HP	6	0	2	1	0.8875	0.7998	0.9397	0.6260	0.5406	0.7041
Test B AND Test C HP	6	0	2	2	0.8875	0.7998	0.9397	0.6718	0.5875	0.7463
Test B AND Test C HP	6	0	2	3	0.8750	0.7850	0.9307	0.7099	0.6271	0.7808
Test B AND Test C HP	6	0	2	4	0.8750	0.7850	0.9307	0.7252	0.6432	0.7944
Test B AND Test C HP	6	0	2	5	0.8625	0.7703	0.9215	0.7634	0.6837	0.8280
Test B AND Test C HP	6	0	2	6	0.7750	0.6721	0.8527	0.8321	0.7588	0.8864
Test B AND Test C HP	6	0	3	1	0.8875	0.7998	0.9397	0.7481	0.6674	0.8146
Test B AND Test C HP	6	0	3	2	0.8875	0.7998	0.9397	0.7863	0.7084	0.8478
Test B AND Test C HP	6	0	3	3	0.8750	0.7850	0.9307	0.8092	0.7334	0.8673
Test B AND Test C HP	6	0	3	4	0.8750	0.7850	0.9307	0.8168	0.7419	0.8737
Test B AND Test C HP	6	0	3	5	0.8625	0.7703	0.9215	0.8473	0.7759	0.8989
Test B AND Test C HP	6	0	3	6	0.7750	0.6721	0.8527	0.8931	0.8286	0.9353
Test B AND Test C HP	6	0	4	1	0.8125	0.7134	0.8829	0.8321	0.7588	0.8864
Test B AND Test C HP	6	0	4	2	0.8125	0.7134	0.8829	0.8550	0.7846	0.9051
Test B AND Test C HP	6	0	4	3	0.8125	0.7134	0.8829	0.8626	0.7932	0.9113
Test B AND Test C HP	6	0	4	4	0.8125	0.7134	0.8829	0.8626	0.7932	0.9113
Test B AND Test C HP	6	0	4	5	0.8125	0.7134	0.8829	0.8779	0.8108	0.9234
Test B AND Test C HP	6	0	4	6	0.7375	0.6318	0.8214	0.9084	0.8467	0.9468
Test B AND Test C HP	6	0	5	1	0.8000	0.6995	0.8730	0.8550	0.7846	0.9051
Test B AND Test C HP	6	0	5	2	0.8000	0.6995	0.8730	0.8702	0.8020	0.9174
Test B AND Test C HP	6	0	5	3	0.8000	0.6995	0.8730	0.8779	0.8108	0.9234
Test B AND Test C HP	6	0	5	4	0.8000	0.6995	0.8730	0.8779	0.8108	0.9234
Test B AND Test C HP	6	0	5	5	0.8000	0.6995	0.8730	0.8931	0.8286	0.9353
Test B AND Test C HP	6	0	5	6	0.7375	0.6318	0.8214	0.9160	0.8559	0.9525
Test B AND Test C HP	6	0	6	1	0.7250	0.6186	0.8108	0.8779	0.8108	0.9234
Test B AND Test C HP	6	0	6	2	0.7250	0.6186	0.8108	0.8931	0.8286	0.9353
Test B AND Test C HP	6	0	6	3	0.7250	0.6186	0.8108	0.9008	0.8376	0.9411
Test B AND Test C HP	6	0	6	4	0.7250	0.6186	0.8108	0.9008	0.8376	0.9411
Test B AND Test C HP	6	0	6	5	0.7250	0.6186	0.8108	0.9008	0.8376	0.9411
Test B AND Test C HP	6	0	6	6	0.6750	0.5664	0.7676	0.9237	0.8652	0.9580
Test B OR Test C HP	7	0	1	1	0.9875	0.9325	0.9978	0.1374	0.0887	0.2068
Test B OR Test C HP	7	0	1	2	0.9875	0.9325	0.9978	0.1908	0.1327	0.2666
Test B OR Test C HP	7	0	1	3	0.9625	0.8955	0.9872	0.2519	0.1854	0.3326
Test B OR Test C HP	7	0	1	4	0.9625	0.8955	0.9872	0.2748	0.2056	0.3568
Test B OR Test C HP	7	0	1	5	0.9625	0.8955	0.9872	0.2901	0.2192	0.3729
Test B OR Test C HP	7	0	1	6	0.9625	0.8955	0.9872	0.3359	0.2607	0.4204
Test B OR Test C HP	7	0	2	1	0.9875	0.9325	0.9978	0.1679	0.1136	0.2412
Test B OR Test C HP	7	0	2	2	0.9875	0.9325	0.9978	0.2366	0.1720	0.3163
Test B OR Test C HP	7	0	2	3	0.9625	0.8955	0.9872	0.3053	0.2330	0.3888

		Threshold				95% CI			95% CI	
Combination	EK	A	B	C	Sensitivity	Lower	Upper	Specificity	Lower	Upper
Test B OR Test C HP	7	0	2	4	0.9625	0.8955	0.9872	0.3359	0.2607	0.4204
Test B OR Test C HP	7	0	2	5	0.9500	0.8784	0.9804	0.3588	0.2817	0.4439
Test B OR Test C HP	7	0	2	6	0.9500	0.8784	0.9804	0.4275	0.3460	0.5131
Test B OR Test C HP	7	0	3	1	0.9875	0.9325	0.9978	0.1832	0.1263	0.2581
Test B OR Test C HP	7	0	3	2	0.9875	0.9325	0.9978	0.2595	0.1921	0.3407
Test B OR Test C HP	7	0	3	3	0.9625	0.8955	0.9872	0.3435	0.2677	0.4282
Test B OR Test C HP	7	0	3	4	0.9625	0.8955	0.9872	0.3817	0.3030	0.4671
Test B OR Test C HP	7	0	3	5	0.9500	0.8784	0.9804	0.4122	0.3316	0.4978
Test B OR Test C HP	7	0	3	6	0.9500	0.8784	0.9804	0.5038	0.4193	0.5881
Test B OR Test C HP	7	0	4	1	0.9875	0.9325	0.9978	0.2214	0.1588	0.2998
Test B OR Test C HP	7	0	4	2	0.9875	0.9325	0.9978	0.3130	0.2399	0.3968
Test B OR Test C HP	7	0	4	3	0.9500	0.8784	0.9804	0.4122	0.3316	0.4978
Test B OR Test C HP	7	0	4	4	0.9500	0.8784	0.9804	0.4580	0.3751	0.5433
Test B OR Test C HP	7	0	4	5	0.9250	0.8459	0.9652	0.5038	0.4193	0.5881
Test B OR Test C HP	7	0	4	6	0.9125	0.8302	0.9570	0.6107	0.5252	0.6899
Test B OR Test C HP	7	0	5	1	0.9875	0.9325	0.9978	0.2290	0.1654	0.3081
Test B OR Test C HP	7	0	5	2	0.9875	0.9325	0.9978	0.3282	0.2537	0.4125
Test B OR Test C HP	7	0	5	3	0.9500	0.8784	0.9804	0.4275	0.3460	0.5131
Test B OR Test C HP	7	0	5	4	0.9500	0.8784	0.9804	0.4733	0.3898	0.5583
Test B OR Test C HP	7	0	5	5	0.9250	0.8459	0.9652	0.5191	0.4342	0.6029
Test B OR Test C HP	7	0	5	6	0.9000	0.8149	0.9485	0.6336	0.5484	0.7112
Test B OR Test C HP	7	0	6	1	0.9875	0.9325	0.9978	0.2443	0.1787	0.3244
Test B OR Test C HP	7	0	6	2	0.9875	0.9325	0.9978	0.3435	0.2677	0.4282
Test B OR Test C HP	7	0	6	3	0.9500	0.8784	0.9804	0.4427	0.3605	0.5282
Test B OR Test C HP	7	0	6	4	0.9500	0.8784	0.9804	0.4885	0.4045	0.5732
Test B OR Test C HP	7	0	6	5	0.9250	0.8459	0.9652	0.5496	0.4642	0.6322
Test B OR Test C HP	7	0	6	6	0.8875	0.7998	0.9397	0.6641	0.5796	0.7393
Test A AND Test C HP	8	1	0	1	0.9125	0.8302	0.9570	0.3664	0.2888	0.4516
Test A AND Test C HP	8	1	0	2	0.9125	0.8302	0.9570	0.4809	0.3971	0.5658
Test A AND Test C HP	8	1	0	3	0.8875	0.7998	0.9397	0.5496	0.4642	0.6322
Test A AND Test C HP	8	1	0	4	0.8875	0.7998	0.9397	0.5954	0.5098	0.6756
Test A AND Test C HP	8	1	0	5	0.8750	0.7850	0.9307	0.6489	0.5639	0.7253
Test A AND Test C HP	8	1	0	6	0.8000	0.6995	0.8730	0.7405	0.6593	0.8079
Test A AND Test C HP	8	2	0	1	0.8875	0.7998	0.9397	0.4275	0.3460	0.5131
Test A AND Test C HP	8	2	0	2	0.8875	0.7998	0.9397	0.5191	0.4342	0.6029
Test A AND Test C HP	8	2	0	3	0.8750	0.7850	0.9307	0.5878	0.5022	0.6684
Test A AND Test C HP	8	2	0	4	0.8750	0.7850	0.9307	0.6336	0.5484	0.7112
Test A AND Test C HP	8	2	0	5	0.8625	0.7703	0.9215	0.6718	0.5875	0.7463
Test A AND Test C HP	8	2	0	6	0.8000	0.6995	0.8730	0.7557	0.6756	0.8213
Test A AND Test C HP	8	3	0	1	0.8750	0.7850	0.9307	0.5344	0.4492	0.6176
Test A AND Test C HP	8	3	0	2	0.8750	0.7850	0.9307	0.6183	0.5329	0.6970
Test A AND Test C HP	8	3	0	3	0.8625	0.7703	0.9215	0.6794	0.5953	0.7532
Test A AND Test C HP	8	3	0	4	0.8625	0.7703	0.9215	0.7099	0.6271	0.7808
Test A AND Test C HP	8	3	0	5	0.8500	0.7559	0.9121	0.7328	0.6512	0.8012
Test A AND Test C HP	8	3	0	6	0.7875	0.6858	0.8629	0.8015	0.7251	0.8608
Test A AND Test C HP	8	4	0	1	0.8250	0.7274	0.8928	0.6489	0.5639	0.7253
Test A AND Test C HP	8	4	0	2	0.8250	0.7274	0.8928	0.7099	0.6271	0.7808
Test A AND Test C HP	8	4	0	3	0.8250	0.7274	0.8928	0.7557	0.6756	0.8213
Test A AND Test C HP	8	4	0	4	0.8250	0.7274	0.8928	0.7710	0.6919	0.8346
Test A AND Test C HP	8	4	0	5	0.8250	0.7274	0.8928	0.7939	0.7167	0.8543
Test A AND Test C HP	8	4	0	6	0.7750	0.6721	0.8527	0.8473	0.7759	0.8989
Test A AND Test C HP	8	5	0	1	0.8000	0.6995	0.8730	0.7176	0.6351	0.7876
Test A AND Test C HP	8	5	0	2	0.8000	0.6995	0.8730	0.7786	0.7002	0.8412

		Threshold				95% CI			95% CI	
Combination	EK	A	B	C	Sensitivity	Lower	Upper	Specificity	Lower	Upper
Test A AND Test C HP	8	5	0	3	0.8000	0.6995	0.8730	0.8244	0.7503	0.8801
Test A AND Test C HP	8	5	0	4	0.8000	0.6995	0.8730	0.8397	0.7673	0.8927
Test A AND Test C HP	8	5	0	5	0.8000	0.6995	0.8730	0.8626	0.7932	0.9113
Test A AND Test C HP	8	5	0	6	0.7625	0.6586	0.8424	0.8931	0.8286	0.9353
Test A AND Test C HP	8	6	0	1	0.7625	0.6586	0.8424	0.8015	0.7251	0.8608
Test A AND Test C HP	8	6	0	2	0.7625	0.6586	0.8424	0.8550	0.7846	0.9051
Test A AND Test C HP	8	6	0	3	0.7625	0.6586	0.8424	0.8855	0.8197	0.9294
Test A AND Test C HP	8	6	0	4	0.7625	0.6586	0.8424	0.8855	0.8197	0.9294
Test A AND Test C HP	8	6	0	5	0.7625	0.6586	0.8424	0.8931	0.8286	0.9353
Test A AND Test C HP	8	6	0	6	0.7375	0.6318	0.8214	0.9237	0.8652	0.9580
Test A OR Test C HP	9	1	0	1	1.0000	0.9542	1.0000	0.0611	0.0313	0.1159
Test A OR Test C HP	9	1	0	2	1.0000	0.9542	1.0000	0.0611	0.0313	0.1159
Test A OR Test C HP	9	1	0	3	0.9875	0.9325	0.9978	0.0992	0.0589	0.1624
Test A OR Test C HP	9	1	0	4	0.9875	0.9325	0.9978	0.0992	0.0589	0.1624
Test A OR Test C HP	9	1	0	5	0.9750	0.9134	0.9931	0.1069	0.0647	0.1714
Test A OR Test C HP	9	1	0	6	0.9625	0.8955	0.9872	0.1527	0.1011	0.2241
Test A OR Test C HP	9	2	0	1	1.0000	0.9542	1.0000	0.1145	0.0706	0.1803
Test A OR Test C HP	9	2	0	2	1.0000	0.9542	1.0000	0.1374	0.0887	0.2068
Test A OR Test C HP	9	2	0	3	0.9750	0.9134	0.9931	0.1756	0.1199	0.2497
Test A OR Test C HP	9	2	0	4	0.9750	0.9134	0.9931	0.1756	0.1199	0.2497
Test A OR Test C HP	9	2	0	5	0.9625	0.8955	0.9872	0.1985	0.1392	0.2749
Test A OR Test C HP	9	2	0	6	0.9375	0.8619	0.9730	0.2519	0.1854	0.3326
Test A OR Test C HP	9	3	0	1	0.9875	0.9325	0.9978	0.1527	0.1011	0.2241
Test A OR Test C HP	9	3	0	2	0.9875	0.9325	0.9978	0.1832	0.1263	0.2581
Test A OR Test C HP	9	3	0	3	0.9625	0.8955	0.9872	0.2290	0.1654	0.3081
Test A OR Test C HP	9	3	0	4	0.9625	0.8955	0.9872	0.2443	0.1787	0.3244
Test A OR Test C HP	9	3	0	5	0.9500	0.8784	0.9804	0.2824	0.2124	0.3649
Test A OR Test C HP	9	3	0	6	0.9250	0.8459	0.9652	0.3511	0.2747	0.4361
Test A OR Test C HP	9	4	0	1	0.9875	0.9325	0.9978	0.1832	0.1263	0.2581
Test A OR Test C HP	9	4	0	2	0.9875	0.9325	0.9978	0.2366	0.1720	0.3163
Test A OR Test C HP	9	4	0	3	0.9500	0.8784	0.9804	0.2977	0.2261	0.3809
Test A OR Test C HP	9	4	0	4	0.9500	0.8784	0.9804	0.3282	0.2537	0.4125
Test A OR Test C HP	9	4	0	5	0.9250	0.8459	0.9652	0.3664	0.2888	0.4516
Test A OR Test C HP	9	4	0	6	0.8875	0.7998	0.9397	0.4504	0.3678	0.5358
Test A OR Test C HP	9	5	0	1	0.9875	0.9325	0.9978	0.2214	0.1588	0.2998
Test A OR Test C HP	9	5	0	2	0.9875	0.9325	0.9978	0.2748	0.2056	0.3568
Test A OR Test C HP	9	5	0	3	0.9500	0.8784	0.9804	0.3359	0.2607	0.4204
Test A OR Test C HP	9	5	0	4	0.9500	0.8784	0.9804	0.3664	0.2888	0.4516
Test A OR Test C HP	9	5	0	5	0.9250	0.8459	0.9652	0.4046	0.3244	0.4902
Test A OR Test C HP	9	5	0	6	0.8750	0.7850	0.9307	0.5115	0.4268	0.5955
Test A OR Test C HP	9	6	0	1	0.9875	0.9325	0.9978	0.2290	0.1654	0.3081
Test A OR Test C HP	9	6	0	2	0.9875	0.9325	0.9978	0.2901	0.2192	0.3729
Test A OR Test C HP	9	6	0	3	0.9500	0.8784	0.9804	0.3664	0.2888	0.4516
Test A OR Test C HP	9	6	0	4	0.9500	0.8784	0.9804	0.4122	0.3316	0.4978
Test A OR Test C HP	9	6	0	5	0.9250	0.8459	0.9652	0.4656	0.3824	0.5508
Test A OR Test C HP	9	6	0	6	0.8625	0.7703	0.9215	0.5725	0.4869	0.6540
at least 1 HP	10	1	1	1	1.0000	0.9542	1.0000	0.0153	0.0042	0.0540
at least 1 HP	10	1	1	2	1.0000	0.9542	1.0000	0.0153	0.0042	0.0540
at least 1 HP	10	1	1	3	1.0000	0.9542	1.0000	0.0229	0.0078	0.0652
at least 1 HP	10	1	1	4	1.0000	0.9542	1.0000	0.0229	0.0078	0.0652
at least 1 HP	10	1	1	5	1.0000	0.9542	1.0000	0.0229	0.0078	0.0652
at least 1 HP	10	1	1	6	1.0000	0.9542	1.0000	0.0534	0.0261	0.1062
at least 1 HP	10	1	2	1	1.0000	0.9542	1.0000	0.0382	0.0164	0.0862

		Threshold				95% CI			95% CI	
Combination	EK	A	B	C	Sensitivity	Lower	Upper	Specificity	Lower	Upper
at least 1 HP	10	1	2	2	1.0000	0.9542	1.0000	0.0382	0.0164	0.0862
at least 1 HP	10	1	2	3	1.0000	0.9542	1.0000	0.0534	0.0261	0.1062
at least 1 HP	10	1	2	4	1.0000	0.9542	1.0000	0.0534	0.0261	0.1062
at least 1 HP	10	1	2	5	0.9875	0.9325	0.9978	0.0534	0.0261	0.1062
at least 1 HP	10	1	2	6	0.9875	0.9325	0.9978	0.0840	0.0475	0.1441
at least 1 HP	10	1	3	1	1.0000	0.9542	1.0000	0.0382	0.0164	0.0862
at least 1 HP	10	1	3	2	1.0000	0.9542	1.0000	0.0382	0.0164	0.0862
at least 1 HP	10	1	3	3	1.0000	0.9542	1.0000	0.0611	0.0313	0.1159
at least 1 HP	10	1	3	4	1.0000	0.9542	1.0000	0.0611	0.0313	0.1159
at least 1 HP	10	1	3	5	0.9875	0.9325	0.9978	0.0611	0.0313	0.1159
at least 1 HP	10	1	3	6	0.9875	0.9325	0.9978	0.0992	0.0589	0.1624
at least 1 HP	10	1	4	1	1.0000	0.9542	1.0000	0.0458	0.0212	0.0963
at least 1 HP	10	1	4	2	1.0000	0.9542	1.0000	0.0458	0.0212	0.0963
at least 1 HP	10	1	4	3	0.9875	0.9325	0.9978	0.0840	0.0475	0.1441
at least 1 HP	10	1	4	4	0.9875	0.9325	0.9978	0.0840	0.0475	0.1441
at least 1 HP	10	1	4	5	0.9750	0.9134	0.9931	0.0840	0.0475	0.1441
at least 1 HP	10	1	4	6	0.9750	0.9134	0.9931	0.1221	0.0766	0.1892
at least 1 HP	10	1	5	1	1.0000	0.9542	1.0000	0.0458	0.0212	0.0963
at least 1 HP	10	1	5	2	1.0000	0.9542	1.0000	0.0458	0.0212	0.0963
at least 1 HP	10	1	5	3	0.9875	0.9325	0.9978	0.0840	0.0475	0.1441
at least 1 HP	10	1	5	4	0.9875	0.9325	0.9978	0.0840	0.0475	0.1441
at least 1 HP	10	1	5	5	0.9750	0.9134	0.9931	0.0840	0.0475	0.1441
at least 1 HP	10	1	5	6	0.9625	0.8955	0.9872	0.1221	0.0766	0.1892
at least 1 HP	10	1	6	1	1.0000	0.9542	1.0000	0.0534	0.0261	0.1062
at least 1 HP	10	1	6	2	1.0000	0.9542	1.0000	0.0534	0.0261	0.1062
at least 1 HP	10	1	6	3	0.9875	0.9325	0.9978	0.0916	0.0532	0.1533
at least 1 HP	10	1	6	4	0.9875	0.9325	0.9978	0.0916	0.0532	0.1533
at least 1 HP	10	1	6	5	0.9750	0.9134	0.9931	0.0992	0.0589	0.1624
at least 1 HP	10	1	6	6	0.9625	0.8955	0.9872	0.1374	0.0887	0.2068
at least 1 HP	10	2	1	1	1.0000	0.9542	1.0000	0.0534	0.0261	0.1062
at least 1 HP	10	2	1	2	1.0000	0.9542	1.0000	0.0611	0.0313	0.1159
at least 1 HP	10	2	1	3	0.9875	0.9325	0.9978	0.0687	0.0366	0.1254
at least 1 HP	10	2	1	4	0.9875	0.9325	0.9978	0.0687	0.0366	0.1254
at least 1 HP	10	2	1	5	0.9875	0.9325	0.9978	0.0763	0.0420	0.1348
at least 1 HP	10	2	1	6	0.9875	0.9325	0.9978	0.1069	0.0647	0.1714
at least 1 HP	10	2	2	1	1.0000	0.9542	1.0000	0.0763	0.0420	0.1348
at least 1 HP	10	2	2	2	1.0000	0.9542	1.0000	0.0840	0.0475	0.1441
at least 1 HP	10	2	2	3	0.9875	0.9325	0.9978	0.0992	0.0589	0.1624
at least 1 HP	10	2	2	4	0.9875	0.9325	0.9978	0.0992	0.0589	0.1624
at least 1 HP	10	2	2	5	0.9750	0.9134	0.9931	0.1145	0.0706	0.1803
at least 1 HP	10	2	2	6	0.9750	0.9134	0.9931	0.1527	0.1011	0.2241
at least 1 HP	10	2	3	1	1.0000	0.9542	1.0000	0.0840	0.0475	0.1441
at least 1 HP	10	2	3	2	1.0000	0.9542	1.0000	0.0916	0.0532	0.1533
at least 1 HP	10	2	3	3	0.9875	0.9325	0.9978	0.1145	0.0706	0.1803
at least 1 HP	10	2	3	4	0.9875	0.9325	0.9978	0.1145	0.0706	0.1803
at least 1 HP	10	2	3	5	0.9750	0.9134	0.9931	0.1298	0.0826	0.1980
at least 1 HP	10	2	3	6	0.9750	0.9134	0.9931	0.1756	0.1199	0.2497
at least 1 HP	10	2	4	1	1.0000	0.9542	1.0000	0.0916	0.0532	0.1533
at least 1 HP	10	2	4	2	1.0000	0.9542	1.0000	0.1069	0.0647	0.1714
at least 1 HP	10	2	4	3	0.9750	0.9134	0.9931	0.1450	0.0949	0.2154
at least 1 HP	10	2	4	4	0.9750	0.9134	0.9931	0.1450	0.0949	0.2154
at least 1 HP	10	2	4	5	0.9625	0.8955	0.9872	0.1603	0.1073	0.2327
at least 1 HP	10	2	4	6	0.9625	0.8955	0.9872	0.2061	0.1457	0.2833

		Threshold				95% CI			95% CI	
Combination	EK	A	B	C	Sensitivity	Lower	Upper	Specificity	Lower	Upper
at least 1 HP	10	2	5	1	1.0000	0.9542	1.0000	0.0992	0.0589	0.1624
at least 1 HP	10	2	5	2	1.0000	0.9542	1.0000	0.1221	0.0766	0.1892
at least 1 HP	10	2	5	3	0.9750	0.9134	0.9931	0.1603	0.1073	0.2327
at least 1 HP	10	2	5	4	0.9750	0.9134	0.9931	0.1603	0.1073	0.2327
at least 1 HP	10	2	5	5	0.9625	0.8955	0.9872	0.1756	0.1199	0.2497
at least 1 HP	10	2	5	6	0.9500	0.8784	0.9804	0.2214	0.1588	0.2998
at least 1 HP	10	2	6	1	1.0000	0.9542	1.0000	0.1069	0.0647	0.1714
at least 1 HP	10	2	6	2	1.0000	0.9542	1.0000	0.1298	0.0826	0.1980
at least 1 HP	10	2	6	3	0.9750	0.9134	0.9931	0.1679	0.1136	0.2412
at least 1 HP	10	2	6	4	0.9750	0.9134	0.9931	0.1679	0.1136	0.2412
at least 1 HP	10	2	6	5	0.9625	0.8955	0.9872	0.1908	0.1327	0.2666
at least 1 HP	10	2	6	6	0.9500	0.8784	0.9804	0.2366	0.1720	0.3163
at least 1 HP	10	3	1	1	0.9875	0.9325	0.9978	0.0763	0.0420	0.1348
at least 1 HP	10	3	1	2	0.9875	0.9325	0.9978	0.0840	0.0475	0.1441
at least 1 HP	10	3	1	3	0.9750	0.9134	0.9931	0.0992	0.0589	0.1624
at least 1 HP	10	3	1	4	0.9750	0.9134	0.9931	0.1069	0.0647	0.1714
at least 1 HP	10	3	1	5	0.9750	0.9134	0.9931	0.1145	0.0706	0.1803
at least 1 HP	10	3	1	6	0.9750	0.9134	0.9931	0.1450	0.0949	0.2154
at least 1 HP	10	3	2	1	0.9875	0.9325	0.9978	0.0992	0.0589	0.1624
at least 1 HP	10	3	2	2	0.9875	0.9325	0.9978	0.1069	0.0647	0.1714
at least 1 HP	10	3	2	3	0.9750	0.9134	0.9931	0.1298	0.0826	0.1980
at least 1 HP	10	3	2	4	0.9750	0.9134	0.9931	0.1374	0.0887	0.2068
at least 1 HP	10	3	2	5	0.9625	0.8955	0.9872	0.1527	0.1011	0.2241
at least 1 HP	10	3	2	6	0.9625	0.8955	0.9872	0.1985	0.1392	0.2749
at least 1 HP	10	3	3	1	0.9875	0.9325	0.9978	0.1069	0.0647	0.1714
at least 1 HP	10	3	3	2	0.9875	0.9325	0.9978	0.1145	0.0706	0.1803
at least 1 HP	10	3	3	3	0.9750	0.9134	0.9931	0.1450	0.0949	0.2154
at least 1 HP	10	3	3	4	0.9750	0.9134	0.9931	0.1603	0.1073	0.2327
at least 1 HP	10	3	3	5	0.9625	0.8955	0.9872	0.1756	0.1199	0.2497
at least 1 HP	10	3	3	6	0.9625	0.8955	0.9872	0.2290	0.1654	0.3081
at least 1 HP	10	3	4	1	0.9875	0.9325	0.9978	0.1221	0.0766	0.1892
at least 1 HP	10	3	4	2	0.9875	0.9325	0.9978	0.1450	0.0949	0.2154
at least 1 HP	10	3	4	3	0.9625	0.8955	0.9872	0.1908	0.1327	0.2666
at least 1 HP	10	3	4	4	0.9625	0.8955	0.9872	0.2061	0.1457	0.2833
at least 1 HP	10	3	4	5	0.9500	0.8784	0.9804	0.2366	0.1720	0.3163
at least 1 HP	10	3	4	6	0.9500	0.8784	0.9804	0.2901	0.2192	0.3729
at least 1 HP	10	3	5	1	0.9875	0.9325	0.9978	0.1298	0.0826	0.1980
at least 1 HP	10	3	5	2	0.9875	0.9325	0.9978	0.1603	0.1073	0.2327
at least 1 HP	10	3	5	3	0.9625	0.8955	0.9872	0.2061	0.1457	0.2833
at least 1 HP	10	3	5	4	0.9625	0.8955	0.9872	0.2214	0.1588	0.2998
at least 1 HP	10	3	5	5	0.9500	0.8784	0.9804	0.2519	0.1854	0.3326
at least 1 HP	10	3	5	6	0.9375	0.8619	0.9730	0.3130	0.2399	0.3968
at least 1 HP	10	3	6	1	0.9875	0.9325	0.9978	0.1450	0.0949	0.2154
at least 1 HP	10	3	6	2	0.9875	0.9325	0.9978	0.1756	0.1199	0.2497
at least 1 HP	10	3	6	3	0.9625	0.8955	0.9872	0.2214	0.1588	0.2998
at least 1 HP	10	3	6	4	0.9625	0.8955	0.9872	0.2366	0.1720	0.3163
at least 1 HP	10	3	6	5	0.9500	0.8784	0.9804	0.2748	0.2056	0.3568
at least 1 HP	10	3	6	6	0.9375	0.8619	0.9730	0.3359	0.2607	0.4204
at least 1 HP	10	4	1	1	0.9875	0.9325	0.9978	0.0916	0.0532	0.1533
at least 1 HP	10	4	1	2	0.9875	0.9325	0.9978	0.1221	0.0766	0.1892
at least 1 HP	10	4	1	3	0.9625	0.8955	0.9872	0.1527	0.1011	0.2241
at least 1 HP	10	4	1	4	0.9625	0.8955	0.9872	0.1603	0.1073	0.2327
at least 1 HP	10	4	1	5	0.9625	0.8955	0.9872	0.1679	0.1136	0.2412

		Threshold				95% CI			95% CI	
Combination	EK	A	B	C	Sensitivity	Lower	Upper	Specificity	Lower	Upper
at least 1 HP	10	4	1	6	0.9625	0.8955	0.9872	0.1985	0.1392	0.2749
at least 1 HP	10	4	2	1	0.9875	0.9325	0.9978	0.1145	0.0706	0.1803
at least 1 HP	10	4	2	2	0.9875	0.9325	0.9978	0.1450	0.0949	0.2154
at least 1 HP	10	4	2	3	0.9625	0.8955	0.9872	0.1832	0.1263	0.2581
at least 1 HP	10	4	2	4	0.9625	0.8955	0.9872	0.1985	0.1392	0.2749
at least 1 HP	10	4	2	5	0.9500	0.8784	0.9804	0.2137	0.1522	0.2916
at least 1 HP	10	4	2	6	0.9500	0.8784	0.9804	0.2595	0.1921	0.3407
at least 1 HP	10	4	3	1	0.9875	0.9325	0.9978	0.1221	0.0766	0.1892
at least 1 HP	10	4	3	2	0.9875	0.9325	0.9978	0.1527	0.1011	0.2241
at least 1 HP	10	4	3	3	0.9625	0.8955	0.9872	0.1985	0.1392	0.2749
at least 1 HP	10	4	3	4	0.9625	0.8955	0.9872	0.2214	0.1588	0.2998
at least 1 HP	10	4	3	5	0.9500	0.8784	0.9804	0.2366	0.1720	0.3163
at least 1 HP	10	4	3	6	0.9500	0.8784	0.9804	0.2901	0.2192	0.3729
at least 1 HP	10	4	4	1	0.9875	0.9325	0.9978	0.1527	0.1011	0.2241
at least 1 HP	10	4	4	2	0.9875	0.9325	0.9978	0.1985	0.1392	0.2749
at least 1 HP	10	4	4	3	0.9500	0.8784	0.9804	0.2595	0.1921	0.3407
at least 1 HP	10	4	4	4	0.9500	0.8784	0.9804	0.2901	0.2192	0.3729
at least 1 HP	10	4	4	5	0.9250	0.8459	0.9652	0.3206	0.2468	0.4047
at least 1 HP	10	4	4	6	0.9125	0.8302	0.9570	0.3817	0.3030	0.4671
at least 1 HP	10	4	5	1	0.9875	0.9325	0.9978	0.1603	0.1073	0.2327
at least 1 HP	10	4	5	2	0.9875	0.9325	0.9978	0.2137	0.1522	0.2916
at least 1 HP	10	4	5	3	0.9500	0.8784	0.9804	0.2748	0.2056	0.3568
at least 1 HP	10	4	5	4	0.9500	0.8784	0.9804	0.3053	0.2330	0.3888
at least 1 HP	10	4	5	5	0.9250	0.8459	0.9652	0.3359	0.2607	0.4204
at least 1 HP	10	4	5	6	0.9000	0.8149	0.9485	0.4046	0.3244	0.4902
at least 1 HP	10	4	6	1	0.9875	0.9325	0.9978	0.1756	0.1199	0.2497
at least 1 HP	10	4	6	2	0.9875	0.9325	0.9978	0.2290	0.1654	0.3081
at least 1 HP	10	4	6	3	0.9500	0.8784	0.9804	0.2901	0.2192	0.3729
at least 1 HP	10	4	6	4	0.9500	0.8784	0.9804	0.3206	0.2468	0.4047
at least 1 HP	10	4	6	5	0.9250	0.8459	0.9652	0.3588	0.2817	0.4439
at least 1 HP	10	4	6	6	0.9000	0.8149	0.9485	0.4275	0.3460	0.5131
at least 1 HP	10	5	1	1	0.9875	0.9325	0.9978	0.1221	0.0766	0.1892
at least 1 HP	10	5	1	2	0.9875	0.9325	0.9978	0.1527	0.1011	0.2241
at least 1 HP	10	5	1	3	0.9625	0.8955	0.9872	0.1832	0.1263	0.2581
at least 1 HP	10	5	1	4	0.9625	0.8955	0.9872	0.1908	0.1327	0.2666
at least 1 HP	10	5	1	5	0.9625	0.8955	0.9872	0.1985	0.1392	0.2749
at least 1 HP	10	5	1	6	0.9625	0.8955	0.9872	0.2290	0.1654	0.3081
at least 1 HP	10	5	2	1	0.9875	0.9325	0.9978	0.1450	0.0949	0.2154
at least 1 HP	10	5	2	2	0.9875	0.9325	0.9978	0.1756	0.1199	0.2497
at least 1 HP	10	5	2	3	0.9625	0.8955	0.9872	0.2137	0.1522	0.2916
at least 1 HP	10	5	2	4	0.9625	0.8955	0.9872	0.2290	0.1654	0.3081
at least 1 HP	10	5	2	5	0.9500	0.8784	0.9804	0.2443	0.1787	0.3244
at least 1 HP	10	5	2	6	0.9500	0.8784	0.9804	0.2977	0.2261	0.3809
at least 1 HP	10	5	3	1	0.9875	0.9325	0.9978	0.1603	0.1073	0.2327
at least 1 HP	10	5	3	2	0.9875	0.9325	0.9978	0.1908	0.1327	0.2666
at least 1 HP	10	5	3	3	0.9625	0.8955	0.9872	0.2366	0.1720	0.3163
at least 1 HP	10	5	3	4	0.9625	0.8955	0.9872	0.2595	0.1921	0.3407
at least 1 HP	10	5	3	5	0.9500	0.8784	0.9804	0.2748	0.2056	0.3568
at least 1 HP	10	5	3	6	0.9500	0.8784	0.9804	0.3435	0.2677	0.4282
at least 1 HP	10	5	4	1	0.9875	0.9325	0.9978	0.1908	0.1327	0.2666
at least 1 HP	10	5	4	2	0.9875	0.9325	0.9978	0.2366	0.1720	0.3163
at least 1 HP	10	5	4	3	0.9500	0.8784	0.9804	0.2977	0.2261	0.3809
at least 1 HP	10	5	4	4	0.9500	0.8784	0.9804	0.3282	0.2537	0.4125

		Threshold				95% CI			95% CI	
Combination	EK	A	B	C	Sensitivity	Lower	Upper	Specificity	Lower	Upper
at least 1 HP	10	5	4	5	0.9250	0.8459	0.9652	0.3588	0.2817	0.4439
at least 1 HP	10	5	4	6	0.9125	0.8302	0.9570	0.4427	0.3605	0.5282
at least 1 HP	10	5	5	1	0.9875	0.9325	0.9978	0.1985	0.1392	0.2749
at least 1 HP	10	5	5	2	0.9875	0.9325	0.9978	0.2519	0.1854	0.3326
at least 1 HP	10	5	5	3	0.9500	0.8784	0.9804	0.3130	0.2399	0.3968
at least 1 HP	10	5	5	4	0.9500	0.8784	0.9804	0.3435	0.2677	0.4282
at least 1 HP	10	5	5	5	0.9250	0.8459	0.9652	0.3740	0.2959	0.4594
at least 1 HP	10	5	5	6	0.9000	0.8149	0.9485	0.4656	0.3824	0.5508
at least 1 HP	10	5	6	1	0.9875	0.9325	0.9978	0.2137	0.1522	0.2916
at least 1 HP	10	5	6	2	0.9875	0.9325	0.9978	0.2672	0.1988	0.3488
at least 1 HP	10	5	6	3	0.9500	0.8784	0.9804	0.3282	0.2537	0.4125
at least 1 HP	10	5	6	4	0.9500	0.8784	0.9804	0.3588	0.2817	0.4439
at least 1 HP	10	5	6	5	0.9250	0.8459	0.9652	0.3969	0.3172	0.4825
at least 1 HP	10	5	6	6	0.8875	0.7998	0.9397	0.4885	0.4045	0.5732
at least 1 HP	10	6	1	1	0.9875	0.9325	0.9978	0.1298	0.0826	0.1980
at least 1 HP	10	6	1	2	0.9875	0.9325	0.9978	0.1603	0.1073	0.2327
at least 1 HP	10	6	1	3	0.9625	0.8955	0.9872	0.1985	0.1392	0.2749
at least 1 HP	10	6	1	4	0.9625	0.8955	0.9872	0.2214	0.1588	0.2998
at least 1 HP	10	6	1	5	0.9625	0.8955	0.9872	0.2290	0.1654	0.3081
at least 1 HP	10	6	1	6	0.9625	0.8955	0.9872	0.2595	0.1921	0.3407
at least 1 HP	10	6	2	1	0.9875	0.9325	0.9978	0.1527	0.1011	0.2241
at least 1 HP	10	6	2	2	0.9875	0.9325	0.9978	0.1832	0.1263	0.2581
at least 1 HP	10	6	2	3	0.9625	0.8955	0.9872	0.2290	0.1654	0.3081
at least 1 HP	10	6	2	4	0.9625	0.8955	0.9872	0.2595	0.1921	0.3407
at least 1 HP	10	6	2	5	0.9500	0.8784	0.9804	0.2748	0.2056	0.3568
at least 1 HP	10	6	2	6	0.9500	0.8784	0.9804	0.3282	0.2537	0.4125
at least 1 HP	10	6	3	1	0.9875	0.9325	0.9978	0.1679	0.1136	0.2412
at least 1 HP	10	6	3	2	0.9875	0.9325	0.9978	0.1985	0.1392	0.2749
at least 1 HP	10	6	3	3	0.9625	0.8955	0.9872	0.2519	0.1854	0.3326
at least 1 HP	10	6	3	4	0.9625	0.8955	0.9872	0.2901	0.2192	0.3729
at least 1 HP	10	6	3	5	0.9500	0.8784	0.9804	0.3130	0.2399	0.3968
at least 1 HP	10	6	3	6	0.9500	0.8784	0.9804	0.3817	0.3030	0.4671
at least 1 HP	10	6	4	1	0.9875	0.9325	0.9978	0.1985	0.1392	0.2749
at least 1 HP	10	6	4	2	0.9875	0.9325	0.9978	0.2443	0.1787	0.3244
at least 1 HP	10	6	4	3	0.9500	0.8784	0.9804	0.3130	0.2399	0.3968
at least 1 HP	10	6	4	4	0.9500	0.8784	0.9804	0.3588	0.2817	0.4439
at least 1 HP	10	6	4	5	0.9250	0.8459	0.9652	0.3969	0.3172	0.4825
at least 1 HP	10	6	4	6	0.9125	0.8302	0.9570	0.4809	0.3971	0.5658
at least 1 HP	10	6	5	1	0.9875	0.9325	0.9978	0.2061	0.1457	0.2833
at least 1 HP	10	6	5	2	0.9875	0.9325	0.9978	0.2595	0.1921	0.3407
at least 1 HP	10	6	5	3	0.9500	0.8784	0.9804	0.3282	0.2537	0.4125
at least 1 HP	10	6	5	4	0.9500	0.8784	0.9804	0.3740	0.2959	0.4594
at least 1 HP	10	6	5	5	0.9250	0.8459	0.9652	0.4122	0.3316	0.4978
at least 1 HP	10	6	5	6	0.9000	0.8149	0.9485	0.5038	0.4193	0.5881
at least 1 HP	10	6	6	1	0.9875	0.9325	0.9978	0.2214	0.1588	0.2998
at least 1 HP	10	6	6	2	0.9875	0.9325	0.9978	0.2748	0.2056	0.3568
at least 1 HP	10	6	6	3	0.9500	0.8784	0.9804	0.3435	0.2677	0.4282
at least 1 HP	10	6	6	4	0.9500	0.8784	0.9804	0.3893	0.3101	0.4748
at least 1 HP	10	6	6	5	0.9250	0.8459	0.9652	0.4427	0.3605	0.5282
at least 1 HP	10	6	6	6	0.8875	0.7998	0.9397	0.5344	0.4492	0.6176
at least 2 HP	11	1	1	1	0.9750	0.9134	0.9931	0.2214	0.1588	0.2998
at least 2 HP	11	1	1	2	0.9750	0.9134	0.9931	0.2748	0.2056	0.3568
at least 2 HP	11	1	1	3	0.9375	0.8619	0.9730	0.3588	0.2817	0.4439

		Threshold				95% CI			95% CI	
Combination	EK	A	B	C	Sensitivity	Lower	Upper	Specificity	Lower	Upper
at least 2 HP	11	1	1	4	0.9375	0.8619	0.9730	0.3817	0.3030	0.4671
at least 2 HP	11	1	1	5	0.9250	0.8459	0.9652	0.4046	0.3244	0.4902
at least 2 HP	11	1	1	6	0.9125	0.8302	0.9570	0.4351	0.3533	0.5207
at least 2 HP	11	1	2	1	0.9500	0.8784	0.9804	0.2443	0.1787	0.3244
at least 2 HP	11	1	2	2	0.9500	0.8784	0.9804	0.3130	0.2399	0.3968
at least 2 HP	11	1	2	3	0.9125	0.8302	0.9570	0.3893	0.3101	0.4748
at least 2 HP	11	1	2	4	0.9125	0.8302	0.9570	0.4198	0.3388	0.5055
at least 2 HP	11	1	2	5	0.9125	0.8302	0.9570	0.4504	0.3678	0.5358
at least 2 HP	11	1	2	6	0.9000	0.8149	0.9485	0.5038	0.4193	0.5881
at least 2 HP	11	1	3	1	0.9500	0.8784	0.9804	0.2824	0.2124	0.3649
at least 2 HP	11	1	3	2	0.9500	0.8784	0.9804	0.3588	0.2817	0.4439
at least 2 HP	11	1	3	3	0.9125	0.8302	0.9570	0.4351	0.3533	0.5207
at least 2 HP	11	1	3	4	0.9125	0.8302	0.9570	0.4733	0.3898	0.5583
at least 2 HP	11	1	3	5	0.9125	0.8302	0.9570	0.5115	0.4268	0.5955
at least 2 HP	11	1	3	6	0.9000	0.8149	0.9485	0.5725	0.4869	0.6540
at least 2 HP	11	1	4	1	0.9375	0.8619	0.9730	0.3282	0.2537	0.4125
at least 2 HP	11	1	4	2	0.9375	0.8619	0.9730	0.4198	0.3388	0.5055
at least 2 HP	11	1	4	3	0.9125	0.8302	0.9570	0.4809	0.3971	0.5658
at least 2 HP	11	1	4	4	0.9125	0.8302	0.9570	0.5267	0.4417	0.6102
at least 2 HP	11	1	4	5	0.9000	0.8149	0.9485	0.5802	0.4945	0.6612
at least 2 HP	11	1	4	6	0.8750	0.7850	0.9307	0.6565	0.5718	0.7323
at least 2 HP	11	1	5	1	0.9250	0.8459	0.9652	0.3359	0.2607	0.4204
at least 2 HP	11	1	5	2	0.9250	0.8459	0.9652	0.4351	0.3533	0.5207
at least 2 HP	11	1	5	3	0.9000	0.8149	0.9485	0.4962	0.4119	0.5807
at least 2 HP	11	1	5	4	0.9000	0.8149	0.9485	0.5420	0.4567	0.6249
at least 2 HP	11	1	5	5	0.8875	0.7998	0.9397	0.5954	0.5098	0.6756
at least 2 HP	11	1	5	6	0.8750	0.7850	0.9307	0.6794	0.5953	0.7532
at least 2 HP	11	1	6	1	0.9125	0.8302	0.9570	0.3511	0.2747	0.4361
at least 2 HP	11	1	6	2	0.9125	0.8302	0.9570	0.4504	0.3678	0.5358
at least 2 HP	11	1	6	3	0.8875	0.7998	0.9397	0.5115	0.4268	0.5955
at least 2 HP	11	1	6	4	0.8875	0.7998	0.9397	0.5573	0.4718	0.6395
at least 2 HP	11	1	6	5	0.8750	0.7850	0.9307	0.6107	0.5252	0.6899
at least 2 HP	11	1	6	6	0.8500	0.7559	0.9121	0.6947	0.6112	0.7670
at least 2 HP	11	2	1	1	0.9625	0.8955	0.9872	0.2672	0.1988	0.3488
at least 2 HP	11	2	1	2	0.9625	0.8955	0.9872	0.3282	0.2537	0.4125
at least 2 HP	11	2	1	3	0.9375	0.8619	0.9730	0.4122	0.3316	0.4978
at least 2 HP	11	2	1	4	0.9375	0.8619	0.9730	0.4351	0.3533	0.5207
at least 2 HP	11	2	1	5	0.9250	0.8459	0.9652	0.4580	0.3751	0.5433
at least 2 HP	11	2	1	6	0.9000	0.8149	0.9485	0.4962	0.4119	0.5807
at least 2 HP	11	2	2	1	0.9375	0.8619	0.9730	0.3053	0.2330	0.3888
at least 2 HP	11	2	2	2	0.9375	0.8619	0.9730	0.3817	0.3030	0.4671
at least 2 HP	11	2	2	3	0.9125	0.8302	0.9570	0.4580	0.3751	0.5433
at least 2 HP	11	2	2	4	0.9125	0.8302	0.9570	0.4885	0.4045	0.5732
at least 2 HP	11	2	2	5	0.9125	0.8302	0.9570	0.5038	0.4193	0.5881
at least 2 HP	11	2	2	6	0.8875	0.7998	0.9397	0.5496	0.4642	0.6322
at least 2 HP	11	2	3	1	0.9375	0.8619	0.9730	0.3359	0.2607	0.4204
at least 2 HP	11	2	3	2	0.9375	0.8619	0.9730	0.4198	0.3388	0.5055
at least 2 HP	11	2	3	3	0.9125	0.8302	0.9570	0.4962	0.4119	0.5807
at least 2 HP	11	2	3	4	0.9125	0.8302	0.9570	0.5344	0.4492	0.6176
at least 2 HP	11	2	3	5	0.9125	0.8302	0.9570	0.5573	0.4718	0.6395
at least 2 HP	11	2	3	6	0.8875	0.7998	0.9397	0.6107	0.5252	0.6899
at least 2 HP	11	2	4	1	0.9250	0.8459	0.9652	0.3893	0.3101	0.4748
at least 2 HP	11	2	4	2	0.9250	0.8459	0.9652	0.4733	0.3898	0.5583

		Threshold				95% CI			95% CI	
Combination	EK	A	B	C	Sensitivity	Lower	Upper	Specificity	Lower	Upper
at least 2 HP	11	2	4	3	0.9125	0.8302	0.9570	0.5344	0.4492	0.6176
at least 2 HP	11	2	4	4	0.9125	0.8302	0.9570	0.5802	0.4945	0.6612
at least 2 HP	11	2	4	5	0.9000	0.8149	0.9485	0.6183	0.5329	0.6970
at least 2 HP	11	2	4	6	0.8625	0.7703	0.9215	0.6870	0.6032	0.7601
at least 2 HP	11	2	5	1	0.9125	0.8302	0.9570	0.3969	0.3172	0.4825
at least 2 HP	11	2	5	2	0.9125	0.8302	0.9570	0.4733	0.3898	0.5583
at least 2 HP	11	2	5	3	0.9000	0.8149	0.9485	0.5344	0.4492	0.6176
at least 2 HP	11	2	5	4	0.9000	0.8149	0.9485	0.5802	0.4945	0.6612
at least 2 HP	11	2	5	5	0.8875	0.7998	0.9397	0.6183	0.5329	0.6970
at least 2 HP	11	2	5	6	0.8625	0.7703	0.9215	0.6947	0.6112	0.7670
at least 2 HP	11	2	6	1	0.9000	0.8149	0.9485	0.4122	0.3316	0.4978
at least 2 HP	11	2	6	2	0.9000	0.8149	0.9485	0.4885	0.4045	0.5732
at least 2 HP	11	2	6	3	0.8875	0.7998	0.9397	0.5496	0.4642	0.6322
at least 2 HP	11	2	6	4	0.8875	0.7998	0.9397	0.5954	0.5098	0.6756
at least 2 HP	11	2	6	5	0.8750	0.7850	0.9307	0.6336	0.5484	0.7112
at least 2 HP	11	2	6	6	0.8375	0.7416	0.9025	0.7099	0.6271	0.7808
at least 2 HP	11	3	1	1	0.9625	0.8955	0.9872	0.3053	0.2330	0.3888
at least 2 HP	11	3	1	2	0.9625	0.8955	0.9872	0.3740	0.2959	0.4594
at least 2 HP	11	3	1	3	0.9375	0.8619	0.9730	0.4504	0.3678	0.5358
at least 2 HP	11	3	1	4	0.9375	0.8619	0.9730	0.4733	0.3898	0.5583
at least 2 HP	11	3	1	5	0.9250	0.8459	0.9652	0.5115	0.4268	0.5955
at least 2 HP	11	3	1	6	0.9000	0.8149	0.9485	0.5649	0.4793	0.6467
at least 2 HP	11	3	2	1	0.9375	0.8619	0.9730	0.3664	0.2888	0.4516
at least 2 HP	11	3	2	2	0.9375	0.8619	0.9730	0.4504	0.3678	0.5358
at least 2 HP	11	3	2	3	0.9125	0.8302	0.9570	0.5191	0.4342	0.6029
at least 2 HP	11	3	2	4	0.9125	0.8302	0.9570	0.5496	0.4642	0.6322
at least 2 HP	11	3	2	5	0.9125	0.8302	0.9570	0.5802	0.4945	0.6612
at least 2 HP	11	3	2	6	0.8875	0.7998	0.9397	0.6260	0.5406	0.7041
at least 2 HP	11	3	3	1	0.9375	0.8619	0.9730	0.4198	0.3388	0.5055
at least 2 HP	11	3	3	2	0.9375	0.8619	0.9730	0.5115	0.4268	0.5955
at least 2 HP	11	3	3	3	0.9125	0.8302	0.9570	0.5802	0.4945	0.6612
at least 2 HP	11	3	3	4	0.9125	0.8302	0.9570	0.6031	0.5175	0.6828
at least 2 HP	11	3	3	5	0.9125	0.8302	0.9570	0.6412	0.5561	0.7183
at least 2 HP	11	3	3	6	0.8875	0.7998	0.9397	0.6947	0.6112	0.7670
at least 2 HP	11	3	4	1	0.9250	0.8459	0.9652	0.4885	0.4045	0.5732
at least 2 HP	11	3	4	2	0.9250	0.8459	0.9652	0.5649	0.4793	0.6467
at least 2 HP	11	3	4	3	0.9125	0.8302	0.9570	0.6183	0.5329	0.6970
at least 2 HP	11	3	4	4	0.9125	0.8302	0.9570	0.6489	0.5639	0.7253
at least 2 HP	11	3	4	5	0.9000	0.8149	0.9485	0.6718	0.5875	0.7463
at least 2 HP	11	3	4	6	0.8625	0.7703	0.9215	0.7405	0.6593	0.8079
at least 2 HP	11	3	5	1	0.9125	0.8302	0.9570	0.5115	0.4268	0.5955
at least 2 HP	11	3	5	2	0.9125	0.8302	0.9570	0.5802	0.4945	0.6612
at least 2 HP	11	3	5	3	0.9000	0.8149	0.9485	0.6336	0.5484	0.7112
at least 2 HP	11	3	5	4	0.9000	0.8149	0.9485	0.6641	0.5796	0.7393
at least 2 HP	11	3	5	5	0.8875	0.7998	0.9397	0.6870	0.6032	0.7601
at least 2 HP	11	3	5	6	0.8625	0.7703	0.9215	0.7481	0.6674	0.8146
at least 2 HP	11	3	6	1	0.9000	0.8149	0.9485	0.5191	0.4342	0.6029
at least 2 HP	11	3	6	2	0.9000	0.8149	0.9485	0.5878	0.5022	0.6684
at least 2 HP	11	3	6	3	0.8875	0.7998	0.9397	0.6412	0.5561	0.7183
at least 2 HP	11	3	6	4	0.8875	0.7998	0.9397	0.6718	0.5875	0.7463
at least 2 HP	11	3	6	5	0.8750	0.7850	0.9307	0.6947	0.6112	0.7670
at least 2 HP	11	3	6	6	0.8375	0.7416	0.9025	0.7557	0.6756	0.8213
at least 2 HP	11	4	1	1	0.9500	0.8784	0.9804	0.3740	0.2959	0.4594

		Threshold				95% CI			95% CI	
Combination	EK	A	B	C	Sensitivity	Lower	Upper	Specificity	Lower	Upper
at least 2 HP	11	4	1	2	0.9500	0.8784	0.9804	0.4198	0.3388	0.5055
at least 2 HP	11	4	1	3	0.9375	0.8619	0.9730	0.4809	0.3971	0.5658
at least 2 HP	11	4	1	4	0.9375	0.8619	0.9730	0.5191	0.4342	0.6029
at least 2 HP	11	4	1	5	0.9125	0.8302	0.9570	0.5573	0.4718	0.6395
at least 2 HP	11	4	1	6	0.8750	0.7850	0.9307	0.6260	0.5406	0.7041
at least 2 HP	11	4	2	1	0.9250	0.8459	0.9652	0.4427	0.3605	0.5282
at least 2 HP	11	4	2	2	0.9250	0.8459	0.9652	0.5038	0.4193	0.5881
at least 2 HP	11	4	2	3	0.9125	0.8302	0.9570	0.5573	0.4718	0.6395
at least 2 HP	11	4	2	4	0.9125	0.8302	0.9570	0.5878	0.5022	0.6684
at least 2 HP	11	4	2	5	0.9000	0.8149	0.9485	0.6183	0.5329	0.6970
at least 2 HP	11	4	2	6	0.8625	0.7703	0.9215	0.6794	0.5953	0.7532
at least 2 HP	11	4	3	1	0.9250	0.8459	0.9652	0.5115	0.4268	0.5955
at least 2 HP	11	4	3	2	0.9250	0.8459	0.9652	0.5802	0.4945	0.6612
at least 2 HP	11	4	3	3	0.9125	0.8302	0.9570	0.6336	0.5484	0.7112
at least 2 HP	11	4	3	4	0.9125	0.8302	0.9570	0.6565	0.5718	0.7323
at least 2 HP	11	4	3	5	0.9000	0.8149	0.9485	0.6947	0.6112	0.7670
at least 2 HP	11	4	3	6	0.8625	0.7703	0.9215	0.7634	0.6837	0.8280
at least 2 HP	11	4	4	1	0.8875	0.7998	0.9397	0.5954	0.5098	0.6756
at least 2 HP	11	4	4	2	0.8875	0.7998	0.9397	0.6489	0.5639	0.7253
at least 2 HP	11	4	4	3	0.8875	0.7998	0.9397	0.6870	0.6032	0.7601
at least 2 HP	11	4	4	4	0.8875	0.7998	0.9397	0.7023	0.6191	0.7739
at least 2 HP	11	4	4	5	0.8875	0.7998	0.9397	0.7252	0.6432	0.7944
at least 2 HP	11	4	4	6	0.8625	0.7703	0.9215	0.7939	0.7167	0.8543
at least 2 HP	11	4	5	1	0.8750	0.7850	0.9307	0.6183	0.5329	0.6970
at least 2 HP	11	4	5	2	0.8750	0.7850	0.9307	0.6641	0.5796	0.7393
at least 2 HP	11	4	5	3	0.8750	0.7850	0.9307	0.7023	0.6191	0.7739
at least 2 HP	11	4	5	4	0.8750	0.7850	0.9307	0.7176	0.6351	0.7876
at least 2 HP	11	4	5	5	0.8750	0.7850	0.9307	0.7405	0.6593	0.8079
at least 2 HP	11	4	5	6	0.8625	0.7703	0.9215	0.8015	0.7251	0.8608
at least 2 HP	11	4	6	1	0.8625	0.7703	0.9215	0.6260	0.5406	0.7041
at least 2 HP	11	4	6	2	0.8625	0.7703	0.9215	0.6718	0.5875	0.7463
at least 2 HP	11	4	6	3	0.8625	0.7703	0.9215	0.7099	0.6271	0.7808
at least 2 HP	11	4	6	4	0.8625	0.7703	0.9215	0.7252	0.6432	0.7944
at least 2 HP	11	4	6	5	0.8625	0.7703	0.9215	0.7481	0.6674	0.8146
at least 2 HP	11	4	6	6	0.8375	0.7416	0.9025	0.8092	0.7334	0.8673
at least 2 HP	11	5	1	1	0.9500	0.8784	0.9804	0.3969	0.3172	0.4825
at least 2 HP	11	5	1	2	0.9500	0.8784	0.9804	0.4427	0.3605	0.5282
at least 2 HP	11	5	1	3	0.9375	0.8619	0.9730	0.5038	0.4193	0.5881
at least 2 HP	11	5	1	4	0.9375	0.8619	0.9730	0.5420	0.4567	0.6249
at least 2 HP	11	5	1	5	0.9125	0.8302	0.9570	0.5802	0.4945	0.6612
at least 2 HP	11	5	1	6	0.8625	0.7703	0.9215	0.6718	0.5875	0.7463
at least 2 HP	11	5	2	1	0.9250	0.8459	0.9652	0.4809	0.3971	0.5658
at least 2 HP	11	5	2	2	0.9250	0.8459	0.9652	0.5420	0.4567	0.6249
at least 2 HP	11	5	2	3	0.9125	0.8302	0.9570	0.5954	0.5098	0.6756
at least 2 HP	11	5	2	4	0.9125	0.8302	0.9570	0.6260	0.5406	0.7041
at least 2 HP	11	5	2	5	0.9000	0.8149	0.9485	0.6565	0.5718	0.7323
at least 2 HP	11	5	2	6	0.8500	0.7559	0.9121	0.7252	0.6432	0.7944
at least 2 HP	11	5	3	1	0.9250	0.8459	0.9652	0.5649	0.4793	0.6467
at least 2 HP	11	5	3	2	0.9250	0.8459	0.9652	0.6336	0.5484	0.7112
at least 2 HP	11	5	3	3	0.9125	0.8302	0.9570	0.6870	0.6032	0.7601
at least 2 HP	11	5	3	4	0.9125	0.8302	0.9570	0.7099	0.6271	0.7808
at least 2 HP	11	5	3	5	0.9000	0.8149	0.9485	0.7481	0.6674	0.8146
at least 2 HP	11	5	3	6	0.8500	0.7559	0.9121	0.8092	0.7334	0.8673

		Threshold				95% CI			95% CI	
Combination	EK	A	B	C	Sensitivity	Lower	Upper	Specificity	Lower	Upper
at least 2 HP	11	5	4	1	0.8750	0.7850	0.9307	0.6565	0.5718	0.7323
at least 2 HP	11	5	4	2	0.8750	0.7850	0.9307	0.7099	0.6271	0.7808
at least 2 HP	11	5	4	3	0.8750	0.7850	0.9307	0.7481	0.6674	0.8146
at least 2 HP	11	5	4	4	0.8750	0.7850	0.9307	0.7634	0.6837	0.8280
at least 2 HP	11	5	4	5	0.8750	0.7850	0.9307	0.7863	0.7084	0.8478
at least 2 HP	11	5	4	6	0.8375	0.7416	0.9025	0.8321	0.7588	0.8864
at least 2 HP	11	5	5	1	0.8625	0.7703	0.9215	0.6794	0.5953	0.7532
at least 2 HP	11	5	5	2	0.8625	0.7703	0.9215	0.7252	0.6432	0.7944
at least 2 HP	11	5	5	3	0.8625	0.7703	0.9215	0.7634	0.6837	0.8280
at least 2 HP	11	5	5	4	0.8625	0.7703	0.9215	0.7786	0.7002	0.8412
at least 2 HP	11	5	5	5	0.8625	0.7703	0.9215	0.8015	0.7251	0.8608
at least 2 HP	11	5	5	6	0.8375	0.7416	0.9025	0.8397	0.7673	0.8927
at least 2 HP	11	5	6	1	0.8375	0.7416	0.9025	0.6870	0.6032	0.7601
at least 2 HP	11	5	6	2	0.8375	0.7416	0.9025	0.7328	0.6512	0.8012
at least 2 HP	11	5	6	3	0.8375	0.7416	0.9025	0.7710	0.6919	0.8346
at least 2 HP	11	5	6	4	0.8375	0.7416	0.9025	0.7863	0.7084	0.8478
at least 2 HP	11	5	6	5	0.8375	0.7416	0.9025	0.8092	0.7334	0.8673
at least 2 HP	11	5	6	6	0.8250	0.7274	0.8928	0.8473	0.7759	0.8989
at least 2 HP	11	6	1	1	0.9500	0.8784	0.9804	0.4275	0.3460	0.5131
at least 2 HP	11	6	1	2	0.9500	0.8784	0.9804	0.4809	0.3971	0.5658
at least 2 HP	11	6	1	3	0.9375	0.8619	0.9730	0.5420	0.4567	0.6249
at least 2 HP	11	6	1	4	0.9375	0.8619	0.9730	0.5649	0.4793	0.6467
at least 2 HP	11	6	1	5	0.9125	0.8302	0.9570	0.6183	0.5329	0.6970
at least 2 HP	11	6	1	6	0.8500	0.7559	0.9121	0.7099	0.6271	0.7808
at least 2 HP	11	6	2	1	0.9250	0.8459	0.9652	0.5115	0.4268	0.5955
at least 2 HP	11	6	2	2	0.9250	0.8459	0.9652	0.5802	0.4945	0.6612
at least 2 HP	11	6	2	3	0.9125	0.8302	0.9570	0.6336	0.5484	0.7112
at least 2 HP	11	6	2	4	0.9125	0.8302	0.9570	0.6489	0.5639	0.7253
at least 2 HP	11	6	2	5	0.9000	0.8149	0.9485	0.6947	0.6112	0.7670
at least 2 HP	11	6	2	6	0.8375	0.7416	0.9025	0.7634	0.6837	0.8280
at least 2 HP	11	6	3	1	0.9250	0.8459	0.9652	0.6107	0.5252	0.6899
at least 2 HP	11	6	3	2	0.9250	0.8459	0.9652	0.6870	0.6032	0.7601
at least 2 HP	11	6	3	3	0.9125	0.8302	0.9570	0.7405	0.6593	0.8079
at least 2 HP	11	6	3	4	0.9125	0.8302	0.9570	0.7481	0.6674	0.8146
at least 2 HP	11	6	3	5	0.9000	0.8149	0.9485	0.7863	0.7084	0.8478
at least 2 HP	11	6	3	6	0.8375	0.7416	0.9025	0.8473	0.7759	0.8989
at least 2 HP	11	6	4	1	0.8750	0.7850	0.9307	0.7023	0.6191	0.7739
at least 2 HP	11	6	4	2	0.8750	0.7850	0.9307	0.7634	0.6837	0.8280
at least 2 HP	11	6	4	3	0.8750	0.7850	0.9307	0.8015	0.7251	0.8608
at least 2 HP	11	6	4	4	0.8750	0.7850	0.9307	0.8015	0.7251	0.8608
at least 2 HP	11	6	4	5	0.8750	0.7850	0.9307	0.8244	0.7503	0.8801
at least 2 HP	11	6	4	6	0.8250	0.7274	0.8928	0.8702	0.8020	0.9174
at least 2 HP	11	6	5	1	0.8625	0.7703	0.9215	0.7252	0.6432	0.7944
at least 2 HP	11	6	5	2	0.8625	0.7703	0.9215	0.7786	0.7002	0.8412
at least 2 HP	11	6	5	3	0.8625	0.7703	0.9215	0.8168	0.7419	0.8737
at least 2 HP	11	6	5	4	0.8625	0.7703	0.9215	0.8168	0.7419	0.8737
at least 2 HP	11	6	5	5	0.8625	0.7703	0.9215	0.8397	0.7673	0.8927
at least 2 HP	11	6	5	6	0.8250	0.7274	0.8928	0.8779	0.8108	0.9234
at least 2 HP	11	6	6	1	0.8375	0.7416	0.9025	0.7405	0.6593	0.8079
at least 2 HP	11	6	6	2	0.8375	0.7416	0.9025	0.7939	0.7167	0.8543
at least 2 HP	11	6	6	3	0.8375	0.7416	0.9025	0.8321	0.7588	0.8864
at least 2 HP	11	6	6	4	0.8375	0.7416	0.9025	0.8321	0.7588	0.8864
at least 2 HP	11	6	6	5	0.8375	0.7416	0.9025	0.8397	0.7673	0.8927

		Threshold				95% CI			95% CI	
Combination	EK	A	B	C	Sensitivity	Lower	Upper	Specificity	Lower	Upper
at least 2 HP	11	6	6	6	0.8125	0.7134	0.8829	0.8779	0.8108	0.9234
all HP	12	1	1	1	0.8750	0.7850	0.9307	0.6031	0.5175	0.6828
all HP	12	1	1	2	0.8750	0.7850	0.9307	0.6641	0.5796	0.7393
all HP	12	1	1	3	0.8750	0.7850	0.9307	0.6794	0.5953	0.7532
all HP	12	1	1	4	0.8750	0.7850	0.9307	0.7023	0.6191	0.7739
all HP	12	1	1	5	0.8625	0.7703	0.9215	0.7405	0.6593	0.8079
all HP	12	1	1	6	0.7875	0.6858	0.8629	0.8168	0.7419	0.8737
all HP	12	1	2	1	0.8500	0.7559	0.9121	0.6870	0.6032	0.7601
all HP	12	1	2	2	0.8500	0.7559	0.9121	0.7328	0.6512	0.8012
all HP	12	1	2	3	0.8500	0.7559	0.9121	0.7481	0.6674	0.8146
all HP	12	1	2	4	0.8500	0.7559	0.9121	0.7634	0.6837	0.8280
all HP	12	1	2	5	0.8375	0.7416	0.9025	0.7939	0.7167	0.8543
all HP	12	1	2	6	0.7625	0.6586	0.8424	0.8473	0.7759	0.8989
all HP	12	1	3	1	0.8500	0.7559	0.9121	0.7863	0.7084	0.8478
all HP	12	1	3	2	0.8500	0.7559	0.9121	0.8244	0.7503	0.8801
all HP	12	1	3	3	0.8500	0.7559	0.9121	0.8321	0.7588	0.8864
all HP	12	1	3	4	0.8500	0.7559	0.9121	0.8397	0.7673	0.8927
all HP	12	1	3	5	0.8375	0.7416	0.9025	0.8626	0.7932	0.9113
all HP	12	1	3	6	0.7625	0.6586	0.8424	0.9008	0.8376	0.9411
all HP	12	1	4	1	0.7875	0.6858	0.8629	0.8550	0.7846	0.9051
all HP	12	1	4	2	0.7875	0.6858	0.8629	0.8779	0.8108	0.9234
all HP	12	1	4	3	0.7875	0.6858	0.8629	0.8855	0.8197	0.9294
all HP	12	1	4	4	0.7875	0.6858	0.8629	0.8855	0.8197	0.9294
all HP	12	1	4	5	0.7875	0.6858	0.8629	0.8931	0.8286	0.9353
all HP	12	1	4	6	0.7250	0.6186	0.8108	0.9160	0.8559	0.9525
all HP	12	1	5	1	0.7875	0.6858	0.8629	0.8779	0.8108	0.9234
all HP	12	1	5	2	0.7875	0.6858	0.8629	0.8931	0.8286	0.9353
all HP	12	1	5	3	0.7875	0.6858	0.8629	0.9008	0.8376	0.9411
all HP	12	1	5	4	0.7875	0.6858	0.8629	0.9008	0.8376	0.9411
all HP	12	1	5	5	0.7875	0.6858	0.8629	0.9084	0.8467	0.9468
all HP	12	1	5	6	0.7250	0.6186	0.8108	0.9237	0.8652	0.9580
all HP	12	1	6	1	0.7250	0.6186	0.8108	0.8931	0.8286	0.9353
all HP	12	1	6	2	0.7250	0.6186	0.8108	0.9084	0.8467	0.9468
all HP	12	1	6	3	0.7250	0.6186	0.8108	0.9160	0.8559	0.9525
all HP	12	1	6	4	0.7250	0.6186	0.8108	0.9160	0.8559	0.9525
all HP	12	1	6	5	0.7250	0.6186	0.8108	0.9160	0.8559	0.9525
all HP	12	1	6	6	0.6750	0.5664	0.7676	0.9313	0.8746	0.9634
all HP	12	2	1	1	0.8625	0.7703	0.9215	0.6336	0.5484	0.7112
all HP	12	2	1	2	0.8625	0.7703	0.9215	0.6794	0.5953	0.7532
all HP	12	2	1	3	0.8625	0.7703	0.9215	0.6947	0.6112	0.7670
all HP	12	2	1	4	0.8625	0.7703	0.9215	0.7176	0.6351	0.7876
all HP	12	2	1	5	0.8500	0.7559	0.9121	0.7481	0.6674	0.8146
all HP	12	2	1	6	0.7875	0.6858	0.8629	0.8168	0.7419	0.8737
all HP	12	2	2	1	0.8375	0.7416	0.9025	0.7023	0.6191	0.7739
all HP	12	2	2	2	0.8375	0.7416	0.9025	0.7328	0.6512	0.8012
all HP	12	2	2	3	0.8375	0.7416	0.9025	0.7481	0.6674	0.8146
all HP	12	2	2	4	0.8375	0.7416	0.9025	0.7634	0.6837	0.8280
all HP	12	2	2	5	0.8250	0.7274	0.8928	0.7939	0.7167	0.8543
all HP	12	2	2	6	0.7625	0.6586	0.8424	0.8473	0.7759	0.8989
all HP	12	2	3	1	0.8375	0.7416	0.9025	0.8015	0.7251	0.8608
all HP	12	2	3	2	0.8375	0.7416	0.9025	0.8244	0.7503	0.8801
all HP	12	2	3	3	0.8375	0.7416	0.9025	0.8321	0.7588	0.8864
all HP	12	2	3	4	0.8375	0.7416	0.9025	0.8397	0.7673	0.8927

Combination	EK	Threshold A	B	C	Sensitivity	95% CI Lower	Upper	Specificity	95% CI Lower	Upper
all HP	12	2	3	5	0.8250	0.7274	0.8928	0.8626	0.7932	0.9113
all HP	12	2	3	6	0.7625	0.6586	0.8424	0.9008	0.8376	0.9411
all HP	12	2	4	1	0.7750	0.6721	0.8527	0.8626	0.7932	0.9113
all HP	12	2	4	2	0.7750	0.6721	0.8527	0.8779	0.8108	0.9234
all HP	12	2	4	3	0.7750	0.6721	0.8527	0.8855	0.8197	0.9294
all HP	12	2	4	4	0.7750	0.6721	0.8527	0.8855	0.8197	0.9294
all HP	12	2	4	5	0.7750	0.6721	0.8527	0.8931	0.8286	0.9353
all HP	12	2	4	6	0.7250	0.6186	0.8108	0.9160	0.8559	0.9525
all HP	12	2	5	1	0.7750	0.6721	0.8527	0.8779	0.8108	0.9234
all HP	12	2	5	2	0.7750	0.6721	0.8527	0.8931	0.8286	0.9353
all HP	12	2	5	3	0.7750	0.6721	0.8527	0.9008	0.8376	0.9411
all HP	12	2	5	4	0.7750	0.6721	0.8527	0.9008	0.8376	0.9411
all HP	12	2	5	5	0.7750	0.6721	0.8527	0.9084	0.8467	0.9468
all HP	12	2	5	6	0.7250	0.6186	0.8108	0.9237	0.8652	0.9580
all HP	12	2	6	1	0.7125	0.6054	0.8001	0.8931	0.8286	0.9353
all HP	12	2	6	2	0.7125	0.6054	0.8001	0.9084	0.8467	0.9468
all HP	12	2	6	3	0.7125	0.6054	0.8001	0.9160	0.8559	0.9525
all HP	12	2	6	4	0.7125	0.6054	0.8001	0.9160	0.8559	0.9525
all HP	12	2	6	5	0.7125	0.6054	0.8001	0.9160	0.8559	0.9525
all HP	12	2	6	6	0.6750	0.5664	0.7676	0.9313	0.8746	0.9634
all HP	12	3	1	1	0.8500	0.7559	0.9121	0.7176	0.6351	0.7876
all HP	12	3	1	2	0.8500	0.7559	0.9121	0.7557	0.6756	0.8213
all HP	12	3	1	3	0.8500	0.7559	0.9121	0.7710	0.6919	0.8346
all HP	12	3	1	4	0.8500	0.7559	0.9121	0.7863	0.7084	0.8478
all HP	12	3	1	5	0.8375	0.7416	0.9025	0.8015	0.7251	0.8608
all HP	12	3	1	6	0.7750	0.6721	0.8527	0.8550	0.7846	0.9051
all HP	12	3	2	1	0.8250	0.7274	0.8928	0.7634	0.6837	0.8280
all HP	12	3	2	2	0.8250	0.7274	0.8928	0.7863	0.7084	0.8478
all HP	12	3	2	3	0.8250	0.7274	0.8928	0.8015	0.7251	0.8608
all HP	12	3	2	4	0.8250	0.7274	0.8928	0.8092	0.7334	0.8673
all HP	12	3	2	5	0.8125	0.7134	0.8829	0.8244	0.7503	0.8801
all HP	12	3	2	6	0.7500	0.6452	0.8319	0.8702	0.8020	0.9174
all HP	12	3	3	1	0.8250	0.7274	0.8928	0.8397	0.7673	0.8927
all HP	12	3	3	2	0.8250	0.7274	0.8928	0.8550	0.7846	0.9051
all HP	12	3	3	3	0.8250	0.7274	0.8928	0.8626	0.7932	0.9113
all HP	12	3	3	4	0.8250	0.7274	0.8928	0.8702	0.8020	0.9174
all HP	12	3	3	5	0.8125	0.7134	0.8829	0.8779	0.8108	0.9234
all HP	12	3	3	6	0.7500	0.6452	0.8319	0.9084	0.8467	0.9468
all HP	12	3	4	1	0.7625	0.6586	0.8424	0.8779	0.8108	0.9234
all HP	12	3	4	2	0.7625	0.6586	0.8424	0.8931	0.8286	0.9353
all HP	12	3	4	3	0.7625	0.6586	0.8424	0.9008	0.8376	0.9411
all HP	12	3	4	4	0.7625	0.6586	0.8424	0.9008	0.8376	0.9411
all HP	12	3	4	5	0.7625	0.6586	0.8424	0.9084	0.8467	0.9468
all HP	12	3	4	6	0.7125	0.6054	0.8001	0.9237	0.8652	0.9580
all HP	12	3	5	1	0.7625	0.6586	0.8424	0.8779	0.8108	0.9234
all HP	12	3	5	2	0.7625	0.6586	0.8424	0.8931	0.8286	0.9353
all HP	12	3	5	3	0.7625	0.6586	0.8424	0.9008	0.8376	0.9411
all HP	12	3	5	4	0.7625	0.6586	0.8424	0.9008	0.8376	0.9411
all HP	12	3	5	5	0.7625	0.6586	0.8424	0.9084	0.8467	0.9468
all HP	12	3	5	6	0.7125	0.6054	0.8001	0.9237	0.8652	0.9580
all HP	12	3	6	1	0.7000	0.5923	0.7894	0.8931	0.8286	0.9353
all HP	12	3	6	2	0.7000	0.5923	0.7894	0.9084	0.8467	0.9468
all HP	12	3	6	3	0.7000	0.5923	0.7894	0.9160	0.8559	0.9525

		Threshold				95% CI			95% CI	
Combination	EK	A	B	C	Sensitivity	Lower	Upper	Specificity	Lower	Upper
all HP	12	3	6	4	0.7000	0.5923	0.7894	0.9160	0.8559	0.9525
all HP	12	3	6	5	0.7000	0.5923	0.7894	0.9160	0.8559	0.9525
all HP	12	3	6	6	0.6625	0.5536	0.7565	0.9313	0.8746	0.9634
all HP	12	4	1	1	0.8125	0.7134	0.8829	0.7786	0.7002	0.8412
all HP	12	4	1	2	0.8125	0.7134	0.8829	0.8168	0.7419	0.8737
all HP	12	4	1	3	0.8125	0.7134	0.8829	0.8321	0.7588	0.8864
all HP	12	4	1	4	0.8125	0.7134	0.8829	0.8321	0.7588	0.8864
all HP	12	4	1	5	0.8125	0.7134	0.8829	0.8473	0.7759	0.8989
all HP	12	4	1	6	0.7625	0.6586	0.8424	0.8855	0.8197	0.9294
all HP	12	4	2	1	0.7875	0.6858	0.8629	0.8168	0.7419	0.8737
all HP	12	4	2	2	0.7875	0.6858	0.8629	0.8397	0.7673	0.8927
all HP	12	4	2	3	0.7875	0.6858	0.8629	0.8550	0.7846	0.9051
all HP	12	4	2	4	0.7875	0.6858	0.8629	0.8550	0.7846	0.9051
all HP	12	4	2	5	0.7875	0.6858	0.8629	0.8702	0.8020	0.9174
all HP	12	4	2	6	0.7375	0.6318	0.8214	0.9008	0.8376	0.9411
all HP	12	4	3	1	0.7875	0.6858	0.8629	0.8779	0.8108	0.9234
all HP	12	4	3	2	0.7875	0.6858	0.8629	0.8931	0.8286	0.9353
all HP	12	4	3	3	0.7875	0.6858	0.8629	0.9008	0.8376	0.9411
all HP	12	4	3	4	0.7875	0.6858	0.8629	0.9008	0.8376	0.9411
all HP	12	4	3	5	0.7875	0.6858	0.8629	0.9084	0.8467	0.9468
all HP	12	4	3	6	0.7375	0.6318	0.8214	0.9237	0.8652	0.9580
all HP	12	4	4	1	0.7500	0.6452	0.8319	0.8855	0.8197	0.9294
all HP	12	4	4	2	0.7500	0.6452	0.8319	0.9008	0.8376	0.9411
all HP	12	4	4	3	0.7500	0.6452	0.8319	0.9084	0.8467	0.9468
all HP	12	4	4	4	0.7500	0.6452	0.8319	0.9084	0.8467	0.9468
all HP	12	4	4	5	0.7500	0.6452	0.8319	0.9160	0.8559	0.9525
all HP	12	4	4	6	0.7000	0.5923	0.7894	0.9237	0.8652	0.9580
all HP	12	4	5	1	0.7500	0.6452	0.8319	0.8855	0.8197	0.9294
all HP	12	4	5	2	0.7500	0.6452	0.8319	0.9008	0.8376	0.9411
all HP	12	4	5	3	0.7500	0.6452	0.8319	0.9084	0.8467	0.9468
all HP	12	4	5	4	0.7500	0.6452	0.8319	0.9084	0.8467	0.9468
all HP	12	4	5	5	0.7500	0.6452	0.8319	0.9160	0.8559	0.9525
all HP	12	4	5	6	0.7000	0.5923	0.7894	0.9237	0.8652	0.9580
all HP	12	4	6	1	0.6875	0.5793	0.7785	0.9008	0.8376	0.9411
all HP	12	4	6	2	0.6875	0.5793	0.7785	0.9160	0.8559	0.9525
all HP	12	4	6	3	0.6875	0.5793	0.7785	0.9237	0.8652	0.9580
all HP	12	4	6	4	0.6875	0.5793	0.7785	0.9237	0.8652	0.9580
all HP	12	4	6	5	0.6875	0.5793	0.7785	0.9237	0.8652	0.9580
all HP	12	4	6	6	0.6500	0.5408	0.7455	0.9313	0.8746	0.9634
all HP	12	5	1	1	0.7875	0.6858	0.8629	0.8321	0.7588	0.8864
all HP	12	5	1	2	0.7875	0.6858	0.8629	0.8702	0.8020	0.9174
all HP	12	5	1	3	0.7875	0.6858	0.8629	0.8855	0.8197	0.9294
all HP	12	5	1	4	0.7875	0.6858	0.8629	0.8855	0.8197	0.9294
all HP	12	5	1	5	0.7875	0.6858	0.8629	0.9008	0.8376	0.9411
all HP	12	5	1	6	0.7500	0.6452	0.8319	0.9160	0.8559	0.9525
all HP	12	5	2	1	0.7625	0.6586	0.8424	0.8550	0.7846	0.9051
all HP	12	5	2	2	0.7625	0.6586	0.8424	0.8779	0.8108	0.9234
all HP	12	5	2	3	0.7625	0.6586	0.8424	0.8931	0.8286	0.9353
all HP	12	5	2	4	0.7625	0.6586	0.8424	0.8931	0.8286	0.9353
all HP	12	5	2	5	0.7625	0.6586	0.8424	0.9084	0.8467	0.9468
all HP	12	5	2	6	0.7250	0.6186	0.8108	0.9237	0.8652	0.9580
all HP	12	5	3	1	0.7625	0.6586	0.8424	0.8931	0.8286	0.9353
all HP	12	5	3	2	0.7625	0.6586	0.8424	0.9084	0.8467	0.9468

		Threshold				95% CI			95% CI	
Combination	EK	A	B	C	Sensitivity	Lower	Upper	Specificity	Lower	Upper
all HP	12	5	3	3	0.7625	0.6586	0.8424	0.9160	0.8559	0.9525
all HP	12	5	3	4	0.7625	0.6586	0.8424	0.9160	0.8559	0.9525
all HP	12	5	3	5	0.7625	0.6586	0.8424	0.9237	0.8652	0.9580
all HP	12	5	3	6	0.7250	0.6186	0.8108	0.9313	0.8746	0.9634
all HP	12	5	4	1	0.7375	0.6318	0.8214	0.8931	0.8286	0.9353
all HP	12	5	4	2	0.7375	0.6318	0.8214	0.9084	0.8467	0.9468
all HP	12	5	4	3	0.7375	0.6318	0.8214	0.9160	0.8559	0.9525
all HP	12	5	4	4	0.7375	0.6318	0.8214	0.9160	0.8559	0.9525
all HP	12	5	4	5	0.7375	0.6318	0.8214	0.9237	0.8652	0.9580
all HP	12	5	4	6	0.7000	0.5923	0.7894	0.9313	0.8746	0.9634
all HP	12	5	5	1	0.7375	0.6318	0.8214	0.8931	0.8286	0.9353
all HP	12	5	5	2	0.7375	0.6318	0.8214	0.9084	0.8467	0.9468
all HP	12	5	5	3	0.7375	0.6318	0.8214	0.9160	0.8559	0.9525
all HP	12	5	5	4	0.7375	0.6318	0.8214	0.9160	0.8559	0.9525
all HP	12	5	5	5	0.7375	0.6318	0.8214	0.9237	0.8652	0.9580
all HP	12	5	5	6	0.7000	0.5923	0.7894	0.9313	0.8746	0.9634
all HP	12	5	6	1	0.6875	0.5793	0.7785	0.9084	0.8467	0.9468
all HP	12	5	6	2	0.6875	0.5793	0.7785	0.9237	0.8652	0.9580
all HP	12	5	6	3	0.6875	0.5793	0.7785	0.9313	0.8746	0.9634
all HP	12	5	6	4	0.6875	0.5793	0.7785	0.9313	0.8746	0.9634
all HP	12	5	6	5	0.6875	0.5793	0.7785	0.9313	0.8746	0.9634
all HP	12	5	6	6	0.6500	0.5408	0.7455	0.9389	0.8841	0.9687
all HP	12	6	1	1	0.7500	0.6452	0.8319	0.8855	0.8197	0.9294
all HP	12	6	1	2	0.7500	0.6452	0.8319	0.9160	0.8559	0.9525
all HP	12	6	1	3	0.7500	0.6452	0.8319	0.9237	0.8652	0.9580
all HP	12	6	1	4	0.7500	0.6452	0.8319	0.9237	0.8652	0.9580
all HP	12	6	1	5	0.7500	0.6452	0.8319	0.9237	0.8652	0.9580
all HP	12	6	1	6	0.7250	0.6186	0.8108	0.9389	0.8841	0.9687
all HP	12	6	2	1	0.7250	0.6186	0.8108	0.9084	0.8467	0.9468
all HP	12	6	2	2	0.7250	0.6186	0.8108	0.9237	0.8652	0.9580
all HP	12	6	2	3	0.7250	0.6186	0.8108	0.9313	0.8746	0.9634
all HP	12	6	2	4	0.7250	0.6186	0.8108	0.9313	0.8746	0.9634
all HP	12	6	2	5	0.7250	0.6186	0.8108	0.9313	0.8746	0.9634
all HP	12	6	2	6	0.7000	0.5923	0.7894	0.9466	0.8938	0.9739
all HP	12	6	3	1	0.7250	0.6186	0.8108	0.9313	0.8746	0.9634
all HP	12	6	3	2	0.7250	0.6186	0.8108	0.9389	0.8841	0.9687
all HP	12	6	3	3	0.7250	0.6186	0.8108	0.9389	0.8841	0.9687
all HP	12	6	3	4	0.7250	0.6186	0.8108	0.9389	0.8841	0.9687
all HP	12	6	3	5	0.7250	0.6186	0.8108	0.9389	0.8841	0.9687
all HP	12	6	3	6	0.7000	0.5923	0.7894	0.9466	0.8938	0.9739
all HP	12	6	4	1	0.7000	0.5923	0.7894	0.9313	0.8746	0.9634
all HP	12	6	4	2	0.7000	0.5923	0.7894	0.9389	0.8841	0.9687
all HP	12	6	4	3	0.7000	0.5923	0.7894	0.9389	0.8841	0.9687
all HP	12	6	4	4	0.7000	0.5923	0.7894	0.9389	0.8841	0.9687
all HP	12	6	4	5	0.7000	0.5923	0.7894	0.9389	0.8841	0.9687
all HP	12	6	4	6	0.6750	0.5664	0.7676	0.9466	0.8938	0.9739
all HP	12	6	5	1	0.7000	0.5923	0.7894	0.9313	0.8746	0.9634
all HP	12	6	5	2	0.7000	0.5923	0.7894	0.9389	0.8841	0.9687
all HP	12	6	5	3	0.7000	0.5923	0.7894	0.9389	0.8841	0.9687
all HP	12	6	5	4	0.7000	0.5923	0.7894	0.9389	0.8841	0.9687
all HP	12	6	5	5	0.7000	0.5923	0.7894	0.9389	0.8841	0.9687
all HP	12	6	5	6	0.6750	0.5664	0.7676	0.9466	0.8938	0.9739
all HP	12	6	6	1	0.6500	0.5408	0.7455	0.9389	0.8841	0.9687

		Threshold				95% CI			95% CI	
Combination	EK	A	B	C	Sensitivity	Lower	Upper	Specificity	Lower	Upper
all HP	12	6	6	2	0.6500	0.5408	0.7455	0.9466	0.8938	0.9739
all HP	12	6	6	3	0.6500	0.5408	0.7455	0.9466	0.8938	0.9739
all HP	12	6	6	4	0.6500	0.5408	0.7455	0.9466	0.8938	0.9739
all HP	12	6	6	5	0.6500	0.5408	0.7455	0.9466	0.8938	0.9739
all HP	12	6	6	6	0.6250	0.5155	0.7231	0.9542	0.9037	0.9788
(A OR B) AND C HP	13	1	1	1	0.9750	0.9134	0.9931	0.2901	0.2192	0.3729
(A OR B) AND C HP	13	1	1	2	0.9750	0.9134	0.9931	0.4046	0.3244	0.4902
(A OR B) AND C HP	13	1	1	3	0.9375	0.8619	0.9730	0.5038	0.4193	0.5881
(A OR B) AND C HP	13	1	1	4	0.9375	0.8619	0.9730	0.5496	0.4642	0.6322
(A OR B) AND C HP	13	1	1	5	0.9125	0.8302	0.9570	0.6107	0.5252	0.6899
(A OR B) AND C HP	13	1	1	6	0.8250	0.7274	0.8928	0.7176	0.6351	0.7876
(A OR B) AND C HP	13	1	2	1	0.9500	0.8784	0.9804	0.3053	0.2330	0.3888
(A OR B) AND C HP	13	1	2	2	0.9500	0.8784	0.9804	0.4198	0.3388	0.5055
(A OR B) AND C HP	13	1	2	3	0.9125	0.8302	0.9570	0.5115	0.4268	0.5955
(A OR B) AND C HP	13	1	2	4	0.9125	0.8302	0.9570	0.5573	0.4718	0.6395
(A OR B) AND C HP	13	1	2	5	0.9000	0.8149	0.9485	0.6183	0.5329	0.6970
(A OR B) AND C HP	13	1	2	6	0.8125	0.7134	0.8829	0.7252	0.6432	0.7944
(A OR B) AND C HP	13	1	3	1	0.9500	0.8784	0.9804	0.3282	0.2537	0.4125
(A OR B) AND C HP	13	1	3	2	0.9500	0.8784	0.9804	0.4427	0.3605	0.5282
(A OR B) AND C HP	13	1	3	3	0.9125	0.8302	0.9570	0.5267	0.4417	0.6102
(A OR B) AND C HP	13	1	3	4	0.9125	0.8302	0.9570	0.5725	0.4869	0.6540
(A OR B) AND C HP	13	1	3	5	0.9000	0.8149	0.9485	0.6336	0.5484	0.7112
(A OR B) AND C HP	13	1	3	6	0.8125	0.7134	0.8829	0.7328	0.6512	0.8012
(A OR B) AND C HP	13	1	4	1	0.9375	0.8619	0.9730	0.3435	0.2677	0.4282
(A OR B) AND C HP	13	1	4	2	0.9375	0.8619	0.9730	0.4580	0.3751	0.5433
(A OR B) AND C HP	13	1	4	3	0.9125	0.8302	0.9570	0.5267	0.4417	0.6102
(A OR B) AND C HP	13	1	4	4	0.9125	0.8302	0.9570	0.5725	0.4869	0.6540
(A OR B) AND C HP	13	1	4	5	0.9000	0.8149	0.9485	0.6336	0.5484	0.7112
(A OR B) AND C HP	13	1	4	6	0.8125	0.7134	0.8829	0.7328	0.6512	0.8012
(A OR B) AND C HP	13	1	5	1	0.9250	0.8459	0.9652	0.3435	0.2677	0.4282
(A OR B) AND C HP	13	1	5	2	0.9250	0.8459	0.9652	0.4580	0.3751	0.5433
(A OR B) AND C HP	13	1	5	3	0.9000	0.8149	0.9485	0.5267	0.4417	0.6102
(A OR B) AND C HP	13	1	5	4	0.9000	0.8149	0.9485	0.5725	0.4869	0.6540
(A OR B) AND C HP	13	1	5	5	0.8875	0.7998	0.9397	0.6336	0.5484	0.7112
(A OR B) AND C HP	13	1	5	6	0.8125	0.7134	0.8829	0.7328	0.6512	0.8012
(A OR B) AND C HP	13	1	6	1	0.9125	0.8302	0.9570	0.3511	0.2747	0.4361
(A OR B) AND C HP	13	1	6	2	0.9125	0.8302	0.9570	0.4656	0.3824	0.5508
(A OR B) AND C HP	13	1	6	3	0.8875	0.7998	0.9397	0.5344	0.4492	0.6176
(A OR B) AND C HP	13	1	6	4	0.8875	0.7998	0.9397	0.5802	0.4945	0.6612
(A OR B) AND C HP	13	1	6	5	0.8750	0.7850	0.9307	0.6336	0.5484	0.7112
(A OR B) AND C HP	13	1	6	6	0.8000	0.6995	0.8730	0.7328	0.6512	0.8012
(A OR B) AND C HP	13	2	1	1	0.9625	0.8955	0.9872	0.3206	0.2468	0.4047
(A OR B) AND C HP	13	2	1	2	0.9625	0.8955	0.9872	0.4275	0.3460	0.5131
(A OR B) AND C HP	13	2	1	3	0.9375	0.8619	0.9730	0.5267	0.4417	0.6102
(A OR B) AND C HP	13	2	1	4	0.9375	0.8619	0.9730	0.5725	0.4869	0.6540
(A OR B) AND C HP	13	2	1	5	0.9125	0.8302	0.9570	0.6260	0.5406	0.7041
(A OR B) AND C HP	13	2	1	6	0.8250	0.7274	0.8928	0.7328	0.6512	0.8012
(A OR B) AND C HP	13	2	2	1	0.9375	0.8619	0.9730	0.3511	0.2747	0.4361
(A OR B) AND C HP	13	2	2	2	0.9375	0.8619	0.9730	0.4580	0.3751	0.5433
(A OR B) AND C HP	13	2	2	3	0.9125	0.8302	0.9570	0.5496	0.4642	0.6322
(A OR B) AND C HP	13	2	2	4	0.9125	0.8302	0.9570	0.5954	0.5098	0.6756
(A OR B) AND C HP	13	2	2	5	0.9000	0.8149	0.9485	0.6412	0.5561	0.7183
(A OR B) AND C HP	13	2	2	6	0.8125	0.7134	0.8829	0.7405	0.6593	0.8079

		Threshold				95% CI			95% CI	
Combination	EK	A	B	C	Sensitivity	Lower	Upper	Specificity	Lower	Upper
(A OR B) AND C HP	13	2	3	1	0.9375	0.8619	0.9730	0.3740	0.2959	0.4594
(A OR B) AND C HP	13	2	3	2	0.9375	0.8619	0.9730	0.4809	0.3971	0.5658
(A OR B) AND C HP	13	2	3	3	0.9125	0.8302	0.9570	0.5649	0.4793	0.6467
(A OR B) AND C HP	13	2	3	4	0.9125	0.8302	0.9570	0.6107	0.5252	0.6899
(A OR B) AND C HP	13	2	3	5	0.9000	0.8149	0.9485	0.6565	0.5718	0.7323
(A OR B) AND C HP	13	2	3	6	0.8125	0.7134	0.8829	0.7481	0.6674	0.8146
(A OR B) AND C HP	13	2	4	1	0.9250	0.8459	0.9652	0.3969	0.3172	0.4825
(A OR B) AND C HP	13	2	4	2	0.9250	0.8459	0.9652	0.4962	0.4119	0.5807
(A OR B) AND C HP	13	2	4	3	0.9125	0.8302	0.9570	0.5649	0.4793	0.6467
(A OR B) AND C HP	13	2	4	4	0.9125	0.8302	0.9570	0.6107	0.5252	0.6899
(A OR B) AND C HP	13	2	4	5	0.9000	0.8149	0.9485	0.6565	0.5718	0.7323
(A OR B) AND C HP	13	2	4	6	0.8125	0.7134	0.8829	0.7481	0.6674	0.8146
(A OR B) AND C HP	13	2	5	1	0.9125	0.8302	0.9570	0.4046	0.3244	0.4902
(A OR B) AND C HP	13	2	5	2	0.9125	0.8302	0.9570	0.4962	0.4119	0.5807
(A OR B) AND C HP	13	2	5	3	0.9000	0.8149	0.9485	0.5649	0.4793	0.6467
(A OR B) AND C HP	13	2	5	4	0.9000	0.8149	0.9485	0.6107	0.5252	0.6899
(A OR B) AND C HP	13	2	5	5	0.8875	0.7998	0.9397	0.6565	0.5718	0.7323
(A OR B) AND C HP	13	2	5	6	0.8125	0.7134	0.8829	0.7481	0.6674	0.8146
(A OR B) AND C HP	13	2	6	1	0.9000	0.8149	0.9485	0.4122	0.3316	0.4978
(A OR B) AND C HP	13	2	6	2	0.9000	0.8149	0.9485	0.5038	0.4193	0.5881
(A OR B) AND C HP	13	2	6	3	0.8875	0.7998	0.9397	0.5725	0.4869	0.6540
(A OR B) AND C HP	13	2	6	4	0.8875	0.7998	0.9397	0.6183	0.5329	0.6970
(A OR B) AND C HP	13	2	6	5	0.8750	0.7850	0.9307	0.6565	0.5718	0.7323
(A OR B) AND C HP	13	2	6	6	0.8000	0.6995	0.8730	0.7481	0.6674	0.8146
(A OR B) AND C HP	13	3	1	1	0.9625	0.8955	0.9872	0.3435	0.2677	0.4282
(A OR B) AND C HP	13	3	1	2	0.9625	0.8955	0.9872	0.4504	0.3678	0.5358
(A OR B) AND C HP	13	3	1	3	0.9375	0.8619	0.9730	0.5420	0.4567	0.6249
(A OR B) AND C HP	13	3	1	4	0.9375	0.8619	0.9730	0.5802	0.4945	0.6612
(A OR B) AND C HP	13	3	1	5	0.9125	0.8302	0.9570	0.6336	0.5484	0.7112
(A OR B) AND C HP	13	3	1	6	0.8250	0.7274	0.8928	0.7405	0.6593	0.8079
(A OR B) AND C HP	13	3	2	1	0.9375	0.8619	0.9730	0.3969	0.3172	0.4825
(A OR B) AND C HP	13	3	2	2	0.9375	0.8619	0.9730	0.5038	0.4193	0.5881
(A OR B) AND C HP	13	3	2	3	0.9125	0.8302	0.9570	0.5878	0.5022	0.6684
(A OR B) AND C HP	13	3	2	4	0.9125	0.8302	0.9570	0.6260	0.5406	0.7041
(A OR B) AND C HP	13	3	2	5	0.9000	0.8149	0.9485	0.6718	0.5875	0.7463
(A OR B) AND C HP	13	3	2	6	0.8125	0.7134	0.8829	0.7634	0.6837	0.8280
(A OR B) AND C HP	13	3	3	1	0.9375	0.8619	0.9730	0.4427	0.3605	0.5282
(A OR B) AND C HP	13	3	3	2	0.9375	0.8619	0.9730	0.5496	0.4642	0.6322
(A OR B) AND C HP	13	3	3	3	0.9125	0.8302	0.9570	0.6260	0.5406	0.7041
(A OR B) AND C HP	13	3	3	4	0.9125	0.8302	0.9570	0.6565	0.5718	0.7323
(A OR B) AND C HP	13	3	3	5	0.9000	0.8149	0.9485	0.7023	0.6191	0.7739
(A OR B) AND C HP	13	3	3	6	0.8125	0.7134	0.8829	0.7863	0.7084	0.8478
(A OR B) AND C HP	13	3	4	1	0.9250	0.8459	0.9652	0.4885	0.4045	0.5732
(A OR B) AND C HP	13	3	4	2	0.9250	0.8459	0.9652	0.5802	0.4945	0.6612
(A OR B) AND C HP	13	3	4	3	0.9125	0.8302	0.9570	0.6412	0.5561	0.7183
(A OR B) AND C HP	13	3	4	4	0.9125	0.8302	0.9570	0.6718	0.5875	0.7463
(A OR B) AND C HP	13	3	4	5	0.9000	0.8149	0.9485	0.7023	0.6191	0.7739
(A OR B) AND C HP	13	3	4	6	0.8125	0.7134	0.8829	0.7863	0.7084	0.8478
(A OR B) AND C HP	13	3	5	1	0.9125	0.8302	0.9570	0.5115	0.4268	0.5955
(A OR B) AND C HP	13	3	5	2	0.9125	0.8302	0.9570	0.5954	0.5098	0.6756
(A OR B) AND C HP	13	3	5	3	0.9000	0.8149	0.9485	0.6565	0.5718	0.7323
(A OR B) AND C HP	13	3	5	4	0.9000	0.8149	0.9485	0.6870	0.6032	0.7601
(A OR B) AND C HP	13	3	5	5	0.8875	0.7998	0.9397	0.7176	0.6351	0.7876

		Threshold				95% CI			95% CI	
Combination	EK	A	B	C	Sensitivity	Lower	Upper	Specificity	Lower	Upper
(A OR B) AND C HP	13	3	5	6	0.8125	0.7134	0.8829	0.7939	0.7167	0.8543
(A OR B) AND C HP	13	3	6	1	0.9000	0.8149	0.9485	0.5191	0.4342	0.6029
(A OR B) AND C HP	13	3	6	2	0.9000	0.8149	0.9485	0.6031	0.5175	0.6828
(A OR B) AND C HP	13	3	6	3	0.8875	0.7998	0.9397	0.6641	0.5796	0.7393
(A OR B) AND C HP	13	3	6	4	0.8875	0.7998	0.9397	0.6947	0.6112	0.7670
(A OR B) AND C HP	13	3	6	5	0.8750	0.7850	0.9307	0.7176	0.6351	0.7876
(A OR B) AND C HP	13	3	6	6	0.8000	0.6995	0.8730	0.7939	0.7167	0.8543
(A OR B) AND C HP	13	4	1	1	0.9500	0.8784	0.9804	0.3969	0.3172	0.4825
(A OR B) AND C HP	13	4	1	2	0.9500	0.8784	0.9804	0.4809	0.3971	0.5658
(A OR B) AND C HP	13	4	1	3	0.9375	0.8619	0.9730	0.5573	0.4718	0.6395
(A OR B) AND C HP	13	4	1	4	0.9375	0.8619	0.9730	0.5954	0.5098	0.6756
(A OR B) AND C HP	13	4	1	5	0.9125	0.8302	0.9570	0.6489	0.5639	0.7253
(A OR B) AND C HP	13	4	1	6	0.8250	0.7274	0.8928	0.7557	0.6756	0.8213
(A OR B) AND C HP	13	4	2	1	0.9250	0.8459	0.9652	0.4580	0.3751	0.5433
(A OR B) AND C HP	13	4	2	2	0.9250	0.8459	0.9652	0.5420	0.4567	0.6249
(A OR B) AND C HP	13	4	2	3	0.9125	0.8302	0.9570	0.6107	0.5252	0.6899
(A OR B) AND C HP	13	4	2	4	0.9125	0.8302	0.9570	0.6412	0.5561	0.7183
(A OR B) AND C HP	13	4	2	5	0.9000	0.8149	0.9485	0.6870	0.6032	0.7601
(A OR B) AND C HP	13	4	2	6	0.8125	0.7134	0.8829	0.7786	0.7002	0.8412
(A OR B) AND C HP	13	4	3	1	0.9250	0.8459	0.9652	0.5191	0.4342	0.6029
(A OR B) AND C HP	13	4	3	2	0.9250	0.8459	0.9652	0.6031	0.5175	0.6828
(A OR B) AND C HP	13	4	3	3	0.9125	0.8302	0.9570	0.6641	0.5796	0.7393
(A OR B) AND C HP	13	4	3	4	0.9125	0.8302	0.9570	0.6870	0.6032	0.7601
(A OR B) AND C HP	13	4	3	5	0.9000	0.8149	0.9485	0.7328	0.6512	0.8012
(A OR B) AND C HP	13	4	3	6	0.8125	0.7134	0.8829	0.8168	0.7419	0.8737
(A OR B) AND C HP	13	4	4	1	0.8875	0.7998	0.9397	0.5954	0.5098	0.6756
(A OR B) AND C HP	13	4	4	2	0.8875	0.7998	0.9397	0.6641	0.5796	0.7393
(A OR B) AND C HP	13	4	4	3	0.8875	0.7998	0.9397	0.7099	0.6271	0.7808
(A OR B) AND C HP	13	4	4	4	0.8875	0.7998	0.9397	0.7252	0.6432	0.7944
(A OR B) AND C HP	13	4	4	5	0.8875	0.7998	0.9397	0.7557	0.6756	0.8213
(A OR B) AND C HP	13	4	4	6	0.8125	0.7134	0.8829	0.8321	0.7588	0.8864
(A OR B) AND C HP	13	4	5	1	0.8750	0.7850	0.9307	0.6183	0.5329	0.6970
(A OR B) AND C HP	13	4	5	2	0.8750	0.7850	0.9307	0.6794	0.5953	0.7532
(A OR B) AND C HP	13	4	5	3	0.8750	0.7850	0.9307	0.7252	0.6432	0.7944
(A OR B) AND C HP	13	4	5	4	0.8750	0.7850	0.9307	0.7405	0.6593	0.8079
(A OR B) AND C HP	13	4	5	5	0.8750	0.7850	0.9307	0.7710	0.6919	0.8346
(A OR B) AND C HP	13	4	5	6	0.8125	0.7134	0.8829	0.8397	0.7673	0.8927
(A OR B) AND C HP	13	4	6	1	0.8625	0.7703	0.9215	0.6260	0.5406	0.7041
(A OR B) AND C HP	13	4	6	2	0.8625	0.7703	0.9215	0.6870	0.6032	0.7601
(A OR B) AND C HP	13	4	6	3	0.8625	0.7703	0.9215	0.7328	0.6512	0.8012
(A OR B) AND C HP	13	4	6	4	0.8625	0.7703	0.9215	0.7481	0.6674	0.8146
(A OR B) AND C HP	13	4	6	5	0.8625	0.7703	0.9215	0.7710	0.6919	0.8346
(A OR B) AND C HP	13	4	6	6	0.8000	0.6995	0.8730	0.8397	0.7673	0.8927
(A OR B) AND C HP	13	5	1	1	0.9500	0.8784	0.9804	0.4122	0.3316	0.4978
(A OR B) AND C HP	13	5	1	2	0.9500	0.8784	0.9804	0.4962	0.4119	0.5807
(A OR B) AND C HP	13	5	1	3	0.9375	0.8619	0.9730	0.5725	0.4869	0.6540
(A OR B) AND C HP	13	5	1	4	0.9375	0.8619	0.9730	0.6107	0.5252	0.6899
(A OR B) AND C HP	13	5	1	5	0.9125	0.8302	0.9570	0.6641	0.5796	0.7393
(A OR B) AND C HP	13	5	1	6	0.8250	0.7274	0.8928	0.7710	0.6919	0.8346
(A OR B) AND C HP	13	5	2	1	0.9250	0.8459	0.9652	0.4885	0.4045	0.5732
(A OR B) AND C HP	13	5	2	2	0.9250	0.8459	0.9652	0.5725	0.4869	0.6540
(A OR B) AND C HP	13	5	2	3	0.9125	0.8302	0.9570	0.6412	0.5561	0.7183
(A OR B) AND C HP	13	5	2	4	0.9125	0.8302	0.9570	0.6718	0.5875	0.7463

		Threshold				95% CI			95% CI	
Combination	EK	A	B	C	Sensitivity	Lower	Upper	Specificity	Lower	Upper
(A OR B) AND C HP	13	5	2	5	0.9000	0.8149	0.9485	0.7176	0.6351	0.7876
(A OR B) AND C HP	13	5	2	6	0.8125	0.7134	0.8829	0.8015	0.7251	0.8608
(A OR B) AND C HP	13	5	3	1	0.9250	0.8459	0.9652	0.5725	0.4869	0.6540
(A OR B) AND C HP	13	5	3	2	0.9250	0.8459	0.9652	0.6565	0.5718	0.7323
(A OR B) AND C HP	13	5	3	3	0.9125	0.8302	0.9570	0.7176	0.6351	0.7876
(A OR B) AND C HP	13	5	3	4	0.9125	0.8302	0.9570	0.7405	0.6593	0.8079
(A OR B) AND C HP	13	5	3	5	0.9000	0.8149	0.9485	0.7863	0.7084	0.8478
(A OR B) AND C HP	13	5	3	6	0.8125	0.7134	0.8829	0.8550	0.7846	0.9051
(A OR B) AND C HP	13	5	4	1	0.8750	0.7850	0.9307	0.6565	0.5718	0.7323
(A OR B) AND C HP	13	5	4	2	0.8750	0.7850	0.9307	0.7252	0.6432	0.7944
(A OR B) AND C HP	13	5	4	3	0.8750	0.7850	0.9307	0.7710	0.6919	0.8346
(A OR B) AND C HP	13	5	4	4	0.8750	0.7850	0.9307	0.7863	0.7084	0.8478
(A OR B) AND C HP	13	5	4	5	0.8750	0.7850	0.9307	0.8168	0.7419	0.8737
(A OR B) AND C HP	13	5	4	6	0.8000	0.6995	0.8730	0.8702	0.8020	0.9174
(A OR B) AND C HP	13	5	5	1	0.8625	0.7703	0.9215	0.6794	0.5953	0.7532
(A OR B) AND C HP	13	5	5	2	0.8625	0.7703	0.9215	0.7405	0.6593	0.8079
(A OR B) AND C HP	13	5	5	3	0.8625	0.7703	0.9215	0.7863	0.7084	0.8478
(A OR B) AND C HP	13	5	5	4	0.8625	0.7703	0.9215	0.8015	0.7251	0.8608
(A OR B) AND C HP	13	5	5	5	0.8625	0.7703	0.9215	0.8321	0.7588	0.8864
(A OR B) AND C HP	13	5	5	6	0.8000	0.6995	0.8730	0.8779	0.8108	0.9234
(A OR B) AND C HP	13	5	6	1	0.8375	0.7416	0.9025	0.6870	0.6032	0.7601
(A OR B) AND C HP	13	5	6	2	0.8375	0.7416	0.9025	0.7481	0.6674	0.8146
(A OR B) AND C HP	13	5	6	3	0.8375	0.7416	0.9025	0.7939	0.7167	0.8543
(A OR B) AND C HP	13	5	6	4	0.8375	0.7416	0.9025	0.8092	0.7334	0.8673
(A OR B) AND C HP	13	5	6	5	0.8375	0.7416	0.9025	0.8321	0.7588	0.8864
(A OR B) AND C HP	13	5	6	6	0.7875	0.6858	0.8629	0.8779	0.8108	0.9234
(A OR B) AND C HP	13	6	1	1	0.9500	0.8784	0.9804	0.4427	0.3605	0.5282
(A OR B) AND C HP	13	6	1	2	0.9500	0.8784	0.9804	0.5267	0.4417	0.6102
(A OR B) AND C HP	13	6	1	3	0.9375	0.8619	0.9730	0.5954	0.5098	0.6756
(A OR B) AND C HP	13	6	1	4	0.9375	0.8619	0.9730	0.6183	0.5329	0.6970
(A OR B) AND C HP	13	6	1	5	0.9125	0.8302	0.9570	0.6718	0.5875	0.7463
(A OR B) AND C HP	13	6	1	6	0.8250	0.7274	0.8928	0.7786	0.7002	0.8412
(A OR B) AND C HP	13	6	2	1	0.9250	0.8459	0.9652	0.5191	0.4342	0.6029
(A OR B) AND C HP	13	6	2	2	0.9250	0.8459	0.9652	0.6031	0.5175	0.6828
(A OR B) AND C HP	13	6	2	3	0.9125	0.8302	0.9570	0.6641	0.5796	0.7393
(A OR B) AND C HP	13	6	2	4	0.9125	0.8302	0.9570	0.6794	0.5953	0.7532
(A OR B) AND C HP	13	6	2	5	0.9000	0.8149	0.9485	0.7252	0.6432	0.7944
(A OR B) AND C HP	13	6	2	6	0.8125	0.7134	0.8829	0.8092	0.7334	0.8673
(A OR B) AND C HP	13	6	3	1	0.9250	0.8459	0.9652	0.6183	0.5329	0.6970
(A OR B) AND C HP	13	6	3	2	0.9250	0.8459	0.9652	0.7023	0.6191	0.7739
(A OR B) AND C HP	13	6	3	3	0.9125	0.8302	0.9570	0.7557	0.6756	0.8213
(A OR B) AND C HP	13	6	3	4	0.9125	0.8302	0.9570	0.7634	0.6837	0.8280
(A OR B) AND C HP	13	6	3	5	0.9000	0.8149	0.9485	0.8015	0.7251	0.8608
(A OR B) AND C HP	13	6	3	6	0.8125	0.7134	0.8829	0.8702	0.8020	0.9174
(A OR B) AND C HP	13	6	4	1	0.8750	0.7850	0.9307	0.7023	0.6191	0.7739
(A OR B) AND C HP	13	6	4	2	0.8750	0.7850	0.9307	0.7710	0.6919	0.8346
(A OR B) AND C HP	13	6	4	3	0.8750	0.7850	0.9307	0.8092	0.7334	0.8673
(A OR B) AND C HP	13	6	4	4	0.8750	0.7850	0.9307	0.8092	0.7334	0.8673
(A OR B) AND C HP	13	6	4	5	0.8750	0.7850	0.9307	0.8321	0.7588	0.8864
(A OR B) AND C HP	13	6	4	6	0.8000	0.6995	0.8730	0.8855	0.8197	0.9294
(A OR B) AND C HP	13	6	5	1	0.8625	0.7703	0.9215	0.7252	0.6432	0.7944
(A OR B) AND C HP	13	6	5	2	0.8625	0.7703	0.9215	0.7863	0.7084	0.8478
(A OR B) AND C HP	13	6	5	3	0.8625	0.7703	0.9215	0.8244	0.7503	0.8801

		Threshold				95% CI			95% CI	
Combination	EK	A	B	C	Sensitivity	Lower	Upper	Specificity	Lower	Upper
(A OR B) AND C HP	13	6	5	4	0.8625	0.7703	0.9215	0.8244	0.7503	0.8801
(A OR B) AND C HP	13	6	5	5	0.8625	0.7703	0.9215	0.8473	0.7759	0.8989
(A OR B) AND C HP	13	6	5	6	0.8000	0.6995	0.8730	0.8931	0.8286	0.9353
(A OR B) AND C HP	13	6	6	1	0.8375	0.7416	0.9025	0.7405	0.6593	0.8079
(A OR B) AND C HP	13	6	6	2	0.8375	0.7416	0.9025	0.8015	0.7251	0.8608
(A OR B) AND C HP	13	6	6	3	0.8375	0.7416	0.9025	0.8397	0.7673	0.8927
(A OR B) AND C HP	13	6	6	4	0.8375	0.7416	0.9025	0.8397	0.7673	0.8927
(A OR B) AND C HP	13	6	6	5	0.8375	0.7416	0.9025	0.8473	0.7759	0.8989
(A OR B) AND C HP	13	6	6	6	0.7875	0.6858	0.8629	0.8931	0.8286	0.9353
(B OR C) AND A HP	14	1	1	1	0.9125	0.8302	0.9570	0.2977	0.2261	0.3809
(B OR C) AND A HP	14	1	1	2	0.9125	0.8302	0.9570	0.3511	0.2747	0.4361
(B OR C) AND A HP	14	1	1	3	0.8875	0.7998	0.9397	0.4046	0.3244	0.4902
(B OR C) AND A HP	14	1	1	4	0.8875	0.7998	0.9397	0.4275	0.3460	0.5131
(B OR C) AND A HP	14	1	1	5	0.8875	0.7998	0.9397	0.4427	0.3605	0.5282
(B OR C) AND A HP	14	1	1	6	0.8875	0.7998	0.9397	0.4580	0.3751	0.5433
(B OR C) AND A HP	14	1	2	1	0.9125	0.8302	0.9570	0.3053	0.2330	0.3888
(B OR C) AND A HP	14	1	2	2	0.9125	0.8302	0.9570	0.3740	0.2959	0.4594
(B OR C) AND A HP	14	1	2	3	0.8875	0.7998	0.9397	0.4275	0.3460	0.5131
(B OR C) AND A HP	14	1	2	4	0.8875	0.7998	0.9397	0.4580	0.3751	0.5433
(B OR C) AND A HP	14	1	2	5	0.8875	0.7998	0.9397	0.4809	0.3971	0.5658
(B OR C) AND A HP	14	1	2	6	0.8875	0.7998	0.9397	0.5191	0.4342	0.6029
(B OR C) AND A HP	14	1	3	1	0.9125	0.8302	0.9570	0.3206	0.2468	0.4047
(B OR C) AND A HP	14	1	3	2	0.9125	0.8302	0.9570	0.3969	0.3172	0.4825
(B OR C) AND A HP	14	1	3	3	0.8875	0.7998	0.9397	0.4580	0.3751	0.5433
(B OR C) AND A HP	14	1	3	4	0.8875	0.7998	0.9397	0.4962	0.4119	0.5807
(B OR C) AND A HP	14	1	3	5	0.8875	0.7998	0.9397	0.5267	0.4417	0.6102
(B OR C) AND A HP	14	1	3	6	0.8875	0.7998	0.9397	0.5802	0.4945	0.6612
(B OR C) AND A HP	14	1	4	1	0.9125	0.8302	0.9570	0.3511	0.2747	0.4361
(B OR C) AND A HP	14	1	4	2	0.9125	0.8302	0.9570	0.4427	0.3605	0.5282
(B OR C) AND A HP	14	1	4	3	0.8875	0.7998	0.9397	0.5038	0.4193	0.5881
(B OR C) AND A HP	14	1	4	4	0.8875	0.7998	0.9397	0.5496	0.4642	0.6322
(B OR C) AND A HP	14	1	4	5	0.8750	0.7850	0.9307	0.5954	0.5098	0.6756
(B OR C) AND A HP	14	1	4	6	0.8625	0.7703	0.9215	0.6641	0.5796	0.7393
(B OR C) AND A HP	14	1	5	1	0.9125	0.8302	0.9570	0.3588	0.2817	0.4439
(B OR C) AND A HP	14	1	5	2	0.9125	0.8302	0.9570	0.4580	0.3751	0.5433
(B OR C) AND A HP	14	1	5	3	0.8875	0.7998	0.9397	0.5191	0.4342	0.6029
(B OR C) AND A HP	14	1	5	4	0.8875	0.7998	0.9397	0.5649	0.4793	0.6467
(B OR C) AND A HP	14	1	5	5	0.8750	0.7850	0.9307	0.6107	0.5252	0.6899
(B OR C) AND A HP	14	1	5	6	0.8625	0.7703	0.9215	0.6870	0.6032	0.7601
(B OR C) AND A HP	14	1	6	1	0.9125	0.8302	0.9570	0.3664	0.2888	0.4516
(B OR C) AND A HP	14	1	6	2	0.9125	0.8302	0.9570	0.4656	0.3824	0.5508
(B OR C) AND A HP	14	1	6	3	0.8875	0.7998	0.9397	0.5267	0.4417	0.6102
(B OR C) AND A HP	14	1	6	4	0.8875	0.7998	0.9397	0.5725	0.4869	0.6540
(B OR C) AND A HP	14	1	6	5	0.8750	0.7850	0.9307	0.6260	0.5406	0.7041
(B OR C) AND A HP	14	1	6	6	0.8500	0.7559	0.9121	0.7023	0.6191	0.7739
(B OR C) AND A HP	14	2	1	1	0.8875	0.7998	0.9397	0.3740	0.2959	0.4594
(B OR C) AND A HP	14	2	1	2	0.8875	0.7998	0.9397	0.4198	0.3388	0.5055
(B OR C) AND A HP	14	2	1	3	0.8750	0.7850	0.9307	0.4733	0.3898	0.5583
(B OR C) AND A HP	14	2	1	4	0.8750	0.7850	0.9307	0.4962	0.4119	0.5807
(B OR C) AND A HP	14	2	1	5	0.8750	0.7850	0.9307	0.5038	0.4193	0.5881
(B OR C) AND A HP	14	2	1	6	0.8750	0.7850	0.9307	0.5191	0.4342	0.6029
(B OR C) AND A HP	14	2	2	1	0.8875	0.7998	0.9397	0.3817	0.3030	0.4671
(B OR C) AND A HP	14	2	2	2	0.8875	0.7998	0.9397	0.4427	0.3605	0.5282

		Threshold				95% CI			95% CI	
Combination	EK	A	B	C	Sensitivity	Lower	Upper	Specificity	Lower	Upper
(B OR C) AND A HP	14	2	2	3	0.8750	0.7850	0.9307	0.4962	0.4119	0.5807
(B OR C) AND A HP	14	2	2	4	0.8750	0.7850	0.9307	0.5267	0.4417	0.6102
(B OR C) AND A HP	14	2	2	5	0.8750	0.7850	0.9307	0.5344	0.4492	0.6176
(B OR C) AND A HP	14	2	2	6	0.8750	0.7850	0.9307	0.5649	0.4793	0.6467
(B OR C) AND A HP	14	2	3	1	0.8875	0.7998	0.9397	0.3893	0.3101	0.4748
(B OR C) AND A HP	14	2	3	2	0.8875	0.7998	0.9397	0.4580	0.3751	0.5433
(B OR C) AND A HP	14	2	3	3	0.8750	0.7850	0.9307	0.5191	0.4342	0.6029
(B OR C) AND A HP	14	2	3	4	0.8750	0.7850	0.9307	0.5573	0.4718	0.6395
(B OR C) AND A HP	14	2	3	5	0.8750	0.7850	0.9307	0.5725	0.4869	0.6540
(B OR C) AND A HP	14	2	3	6	0.8750	0.7850	0.9307	0.6183	0.5329	0.6970
(B OR C) AND A HP	14	2	4	1	0.8875	0.7998	0.9397	0.4198	0.3388	0.5055
(B OR C) AND A HP	14	2	4	2	0.8875	0.7998	0.9397	0.4962	0.4119	0.5807
(B OR C) AND A HP	14	2	4	3	0.8750	0.7850	0.9307	0.5573	0.4718	0.6395
(B OR C) AND A HP	14	2	4	4	0.8750	0.7850	0.9307	0.6031	0.5175	0.6828
(B OR C) AND A HP	14	2	4	5	0.8625	0.7703	0.9215	0.6336	0.5484	0.7112
(B OR C) AND A HP	14	2	4	6	0.8500	0.7559	0.9121	0.6947	0.6112	0.7670
(B OR C) AND A HP	14	2	5	1	0.8875	0.7998	0.9397	0.4198	0.3388	0.5055
(B OR C) AND A HP	14	2	5	2	0.8875	0.7998	0.9397	0.4962	0.4119	0.5807
(B OR C) AND A HP	14	2	5	3	0.8750	0.7850	0.9307	0.5573	0.4718	0.6395
(B OR C) AND A HP	14	2	5	4	0.8750	0.7850	0.9307	0.6031	0.5175	0.6828
(B OR C) AND A HP	14	2	5	5	0.8625	0.7703	0.9215	0.6336	0.5484	0.7112
(B OR C) AND A HP	14	2	5	6	0.8500	0.7559	0.9121	0.7023	0.6191	0.7739
(B OR C) AND A HP	14	2	6	1	0.8875	0.7998	0.9397	0.4275	0.3460	0.5131
(B OR C) AND A HP	14	2	6	2	0.8875	0.7998	0.9397	0.5038	0.4193	0.5881
(B OR C) AND A HP	14	2	6	3	0.8750	0.7850	0.9307	0.5649	0.4793	0.6467
(B OR C) AND A HP	14	2	6	4	0.8750	0.7850	0.9307	0.6107	0.5252	0.6899
(B OR C) AND A HP	14	2	6	5	0.8625	0.7703	0.9215	0.6489	0.5639	0.7253
(B OR C) AND A HP	14	2	6	6	0.8375	0.7416	0.9025	0.7176	0.6351	0.7876
(B OR C) AND A HP	14	3	1	1	0.8750	0.7850	0.9307	0.4962	0.4119	0.5807
(B OR C) AND A HP	14	3	1	2	0.8750	0.7850	0.9307	0.5420	0.4567	0.6249
(B OR C) AND A HP	14	3	1	3	0.8625	0.7703	0.9215	0.5878	0.5022	0.6684
(B OR C) AND A HP	14	3	1	4	0.8625	0.7703	0.9215	0.6031	0.5175	0.6828
(B OR C) AND A HP	14	3	1	5	0.8625	0.7703	0.9215	0.6107	0.5252	0.6899
(B OR C) AND A HP	14	3	1	6	0.8625	0.7703	0.9215	0.6260	0.5406	0.7041
(B OR C) AND A HP	14	3	2	1	0.8750	0.7850	0.9307	0.5038	0.4193	0.5881
(B OR C) AND A HP	14	3	2	2	0.8750	0.7850	0.9307	0.5649	0.4793	0.6467
(B OR C) AND A HP	14	3	2	3	0.8625	0.7703	0.9215	0.6107	0.5252	0.6899
(B OR C) AND A HP	14	3	2	4	0.8625	0.7703	0.9215	0.6336	0.5484	0.7112
(B OR C) AND A HP	14	3	2	5	0.8625	0.7703	0.9215	0.6412	0.5561	0.7183
(B OR C) AND A HP	14	3	2	6	0.8625	0.7703	0.9215	0.6641	0.5796	0.7393
(B OR C) AND A HP	14	3	3	1	0.8750	0.7850	0.9307	0.5115	0.4268	0.5955
(B OR C) AND A HP	14	3	3	2	0.8750	0.7850	0.9307	0.5802	0.4945	0.6612
(B OR C) AND A HP	14	3	3	3	0.8625	0.7703	0.9215	0.6336	0.5484	0.7112
(B OR C) AND A HP	14	3	3	4	0.8625	0.7703	0.9215	0.6565	0.5718	0.7323
(B OR C) AND A HP	14	3	3	5	0.8625	0.7703	0.9215	0.6718	0.5875	0.7463
(B OR C) AND A HP	14	3	3	6	0.8625	0.7703	0.9215	0.7099	0.6271	0.7808
(B OR C) AND A HP	14	3	4	1	0.8750	0.7850	0.9307	0.5344	0.4492	0.6176
(B OR C) AND A HP	14	3	4	2	0.8750	0.7850	0.9307	0.6031	0.5175	0.6828
(B OR C) AND A HP	14	3	4	3	0.8625	0.7703	0.9215	0.6565	0.5718	0.7323
(B OR C) AND A HP	14	3	4	4	0.8625	0.7703	0.9215	0.6870	0.6032	0.7601
(B OR C) AND A HP	14	3	4	5	0.8500	0.7559	0.9121	0.7023	0.6191	0.7739
(B OR C) AND A HP	14	3	4	6	0.8375	0.7416	0.9025	0.7557	0.6756	0.8213
(B OR C) AND A HP	14	3	5	1	0.8750	0.7850	0.9307	0.5344	0.4492	0.6176

		Threshold				95% CI			95% CI	
Combination	EK	A	B	C	Sensitivity	Lower	Upper	Specificity	Lower	Upper
(B OR C) AND A HP	14	3	5	2	0.8750	0.7850	0.9307	0.6031	0.5175	0.6828
(B OR C) AND A HP	14	3	5	3	0.8625	0.7703	0.9215	0.6565	0.5718	0.7323
(B OR C) AND A HP	14	3	5	4	0.8625	0.7703	0.9215	0.6870	0.6032	0.7601
(B OR C) AND A HP	14	3	5	5	0.8500	0.7559	0.9121	0.7023	0.6191	0.7739
(B OR C) AND A HP	14	3	5	6	0.8375	0.7416	0.9025	0.7557	0.6756	0.8213
(B OR C) AND A HP	14	3	6	1	0.8750	0.7850	0.9307	0.5344	0.4492	0.6176
(B OR C) AND A HP	14	3	6	2	0.8750	0.7850	0.9307	0.6031	0.5175	0.6828
(B OR C) AND A HP	14	3	6	3	0.8625	0.7703	0.9215	0.6565	0.5718	0.7323
(B OR C) AND A HP	14	3	6	4	0.8625	0.7703	0.9215	0.6870	0.6032	0.7601
(B OR C) AND A HP	14	3	6	5	0.8500	0.7559	0.9121	0.7099	0.6271	0.7808
(B OR C) AND A HP	14	3	6	6	0.8250	0.7274	0.8928	0.7634	0.6837	0.8280
(B OR C) AND A HP	14	4	1	1	0.8250	0.7274	0.8928	0.6260	0.5406	0.7041
(B OR C) AND A HP	14	4	1	2	0.8250	0.7274	0.8928	0.6489	0.5639	0.7253
(B OR C) AND A HP	14	4	1	3	0.8250	0.7274	0.8928	0.6794	0.5953	0.7532
(B OR C) AND A HP	14	4	1	4	0.8250	0.7274	0.8928	0.6947	0.6112	0.7670
(B OR C) AND A HP	14	4	1	5	0.8250	0.7274	0.8928	0.7023	0.6191	0.7739
(B OR C) AND A HP	14	4	1	6	0.8250	0.7274	0.8928	0.7176	0.6351	0.7876
(B OR C) AND A HP	14	4	2	1	0.8250	0.7274	0.8928	0.6336	0.5484	0.7112
(B OR C) AND A HP	14	4	2	2	0.8250	0.7274	0.8928	0.6718	0.5875	0.7463
(B OR C) AND A HP	14	4	2	3	0.8250	0.7274	0.8928	0.7023	0.6191	0.7739
(B OR C) AND A HP	14	4	2	4	0.8250	0.7274	0.8928	0.7176	0.6351	0.7876
(B OR C) AND A HP	14	4	2	5	0.8250	0.7274	0.8928	0.7252	0.6432	0.7944
(B OR C) AND A HP	14	4	2	6	0.8250	0.7274	0.8928	0.7481	0.6674	0.8146
(B OR C) AND A HP	14	4	3	1	0.8250	0.7274	0.8928	0.6412	0.5561	0.7183
(B OR C) AND A HP	14	4	3	2	0.8250	0.7274	0.8928	0.6870	0.6032	0.7601
(B OR C) AND A HP	14	4	3	3	0.8250	0.7274	0.8928	0.7252	0.6432	0.7944
(B OR C) AND A HP	14	4	3	4	0.8250	0.7274	0.8928	0.7405	0.6593	0.8079
(B OR C) AND A HP	14	4	3	5	0.8250	0.7274	0.8928	0.7557	0.6756	0.8213
(B OR C) AND A HP	14	4	3	6	0.8250	0.7274	0.8928	0.7939	0.7167	0.8543
(B OR C) AND A HP	14	4	4	1	0.8250	0.7274	0.8928	0.6489	0.5639	0.7253
(B OR C) AND A HP	14	4	4	2	0.8250	0.7274	0.8928	0.6947	0.6112	0.7670
(B OR C) AND A HP	14	4	4	3	0.8250	0.7274	0.8928	0.7328	0.6512	0.8012
(B OR C) AND A HP	14	4	4	4	0.8250	0.7274	0.8928	0.7481	0.6674	0.8146
(B OR C) AND A HP	14	4	4	5	0.8250	0.7274	0.8928	0.7634	0.6837	0.8280
(B OR C) AND A HP	14	4	4	6	0.8250	0.7274	0.8928	0.8092	0.7334	0.8673
(B OR C) AND A HP	14	4	5	1	0.8250	0.7274	0.8928	0.6489	0.5639	0.7253
(B OR C) AND A HP	14	4	5	2	0.8250	0.7274	0.8928	0.6947	0.6112	0.7670
(B OR C) AND A HP	14	4	5	3	0.8250	0.7274	0.8928	0.7328	0.6512	0.8012
(B OR C) AND A HP	14	4	5	4	0.8250	0.7274	0.8928	0.7481	0.6674	0.8146
(B OR C) AND A HP	14	4	5	5	0.8250	0.7274	0.8928	0.7634	0.6837	0.8280
(B OR C) AND A HP	14	4	5	6	0.8250	0.7274	0.8928	0.8092	0.7334	0.8673
(B OR C) AND A HP	14	4	6	1	0.8250	0.7274	0.8928	0.6489	0.5639	0.7253
(B OR C) AND A HP	14	4	6	2	0.8250	0.7274	0.8928	0.6947	0.6112	0.7670
(B OR C) AND A HP	14	4	6	3	0.8250	0.7274	0.8928	0.7328	0.6512	0.8012
(B OR C) AND A HP	14	4	6	4	0.8250	0.7274	0.8928	0.7481	0.6674	0.8146
(B OR C) AND A HP	14	4	6	5	0.8250	0.7274	0.8928	0.7710	0.6919	0.8346
(B OR C) AND A HP	14	4	6	6	0.8125	0.7134	0.8829	0.8168	0.7419	0.8737
(B OR C) AND A HP	14	5	1	1	0.8000	0.6995	0.8730	0.7023	0.6191	0.7739
(B OR C) AND A HP	14	5	1	2	0.8000	0.6995	0.8730	0.7252	0.6432	0.7944
(B OR C) AND A HP	14	5	1	3	0.8000	0.6995	0.8730	0.7557	0.6756	0.8213
(B OR C) AND A HP	14	5	1	4	0.8000	0.6995	0.8730	0.7710	0.6919	0.8346
(B OR C) AND A HP	14	5	1	5	0.8000	0.6995	0.8730	0.7786	0.7002	0.8412
(B OR C) AND A HP	14	5	1	6	0.8000	0.6995	0.8730	0.7939	0.7167	0.8543

		Threshold				95% CI			95% CI	
Combination	EK	A	B	C	Sensitivity	Lower	Upper	Specificity	Lower	Upper
(B OR C) AND A HP	14	5	2	1	0.8000	0.6995	0.8730	0.7099	0.6271	0.7808
(B OR C) AND A HP	14	5	2	2	0.8000	0.6995	0.8730	0.7481	0.6674	0.8146
(B OR C) AND A HP	14	5	2	3	0.8000	0.6995	0.8730	0.7786	0.7002	0.8412
(B OR C) AND A HP	14	5	2	4	0.8000	0.6995	0.8730	0.7939	0.7167	0.8543
(B OR C) AND A HP	14	5	2	5	0.8000	0.6995	0.8730	0.8015	0.7251	0.8608
(B OR C) AND A HP	14	5	2	6	0.8000	0.6995	0.8730	0.8168	0.7419	0.8737
(B OR C) AND A HP	14	5	3	1	0.8000	0.6995	0.8730	0.7099	0.6271	0.7808
(B OR C) AND A HP	14	5	3	2	0.8000	0.6995	0.8730	0.7557	0.6756	0.8213
(B OR C) AND A HP	14	5	3	3	0.8000	0.6995	0.8730	0.7939	0.7167	0.8543
(B OR C) AND A HP	14	5	3	4	0.8000	0.6995	0.8730	0.8092	0.7334	0.8673
(B OR C) AND A HP	14	5	3	5	0.8000	0.6995	0.8730	0.8244	0.7503	0.8801
(B OR C) AND A HP	14	5	3	6	0.8000	0.6995	0.8730	0.8473	0.7759	0.8989
(B OR C) AND A HP	14	5	4	1	0.8000	0.6995	0.8730	0.7176	0.6351	0.7876
(B OR C) AND A HP	14	5	4	2	0.8000	0.6995	0.8730	0.7634	0.6837	0.8280
(B OR C) AND A HP	14	5	4	3	0.8000	0.6995	0.8730	0.8015	0.7251	0.8608
(B OR C) AND A HP	14	5	4	4	0.8000	0.6995	0.8730	0.8168	0.7419	0.8737
(B OR C) AND A HP	14	5	4	5	0.8000	0.6995	0.8730	0.8321	0.7588	0.8864
(B OR C) AND A HP	14	5	4	6	0.8000	0.6995	0.8730	0.8550	0.7846	0.9051
(B OR C) AND A HP	14	5	5	1	0.8000	0.6995	0.8730	0.7176	0.6351	0.7876
(B OR C) AND A HP	14	5	5	2	0.8000	0.6995	0.8730	0.7634	0.6837	0.8280
(B OR C) AND A HP	14	5	5	3	0.8000	0.6995	0.8730	0.8015	0.7251	0.8608
(B OR C) AND A HP	14	5	5	4	0.8000	0.6995	0.8730	0.8168	0.7419	0.8737
(B OR C) AND A HP	14	5	5	5	0.8000	0.6995	0.8730	0.8321	0.7588	0.8864
(B OR C) AND A HP	14	5	5	6	0.8000	0.6995	0.8730	0.8550	0.7846	0.9051
(B OR C) AND A HP	14	5	6	1	0.8000	0.6995	0.8730	0.7176	0.6351	0.7876
(B OR C) AND A HP	14	5	6	2	0.8000	0.6995	0.8730	0.7634	0.6837	0.8280
(B OR C) AND A HP	14	5	6	3	0.8000	0.6995	0.8730	0.8015	0.7251	0.8608
(B OR C) AND A HP	14	5	6	4	0.8000	0.6995	0.8730	0.8168	0.7419	0.8737
(B OR C) AND A HP	14	5	6	5	0.8000	0.6995	0.8730	0.8397	0.7673	0.8927
(B OR C) AND A HP	14	5	6	6	0.8000	0.6995	0.8730	0.8626	0.7932	0.9113
(B OR C) AND A HP	14	6	1	1	0.7625	0.6586	0.8424	0.7863	0.7084	0.8478
(B OR C) AND A HP	14	6	1	2	0.7625	0.6586	0.8424	0.8092	0.7334	0.8673
(B OR C) AND A HP	14	6	1	3	0.7625	0.6586	0.8424	0.8321	0.7588	0.8864
(B OR C) AND A HP	14	6	1	4	0.7625	0.6586	0.8424	0.8321	0.7588	0.8864
(B OR C) AND A HP	14	6	1	5	0.7625	0.6586	0.8424	0.8397	0.7673	0.8927
(B OR C) AND A HP	14	6	1	6	0.7625	0.6586	0.8424	0.8550	0.7846	0.9051
(B OR C) AND A HP	14	6	2	1	0.7625	0.6586	0.8424	0.7939	0.7167	0.8543
(B OR C) AND A HP	14	6	2	2	0.7625	0.6586	0.8424	0.8321	0.7588	0.8864
(B OR C) AND A HP	14	6	2	3	0.7625	0.6586	0.8424	0.8550	0.7846	0.9051
(B OR C) AND A HP	14	6	2	4	0.7625	0.6586	0.8424	0.8550	0.7846	0.9051
(B OR C) AND A HP	14	6	2	5	0.7625	0.6586	0.8424	0.8626	0.7932	0.9113
(B OR C) AND A HP	14	6	2	6	0.7625	0.6586	0.8424	0.8779	0.8108	0.9234
(B OR C) AND A HP	14	6	3	1	0.7625	0.6586	0.8424	0.7939	0.7167	0.8543
(B OR C) AND A HP	14	6	3	2	0.7625	0.6586	0.8424	0.8397	0.7673	0.8927
(B OR C) AND A HP	14	6	3	3	0.7625	0.6586	0.8424	0.8702	0.8020	0.9174
(B OR C) AND A HP	14	6	3	4	0.7625	0.6586	0.8424	0.8702	0.8020	0.9174
(B OR C) AND A HP	14	6	3	5	0.7625	0.6586	0.8424	0.8779	0.8108	0.9234
(B OR C) AND A HP	14	6	3	6	0.7625	0.6586	0.8424	0.9008	0.8376	0.9411
(B OR C) AND A HP	14	6	4	1	0.7625	0.6586	0.8424	0.8015	0.7251	0.8608
(B OR C) AND A HP	14	6	4	2	0.7625	0.6586	0.8424	0.8473	0.7759	0.8989
(B OR C) AND A HP	14	6	4	3	0.7625	0.6586	0.8424	0.8779	0.8108	0.9234
(B OR C) AND A HP	14	6	4	4	0.7625	0.6586	0.8424	0.8779	0.8108	0.9234
(B OR C) AND A HP	14	6	4	5	0.7625	0.6586	0.8424	0.8855	0.8197	0.9294

		Threshold				95% CI			95% CI	
Combination	EK	A	B	C	Sensitivity	Lower	Upper	Specificity	Lower	Upper
(B OR C) AND A HP	14	6	4	6	0.7625	0.6586	0.8424	0.9084	0.8467	0.9468
(B OR C) AND A HP	14	6	5	1	0.7625	0.6586	0.8424	0.8015	0.7251	0.8608
(B OR C) AND A HP	14	6	5	2	0.7625	0.6586	0.8424	0.8473	0.7759	0.8989
(B OR C) AND A HP	14	6	5	3	0.7625	0.6586	0.8424	0.8779	0.8108	0.9234
(B OR C) AND A HP	14	6	5	4	0.7625	0.6586	0.8424	0.8779	0.8108	0.9234
(B OR C) AND A HP	14	6	5	5	0.7625	0.6586	0.8424	0.8855	0.8197	0.9294
(B OR C) AND A HP	14	6	5	6	0.7625	0.6586	0.8424	0.9084	0.8467	0.9468
(B OR C) AND A HP	14	6	6	1	0.7625	0.6586	0.8424	0.8015	0.7251	0.8608
(B OR C) AND A HP	14	6	6	2	0.7625	0.6586	0.8424	0.8473	0.7759	0.8989
(B OR C) AND A HP	14	6	6	3	0.7625	0.6586	0.8424	0.8779	0.8108	0.9234
(B OR C) AND A HP	14	6	6	4	0.7625	0.6586	0.8424	0.8779	0.8108	0.9234
(B OR C) AND A HP	14	6	6	5	0.7625	0.6586	0.8424	0.8855	0.8197	0.9294
(B OR C) AND A HP	14	6	6	6	0.7625	0.6586	0.8424	0.9084	0.8467	0.9468
(A OR C) AND B HP	15	1	1	1	0.9375	0.8619	0.9730	0.4580	0.3751	0.5433
(A OR C) AND B HP	15	1	1	2	0.9375	0.8619	0.9730	0.4580	0.3751	0.5433
(A OR C) AND B HP	15	1	1	3	0.9250	0.8459	0.9652	0.4885	0.4045	0.5732
(A OR C) AND B HP	15	1	1	4	0.9250	0.8459	0.9652	0.4885	0.4045	0.5732
(A OR C) AND B HP	15	1	1	5	0.9125	0.8302	0.9570	0.4962	0.4119	0.5807
(A OR C) AND B HP	15	1	1	6	0.9000	0.8149	0.9485	0.5115	0.4268	0.5955
(A OR C) AND B HP	15	1	2	1	0.8875	0.7998	0.9397	0.5649	0.4793	0.6467
(A OR C) AND B HP	15	1	2	2	0.8875	0.7998	0.9397	0.5649	0.4793	0.6467
(A OR C) AND B HP	15	1	2	3	0.8750	0.7850	0.9307	0.5878	0.5022	0.6684
(A OR C) AND B HP	15	1	2	4	0.8750	0.7850	0.9307	0.5878	0.5022	0.6684
(A OR C) AND B HP	15	1	2	5	0.8750	0.7850	0.9307	0.5954	0.5098	0.6756
(A OR C) AND B HP	15	1	2	6	0.8625	0.7703	0.9215	0.6107	0.5252	0.6899
(A OR C) AND B HP	15	1	3	1	0.8875	0.7998	0.9397	0.7023	0.6191	0.7739
(A OR C) AND B HP	15	1	3	2	0.8875	0.7998	0.9397	0.7023	0.6191	0.7739
(A OR C) AND B HP	15	1	3	3	0.8750	0.7850	0.9307	0.7176	0.6351	0.7876
(A OR C) AND B HP	15	1	3	4	0.8750	0.7850	0.9307	0.7176	0.6351	0.7876
(A OR C) AND B HP	15	1	3	5	0.8750	0.7850	0.9307	0.7252	0.6432	0.7944
(A OR C) AND B HP	15	1	3	6	0.8625	0.7703	0.9215	0.7328	0.6512	0.8012
(A OR C) AND B HP	15	1	4	1	0.8125	0.7134	0.8829	0.8168	0.7419	0.8737
(A OR C) AND B HP	15	1	4	2	0.8125	0.7134	0.8829	0.8168	0.7419	0.8737
(A OR C) AND B HP	15	1	4	3	0.8125	0.7134	0.8829	0.8168	0.7419	0.8737
(A OR C) AND B HP	15	1	4	4	0.8125	0.7134	0.8829	0.8168	0.7419	0.8737
(A OR C) AND B HP	15	1	4	5	0.8125	0.7134	0.8829	0.8244	0.7503	0.8801
(A OR C) AND B HP	15	1	4	6	0.8000	0.6995	0.8730	0.8321	0.7588	0.8864
(A OR C) AND B HP	15	1	5	1	0.8000	0.6995	0.8730	0.8473	0.7759	0.8989
(A OR C) AND B HP	15	1	5	2	0.8000	0.6995	0.8730	0.8473	0.7759	0.8989
(A OR C) AND B HP	15	1	5	3	0.8000	0.6995	0.8730	0.8473	0.7759	0.8989
(A OR C) AND B HP	15	1	5	4	0.8000	0.6995	0.8730	0.8473	0.7759	0.8989
(A OR C) AND B HP	15	1	5	5	0.8000	0.6995	0.8730	0.8550	0.7846	0.9051
(A OR C) AND B HP	15	1	5	6	0.8000	0.6995	0.8730	0.8626	0.7932	0.9113
(A OR C) AND B HP	15	1	6	1	0.7250	0.6186	0.8108	0.8779	0.8108	0.9234
(A OR C) AND B HP	15	1	6	2	0.7250	0.6186	0.8108	0.8779	0.8108	0.9234
(A OR C) AND B HP	15	1	6	3	0.7250	0.6186	0.8108	0.8779	0.8108	0.9234
(A OR C) AND B HP	15	1	6	4	0.7250	0.6186	0.8108	0.8779	0.8108	0.9234
(A OR C) AND B HP	15	1	6	5	0.7250	0.6186	0.8108	0.8779	0.8108	0.9234
(A OR C) AND B HP	15	1	6	6	0.7250	0.6186	0.8108	0.8855	0.8197	0.9294
(A OR C) AND B HP	15	2	1	1	0.9375	0.8619	0.9730	0.4733	0.3898	0.5583
(A OR C) AND B HP	15	2	1	2	0.9375	0.8619	0.9730	0.4885	0.4045	0.5732
(A OR C) AND B HP	15	2	1	3	0.9250	0.8459	0.9652	0.5191	0.4342	0.6029
(A OR C) AND B HP	15	2	1	4	0.9250	0.8459	0.9652	0.5191	0.4342	0.6029

		Threshold				95% CI			95% CI	
Combination	EK	A	B	C	Sensitivity	Lower	Upper	Specificity	Lower	Upper
(A OR C) AND B HP	15	2	1	5	0.9125	0.8302	0.9570	0.5344	0.4492	0.6176
(A OR C) AND B HP	15	2	1	6	0.8875	0.7998	0.9397	0.5573	0.4718	0.6395
(A OR C) AND B HP	15	2	2	1	0.8875	0.7998	0.9397	0.5802	0.4945	0.6612
(A OR C) AND B HP	15	2	2	2	0.8875	0.7998	0.9397	0.5954	0.5098	0.6756
(A OR C) AND B HP	15	2	2	3	0.8750	0.7850	0.9307	0.6183	0.5329	0.6970
(A OR C) AND B HP	15	2	2	4	0.8750	0.7850	0.9307	0.6183	0.5329	0.6970
(A OR C) AND B HP	15	2	2	5	0.8750	0.7850	0.9307	0.6260	0.5406	0.7041
(A OR C) AND B HP	15	2	2	6	0.8500	0.7559	0.9121	0.6412	0.5561	0.7183
(A OR C) AND B HP	15	2	3	1	0.8875	0.7998	0.9397	0.7099	0.6271	0.7808
(A OR C) AND B HP	15	2	3	2	0.8875	0.7998	0.9397	0.7252	0.6432	0.7944
(A OR C) AND B HP	15	2	3	3	0.8750	0.7850	0.9307	0.7405	0.6593	0.8079
(A OR C) AND B HP	15	2	3	4	0.8750	0.7850	0.9307	0.7405	0.6593	0.8079
(A OR C) AND B HP	15	2	3	5	0.8750	0.7850	0.9307	0.7481	0.6674	0.8146
(A OR C) AND B HP	15	2	3	6	0.8500	0.7559	0.9121	0.7557	0.6756	0.8213
(A OR C) AND B HP	15	2	4	1	0.8125	0.7134	0.8829	0.8244	0.7503	0.8801
(A OR C) AND B HP	15	2	4	2	0.8125	0.7134	0.8829	0.8321	0.7588	0.8864
(A OR C) AND B HP	15	2	4	3	0.8125	0.7134	0.8829	0.8321	0.7588	0.8864
(A OR C) AND B HP	15	2	4	4	0.8125	0.7134	0.8829	0.8321	0.7588	0.8864
(A OR C) AND B HP	15	2	4	5	0.8125	0.7134	0.8829	0.8397	0.7673	0.8927
(A OR C) AND B HP	15	2	4	6	0.7875	0.6858	0.8629	0.8473	0.7759	0.8989
(A OR C) AND B HP	15	2	5	1	0.8000	0.6995	0.8730	0.8473	0.7759	0.8989
(A OR C) AND B HP	15	2	5	2	0.8000	0.6995	0.8730	0.8473	0.7759	0.8989
(A OR C) AND B HP	15	2	5	3	0.8000	0.6995	0.8730	0.8473	0.7759	0.8989
(A OR C) AND B HP	15	2	5	4	0.8000	0.6995	0.8730	0.8473	0.7759	0.8989
(A OR C) AND B HP	15	2	5	5	0.8000	0.6995	0.8730	0.8550	0.7846	0.9051
(A OR C) AND B HP	15	2	5	6	0.7875	0.6858	0.8629	0.8626	0.7932	0.9113
(A OR C) AND B HP	15	2	6	1	0.7250	0.6186	0.8108	0.8779	0.8108	0.9234
(A OR C) AND B HP	15	2	6	2	0.7250	0.6186	0.8108	0.8779	0.8108	0.9234
(A OR C) AND B HP	15	2	6	3	0.7250	0.6186	0.8108	0.8779	0.8108	0.9234
(A OR C) AND B HP	15	2	6	4	0.7250	0.6186	0.8108	0.8779	0.8108	0.9234
(A OR C) AND B HP	15	2	6	5	0.7250	0.6186	0.8108	0.8779	0.8108	0.9234
(A OR C) AND B HP	15	2	6	6	0.7125	0.6054	0.8001	0.8855	0.8197	0.9294
(A OR C) AND B HP	15	3	1	1	0.9375	0.8619	0.9730	0.4885	0.4045	0.5732
(A OR C) AND B HP	15	3	1	2	0.9375	0.8619	0.9730	0.5115	0.4268	0.5955
(A OR C) AND B HP	15	3	1	3	0.9250	0.8459	0.9652	0.5420	0.4567	0.6249
(A OR C) AND B HP	15	3	1	4	0.9250	0.8459	0.9652	0.5496	0.4642	0.6322
(A OR C) AND B HP	15	3	1	5	0.9125	0.8302	0.9570	0.5802	0.4945	0.6612
(A OR C) AND B HP	15	3	1	6	0.8875	0.7998	0.9397	0.6183	0.5329	0.6970
(A OR C) AND B HP	15	3	2	1	0.8875	0.7998	0.9397	0.5954	0.5098	0.6756
(A OR C) AND B HP	15	3	2	2	0.8875	0.7998	0.9397	0.6183	0.5329	0.6970
(A OR C) AND B HP	15	3	2	3	0.8750	0.7850	0.9307	0.6412	0.5561	0.7183
(A OR C) AND B HP	15	3	2	4	0.8750	0.7850	0.9307	0.6489	0.5639	0.7253
(A OR C) AND B HP	15	3	2	5	0.8750	0.7850	0.9307	0.6718	0.5875	0.7463
(A OR C) AND B HP	15	3	2	6	0.8500	0.7559	0.9121	0.6947	0.6112	0.7670
(A OR C) AND B HP	15	3	3	1	0.8875	0.7998	0.9397	0.7252	0.6432	0.7944
(A OR C) AND B HP	15	3	3	2	0.8875	0.7998	0.9397	0.7481	0.6674	0.8146
(A OR C) AND B HP	15	3	3	3	0.8750	0.7850	0.9307	0.7634	0.6837	0.8280
(A OR C) AND B HP	15	3	3	4	0.8750	0.7850	0.9307	0.7634	0.6837	0.8280
(A OR C) AND B HP	15	3	3	5	0.8750	0.7850	0.9307	0.7863	0.7084	0.8478
(A OR C) AND B HP	15	3	3	6	0.8500	0.7559	0.9121	0.8015	0.7251	0.8608
(A OR C) AND B HP	15	3	4	1	0.8125	0.7134	0.8829	0.8321	0.7588	0.8864
(A OR C) AND B HP	15	3	4	2	0.8125	0.7134	0.8829	0.8397	0.7673	0.8927
(A OR C) AND B HP	15	3	4	3	0.8125	0.7134	0.8829	0.8397	0.7673	0.8927

		Threshold				95% CI			95% CI	
Combination	EK	A	B	C	Sensitivity	Lower	Upper	Specificity	Lower	Upper
(A OR C) AND B HP	15	3	4	4	0.8125	0.7134	0.8829	0.8397	0.7673	0.8927
(A OR C) AND B HP	15	3	4	5	0.8125	0.7134	0.8829	0.8473	0.7759	0.8989
(A OR C) AND B HP	15	3	4	6	0.7875	0.6858	0.8629	0.8626	0.7932	0.9113
(A OR C) AND B HP	15	3	5	1	0.8000	0.6995	0.8730	0.8550	0.7846	0.9051
(A OR C) AND B HP	15	3	5	2	0.8000	0.6995	0.8730	0.8550	0.7846	0.9051
(A OR C) AND B HP	15	3	5	3	0.8000	0.6995	0.8730	0.8550	0.7846	0.9051
(A OR C) AND B HP	15	3	5	4	0.8000	0.6995	0.8730	0.8550	0.7846	0.9051
(A OR C) AND B HP	15	3	5	5	0.8000	0.6995	0.8730	0.8626	0.7932	0.9113
(A OR C) AND B HP	15	3	5	6	0.7875	0.6858	0.8629	0.8702	0.8020	0.9174
(A OR C) AND B HP	15	3	6	1	0.7250	0.6186	0.8108	0.8779	0.8108	0.9234
(A OR C) AND B HP	15	3	6	2	0.7250	0.6186	0.8108	0.8779	0.8108	0.9234
(A OR C) AND B HP	15	3	6	3	0.7250	0.6186	0.8108	0.8779	0.8108	0.9234
(A OR C) AND B HP	15	3	6	4	0.7250	0.6186	0.8108	0.8779	0.8108	0.9234
(A OR C) AND B HP	15	3	6	5	0.7250	0.6186	0.8108	0.8779	0.8108	0.9234
(A OR C) AND B HP	15	3	6	6	0.7125	0.6054	0.8001	0.8855	0.8197	0.9294
(A OR C) AND B HP	15	4	1	1	0.9375	0.8619	0.9730	0.5038	0.4193	0.5881
(A OR C) AND B HP	15	4	1	2	0.9375	0.8619	0.9730	0.5267	0.4417	0.6102
(A OR C) AND B HP	15	4	1	3	0.9250	0.8459	0.9652	0.5573	0.4718	0.6395
(A OR C) AND B HP	15	4	1	4	0.9250	0.8459	0.9652	0.5802	0.4945	0.6612
(A OR C) AND B HP	15	4	1	5	0.9000	0.8149	0.9485	0.6107	0.5252	0.6899
(A OR C) AND B HP	15	4	1	6	0.8625	0.7703	0.9215	0.6641	0.5796	0.7393
(A OR C) AND B HP	15	4	2	1	0.8875	0.7998	0.9397	0.6107	0.5252	0.6899
(A OR C) AND B HP	15	4	2	2	0.8875	0.7998	0.9397	0.6336	0.5484	0.7112
(A OR C) AND B HP	15	4	2	3	0.8750	0.7850	0.9307	0.6565	0.5718	0.7323
(A OR C) AND B HP	15	4	2	4	0.8750	0.7850	0.9307	0.6718	0.5875	0.7463
(A OR C) AND B HP	15	4	2	5	0.8625	0.7703	0.9215	0.6947	0.6112	0.7670
(A OR C) AND B HP	15	4	2	6	0.8250	0.7274	0.8928	0.7328	0.6512	0.8012
(A OR C) AND B HP	15	4	3	1	0.8875	0.7998	0.9397	0.7405	0.6593	0.8079
(A OR C) AND B HP	15	4	3	2	0.8875	0.7998	0.9397	0.7634	0.6837	0.8280
(A OR C) AND B HP	15	4	3	3	0.8750	0.7850	0.9307	0.7786	0.7002	0.8412
(A OR C) AND B HP	15	4	3	4	0.8750	0.7850	0.9307	0.7863	0.7084	0.8478
(A OR C) AND B HP	15	4	3	5	0.8625	0.7703	0.9215	0.8092	0.7334	0.8673
(A OR C) AND B HP	15	4	3	6	0.8250	0.7274	0.8928	0.8397	0.7673	0.8927
(A OR C) AND B HP	15	4	4	1	0.8125	0.7134	0.8829	0.8321	0.7588	0.8864
(A OR C) AND B HP	15	4	4	2	0.8125	0.7134	0.8829	0.8397	0.7673	0.8927
(A OR C) AND B HP	15	4	4	3	0.8125	0.7134	0.8829	0.8397	0.7673	0.8927
(A OR C) AND B HP	15	4	4	4	0.8125	0.7134	0.8829	0.8397	0.7673	0.8927
(A OR C) AND B HP	15	4	4	5	0.8125	0.7134	0.8829	0.8473	0.7759	0.8989
(A OR C) AND B HP	15	4	4	6	0.7875	0.6858	0.8629	0.8702	0.8020	0.9174
(A OR C) AND B HP	15	4	5	1	0.8000	0.6995	0.8730	0.8550	0.7846	0.9051
(A OR C) AND B HP	15	4	5	2	0.8000	0.6995	0.8730	0.8550	0.7846	0.9051
(A OR C) AND B HP	15	4	5	3	0.8000	0.6995	0.8730	0.8550	0.7846	0.9051
(A OR C) AND B HP	15	4	5	4	0.8000	0.6995	0.8730	0.8550	0.7846	0.9051
(A OR C) AND B HP	15	4	5	5	0.8000	0.6995	0.8730	0.8626	0.7932	0.9113
(A OR C) AND B HP	15	4	5	6	0.7875	0.6858	0.8629	0.8779	0.8108	0.9234
(A OR C) AND B HP	15	4	6	1	0.7250	0.6186	0.8108	0.8779	0.8108	0.9234
(A OR C) AND B HP	15	4	6	2	0.7250	0.6186	0.8108	0.8779	0.8108	0.9234
(A OR C) AND B HP	15	4	6	3	0.7250	0.6186	0.8108	0.8779	0.8108	0.9234
(A OR C) AND B HP	15	4	6	4	0.7250	0.6186	0.8108	0.8779	0.8108	0.9234
(A OR C) AND B HP	15	4	6	5	0.7250	0.6186	0.8108	0.8779	0.8108	0.9234
(A OR C) AND B HP	15	4	6	6	0.7125	0.6054	0.8001	0.8931	0.8286	0.9353
(A OR C) AND B HP	15	5	1	1	0.9375	0.8619	0.9730	0.5115	0.4268	0.5955
(A OR C) AND B HP	15	5	1	2	0.9375	0.8619	0.9730	0.5344	0.4492	0.6176

		Threshold				95% CI			95% CI	
Combination	EK	A	B	C	Sensitivity	Lower	Upper	Specificity	Lower	Upper
(A OR C) AND B HP	15	5	1	3	0.9250	0.8459	0.9652	0.5649	0.4793	0.6467
(A OR C) AND B HP	15	5	1	4	0.9250	0.8459	0.9652	0.5878	0.5022	0.6684
(A OR C) AND B HP	15	5	1	5	0.9000	0.8149	0.9485	0.6183	0.5329	0.6970
(A OR C) AND B HP	15	5	1	6	0.8500	0.7559	0.9121	0.6947	0.6112	0.7670
(A OR C) AND B HP	15	5	2	1	0.8875	0.7998	0.9397	0.6183	0.5329	0.6970
(A OR C) AND B HP	15	5	2	2	0.8875	0.7998	0.9397	0.6412	0.5561	0.7183
(A OR C) AND B HP	15	5	2	3	0.8750	0.7850	0.9307	0.6641	0.5796	0.7393
(A OR C) AND B HP	15	5	2	4	0.8750	0.7850	0.9307	0.6794	0.5953	0.7532
(A OR C) AND B HP	15	5	2	5	0.8625	0.7703	0.9215	0.7023	0.6191	0.7739
(A OR C) AND B HP	15	5	2	6	0.8125	0.7134	0.8829	0.7557	0.6756	0.8213
(A OR C) AND B HP	15	5	3	1	0.8875	0.7998	0.9397	0.7405	0.6593	0.8079
(A OR C) AND B HP	15	5	3	2	0.8875	0.7998	0.9397	0.7634	0.6837	0.8280
(A OR C) AND B HP	15	5	3	3	0.8750	0.7850	0.9307	0.7786	0.7002	0.8412
(A OR C) AND B HP	15	5	3	4	0.8750	0.7850	0.9307	0.7863	0.7084	0.8478
(A OR C) AND B HP	15	5	3	5	0.8625	0.7703	0.9215	0.8092	0.7334	0.8673
(A OR C) AND B HP	15	5	3	6	0.8125	0.7134	0.8829	0.8473	0.7759	0.8989
(A OR C) AND B HP	15	5	4	1	0.8125	0.7134	0.8829	0.8321	0.7588	0.8864
(A OR C) AND B HP	15	5	4	2	0.8125	0.7134	0.8829	0.8397	0.7673	0.8927
(A OR C) AND B HP	15	5	4	3	0.8125	0.7134	0.8829	0.8397	0.7673	0.8927
(A OR C) AND B HP	15	5	4	4	0.8125	0.7134	0.8829	0.8397	0.7673	0.8927
(A OR C) AND B HP	15	5	4	5	0.8125	0.7134	0.8829	0.8473	0.7759	0.8989
(A OR C) AND B HP	15	5	4	6	0.7750	0.6721	0.8527	0.8702	0.8020	0.9174
(A OR C) AND B HP	15	5	5	1	0.8000	0.6995	0.8730	0.8550	0.7846	0.9051
(A OR C) AND B HP	15	5	5	2	0.8000	0.6995	0.8730	0.8550	0.7846	0.9051
(A OR C) AND B HP	15	5	5	3	0.8000	0.6995	0.8730	0.8550	0.7846	0.9051
(A OR C) AND B HP	15	5	5	4	0.8000	0.6995	0.8730	0.8550	0.7846	0.9051
(A OR C) AND B HP	15	5	5	5	0.8000	0.6995	0.8730	0.8626	0.7932	0.9113
(A OR C) AND B HP	15	5	5	6	0.7750	0.6721	0.8527	0.8779	0.8108	0.9234
(A OR C) AND B HP	15	5	6	1	0.7250	0.6186	0.8108	0.8779	0.8108	0.9234
(A OR C) AND B HP	15	5	6	2	0.7250	0.6186	0.8108	0.8779	0.8108	0.9234
(A OR C) AND B HP	15	5	6	3	0.7250	0.6186	0.8108	0.8779	0.8108	0.9234
(A OR C) AND B HP	15	5	6	4	0.7250	0.6186	0.8108	0.8779	0.8108	0.9234
(A OR C) AND B HP	15	5	6	5	0.7250	0.6186	0.8108	0.8779	0.8108	0.9234
(A OR C) AND B HP	15	5	6	6	0.7125	0.6054	0.8001	0.8931	0.8286	0.9353
(A OR C) AND B HP	15	6	1	1	0.9375	0.8619	0.9730	0.5115	0.4268	0.5955
(A OR C) AND B HP	15	6	1	2	0.9375	0.8619	0.9730	0.5420	0.4567	0.6249
(A OR C) AND B HP	15	6	1	3	0.9250	0.8459	0.9652	0.5802	0.4945	0.6612
(A OR C) AND B HP	15	6	1	4	0.9250	0.8459	0.9652	0.6031	0.5175	0.6828
(A OR C) AND B HP	15	6	1	5	0.9000	0.8149	0.9485	0.6489	0.5639	0.7253
(A OR C) AND B HP	15	6	1	6	0.8375	0.7416	0.9025	0.7252	0.6432	0.7944
(A OR C) AND B HP	15	6	2	1	0.8875	0.7998	0.9397	0.6183	0.5329	0.6970
(A OR C) AND B HP	15	6	2	2	0.8875	0.7998	0.9397	0.6489	0.5639	0.7253
(A OR C) AND B HP	15	6	2	3	0.8750	0.7850	0.9307	0.6794	0.5953	0.7532
(A OR C) AND B HP	15	6	2	4	0.8750	0.7850	0.9307	0.6947	0.6112	0.7670
(A OR C) AND B HP	15	6	2	5	0.8625	0.7703	0.9215	0.7328	0.6512	0.8012
(A OR C) AND B HP	15	6	2	6	0.8000	0.6995	0.8730	0.7863	0.7084	0.8478
(A OR C) AND B HP	15	6	3	1	0.8875	0.7998	0.9397	0.7405	0.6593	0.8079
(A OR C) AND B HP	15	6	3	2	0.8875	0.7998	0.9397	0.7710	0.6919	0.8346
(A OR C) AND B HP	15	6	3	3	0.8750	0.7850	0.9307	0.7939	0.7167	0.8543
(A OR C) AND B HP	15	6	3	4	0.8750	0.7850	0.9307	0.8015	0.7251	0.8608
(A OR C) AND B HP	15	6	3	5	0.8625	0.7703	0.9215	0.8321	0.7588	0.8864
(A OR C) AND B HP	15	6	3	6	0.8000	0.6995	0.8730	0.8702	0.8020	0.9174
(A OR C) AND B HP	15	6	4	1	0.8125	0.7134	0.8829	0.8321	0.7588	0.8864

		Threshold				95% CI			95% CI	
Combination	EK	A	B	C	Sensitivity	Lower	Upper	Specificity	Lower	Upper
(A OR C) AND B HP	15	6	4	2	0.8125	0.7134	0.8829	0.8473	0.7759	0.8989
(A OR C) AND B HP	15	6	4	3	0.8125	0.7134	0.8829	0.8550	0.7846	0.9051
(A OR C) AND B HP	15	6	4	4	0.8125	0.7134	0.8829	0.8550	0.7846	0.9051
(A OR C) AND B HP	15	6	4	5	0.8125	0.7134	0.8829	0.8702	0.8020	0.9174
(A OR C) AND B HP	15	6	4	6	0.7625	0.6586	0.8424	0.8931	0.8286	0.9353
(A OR C) AND B HP	15	6	5	1	0.8000	0.6995	0.8730	0.8550	0.7846	0.9051
(A OR C) AND B HP	15	6	5	2	0.8000	0.6995	0.8730	0.8626	0.7932	0.9113
(A OR C) AND B HP	15	6	5	3	0.8000	0.6995	0.8730	0.8702	0.8020	0.9174
(A OR C) AND B HP	15	6	5	4	0.8000	0.6995	0.8730	0.8702	0.8020	0.9174
(A OR C) AND B HP	15	6	5	5	0.8000	0.6995	0.8730	0.8855	0.8197	0.9294
(A OR C) AND B HP	15	6	5	6	0.7625	0.6586	0.8424	0.9008	0.8376	0.9411
(A OR C) AND B HP	15	6	6	1	0.7250	0.6186	0.8108	0.8779	0.8108	0.9234
(A OR C) AND B HP	15	6	6	2	0.7250	0.6186	0.8108	0.8855	0.8197	0.9294
(A OR C) AND B HP	15	6	6	3	0.7250	0.6186	0.8108	0.8931	0.8286	0.9353
(A OR C) AND B HP	15	6	6	4	0.7250	0.6186	0.8108	0.8931	0.8286	0.9353
(A OR C) AND B HP	15	6	6	5	0.7250	0.6186	0.8108	0.8931	0.8286	0.9353
(A OR C) AND B HP	15	6	6	6	0.7000	0.5923	0.7894	0.9084	0.8467	0.9468
(A AND B) OR C HP	16	1	1	1	0.9875	0.9325	0.9978	0.1832	0.1263	0.2581
(A AND B) OR C HP	16	1	1	2	0.9875	0.9325	0.9978	0.2366	0.1720	0.3163
(A AND B) OR C HP	16	1	1	3	0.9500	0.8784	0.9804	0.3282	0.2537	0.4125
(A AND B) OR C HP	16	1	1	4	0.9500	0.8784	0.9804	0.3511	0.2747	0.4361
(A AND B) OR C HP	16	1	1	5	0.9375	0.8619	0.9730	0.3740	0.2959	0.4594
(A AND B) OR C HP	16	1	1	6	0.9250	0.8459	0.9652	0.4351	0.3533	0.5207
(A AND B) OR C HP	16	1	2	1	0.9875	0.9325	0.9978	0.1908	0.1327	0.2666
(A AND B) OR C HP	16	1	2	2	0.9875	0.9325	0.9978	0.2595	0.1921	0.3407
(A AND B) OR C HP	16	1	2	3	0.9500	0.8784	0.9804	0.3511	0.2747	0.4361
(A AND B) OR C HP	16	1	2	4	0.9500	0.8784	0.9804	0.3817	0.3030	0.4671
(A AND B) OR C HP	16	1	2	5	0.9375	0.8619	0.9730	0.4122	0.3316	0.4978
(A AND B) OR C HP	16	1	2	6	0.9250	0.8459	0.9652	0.4962	0.4119	0.5807
(A AND B) OR C HP	16	1	3	1	0.9875	0.9325	0.9978	0.2061	0.1457	0.2833
(A AND B) OR C HP	16	1	3	2	0.9875	0.9325	0.9978	0.2824	0.2124	0.3649
(A AND B) OR C HP	16	1	3	3	0.9500	0.8784	0.9804	0.3817	0.3030	0.4671
(A AND B) OR C HP	16	1	3	4	0.9500	0.8784	0.9804	0.4198	0.3388	0.5055
(A AND B) OR C HP	16	1	3	5	0.9375	0.8619	0.9730	0.4580	0.3751	0.5433
(A AND B) OR C HP	16	1	3	6	0.9250	0.8459	0.9652	0.5573	0.4718	0.6395
(A AND B) OR C HP	16	1	4	1	0.9875	0.9325	0.9978	0.2366	0.1720	0.3163
(A AND B) OR C HP	16	1	4	2	0.9875	0.9325	0.9978	0.3282	0.2537	0.4125
(A AND B) OR C HP	16	1	4	3	0.9500	0.8784	0.9804	0.4275	0.3460	0.5131
(A AND B) OR C HP	16	1	4	4	0.9500	0.8784	0.9804	0.4733	0.3898	0.5583
(A AND B) OR C HP	16	1	4	5	0.9250	0.8459	0.9652	0.5267	0.4417	0.6102
(A AND B) OR C HP	16	1	4	6	0.9000	0.8149	0.9485	0.6412	0.5561	0.7183
(A AND B) OR C HP	16	1	5	1	0.9875	0.9325	0.9978	0.2443	0.1787	0.3244
(A AND B) OR C HP	16	1	5	2	0.9875	0.9325	0.9978	0.3435	0.2677	0.4282
(A AND B) OR C HP	16	1	5	3	0.9500	0.8784	0.9804	0.4427	0.3605	0.5282
(A AND B) OR C HP	16	1	5	4	0.9500	0.8784	0.9804	0.4885	0.4045	0.5732
(A AND B) OR C HP	16	1	5	5	0.9250	0.8459	0.9652	0.5420	0.4567	0.6249
(A AND B) OR C HP	16	1	5	6	0.9000	0.8149	0.9485	0.6641	0.5796	0.7393
(A AND B) OR C HP	16	1	6	1	0.9875	0.9325	0.9978	0.2519	0.1854	0.3326
(A AND B) OR C HP	16	1	6	2	0.9875	0.9325	0.9978	0.3511	0.2747	0.4361
(A AND B) OR C HP	16	1	6	3	0.9500	0.8784	0.9804	0.4504	0.3678	0.5358
(A AND B) OR C HP	16	1	6	4	0.9500	0.8784	0.9804	0.4962	0.4119	0.5807
(A AND B) OR C HP	16	1	6	5	0.9250	0.8459	0.9652	0.5573	0.4718	0.6395
(A AND B) OR C HP	16	1	6	6	0.8875	0.7998	0.9397	0.6794	0.5953	0.7532

		Threshold				95% CI			95% CI	
Combination	EK	A	B	C	Sensitivity	Lower	Upper	Specificity	Lower	Upper
(A AND B) OR C HP	16	2	1	1	0.9875	0.9325	0.9978	0.1985	0.1392	0.2749
(A AND B) OR C HP	16	2	1	2	0.9875	0.9325	0.9978	0.2672	0.1988	0.3488
(A AND B) OR C HP	16	2	1	3	0.9500	0.8784	0.9804	0.3588	0.2817	0.4439
(A AND B) OR C HP	16	2	1	4	0.9500	0.8784	0.9804	0.3817	0.3030	0.4671
(A AND B) OR C HP	16	2	1	5	0.9375	0.8619	0.9730	0.4122	0.3316	0.4978
(A AND B) OR C HP	16	2	1	6	0.9125	0.8302	0.9570	0.4809	0.3971	0.5658
(A AND B) OR C HP	16	2	2	1	0.9875	0.9325	0.9978	0.2061	0.1457	0.2833
(A AND B) OR C HP	16	2	2	2	0.9875	0.9325	0.9978	0.2901	0.2192	0.3729
(A AND B) OR C HP	16	2	2	3	0.9500	0.8784	0.9804	0.3817	0.3030	0.4671
(A AND B) OR C HP	16	2	2	4	0.9500	0.8784	0.9804	0.4122	0.3316	0.4978
(A AND B) OR C HP	16	2	2	5	0.9375	0.8619	0.9730	0.4427	0.3605	0.5282
(A AND B) OR C HP	16	2	2	6	0.9125	0.8302	0.9570	0.5267	0.4417	0.6102
(A AND B) OR C HP	16	2	3	1	0.9875	0.9325	0.9978	0.2137	0.1522	0.2916
(A AND B) OR C HP	16	2	3	2	0.9875	0.9325	0.9978	0.3053	0.2330	0.3888
(A AND B) OR C HP	16	2	3	3	0.9500	0.8784	0.9804	0.4046	0.3244	0.4902
(A AND B) OR C HP	16	2	3	4	0.9500	0.8784	0.9804	0.4427	0.3605	0.5282
(A AND B) OR C HP	16	2	3	5	0.9375	0.8619	0.9730	0.4809	0.3971	0.5658
(A AND B) OR C HP	16	2	3	6	0.9125	0.8302	0.9570	0.5802	0.4945	0.6612
(A AND B) OR C HP	16	2	4	1	0.9875	0.9325	0.9978	0.2443	0.1787	0.3244
(A AND B) OR C HP	16	2	4	2	0.9875	0.9325	0.9978	0.3435	0.2677	0.4282
(A AND B) OR C HP	16	2	4	3	0.9500	0.8784	0.9804	0.4427	0.3605	0.5282
(A AND B) OR C HP	16	2	4	4	0.9500	0.8784	0.9804	0.4885	0.4045	0.5732
(A AND B) OR C HP	16	2	4	5	0.9250	0.8459	0.9652	0.5420	0.4567	0.6249
(A AND B) OR C HP	16	2	4	6	0.8875	0.7998	0.9397	0.6565	0.5718	0.7323
(A AND B) OR C HP	16	2	5	1	0.9875	0.9325	0.9978	0.2443	0.1787	0.3244
(A AND B) OR C HP	16	2	5	2	0.9875	0.9325	0.9978	0.3435	0.2677	0.4282
(A AND B) OR C HP	16	2	5	3	0.9500	0.8784	0.9804	0.4427	0.3605	0.5282
(A AND B) OR C HP	16	2	5	4	0.9500	0.8784	0.9804	0.4885	0.4045	0.5732
(A AND B) OR C HP	16	2	5	5	0.9250	0.8459	0.9652	0.5420	0.4567	0.6249
(A AND B) OR C HP	16	2	5	6	0.8875	0.7998	0.9397	0.6641	0.5796	0.7393
(A AND B) OR C HP	16	2	6	1	0.9875	0.9325	0.9978	0.2519	0.1854	0.3326
(A AND B) OR C HP	16	2	6	2	0.9875	0.9325	0.9978	0.3511	0.2747	0.4361
(A AND B) OR C HP	16	2	6	3	0.9500	0.8784	0.9804	0.4504	0.3678	0.5358
(A AND B) OR C HP	16	2	6	4	0.9500	0.8784	0.9804	0.4962	0.4119	0.5807
(A AND B) OR C HP	16	2	6	5	0.9250	0.8459	0.9652	0.5573	0.4718	0.6395
(A AND B) OR C HP	16	2	6	6	0.8750	0.7850	0.9307	0.6794	0.5953	0.7532
(A AND B) OR C HP	16	3	1	1	0.9875	0.9325	0.9978	0.2137	0.1522	0.2916
(A AND B) OR C HP	16	3	1	2	0.9875	0.9325	0.9978	0.2901	0.2192	0.3729
(A AND B) OR C HP	16	3	1	3	0.9500	0.8784	0.9804	0.3817	0.3030	0.4671
(A AND B) OR C HP	16	3	1	4	0.9500	0.8784	0.9804	0.4122	0.3316	0.4978
(A AND B) OR C HP	16	3	1	5	0.9375	0.8619	0.9730	0.4580	0.3751	0.5433
(A AND B) OR C HP	16	3	1	6	0.9125	0.8302	0.9570	0.5420	0.4567	0.6249
(A AND B) OR C HP	16	3	2	1	0.9875	0.9325	0.9978	0.2214	0.1588	0.2998
(A AND B) OR C HP	16	3	2	2	0.9875	0.9325	0.9978	0.3130	0.2399	0.3968
(A AND B) OR C HP	16	3	2	3	0.9500	0.8784	0.9804	0.4046	0.3244	0.4902
(A AND B) OR C HP	16	3	2	4	0.9500	0.8784	0.9804	0.4427	0.3605	0.5282
(A AND B) OR C HP	16	3	2	5	0.9375	0.8619	0.9730	0.4885	0.4045	0.5732
(A AND B) OR C HP	16	3	2	6	0.9125	0.8302	0.9570	0.5802	0.4945	0.6612
(A AND B) OR C HP	16	3	3	1	0.9875	0.9325	0.9978	0.2290	0.1654	0.3081
(A AND B) OR C HP	16	3	3	2	0.9875	0.9325	0.9978	0.3282	0.2537	0.4125
(A AND B) OR C HP	16	3	3	3	0.9500	0.8784	0.9804	0.4275	0.3460	0.5131
(A AND B) OR C HP	16	3	3	4	0.9500	0.8784	0.9804	0.4656	0.3824	0.5508
(A AND B) OR C HP	16	3	3	5	0.9375	0.8619	0.9730	0.5191	0.4342	0.6029

		Threshold				95% CI			95% CI	
Combination	EK	A	B	C	Sensitivity	Lower	Upper	Specificity	Lower	Upper
(A AND B) OR C HP	16	3	3	6	0.9125	0.8302	0.9570	0.6260	0.5406	0.7041
(A AND B) OR C HP	16	3	4	1	0.9875	0.9325	0.9978	0.2519	0.1854	0.3326
(A AND B) OR C HP	16	3	4	2	0.9875	0.9325	0.9978	0.3511	0.2747	0.4361
(A AND B) OR C HP	16	3	4	3	0.9500	0.8784	0.9804	0.4504	0.3678	0.5358
(A AND B) OR C HP	16	3	4	4	0.9500	0.8784	0.9804	0.4962	0.4119	0.5807
(A AND B) OR C HP	16	3	4	5	0.9250	0.8459	0.9652	0.5496	0.4642	0.6322
(A AND B) OR C HP	16	3	4	6	0.8875	0.7998	0.9397	0.6718	0.5875	0.7463
(A AND B) OR C HP	16	3	5	1	0.9875	0.9325	0.9978	0.2519	0.1854	0.3326
(A AND B) OR C HP	16	3	5	2	0.9875	0.9325	0.9978	0.3511	0.2747	0.4361
(A AND B) OR C HP	16	3	5	3	0.9500	0.8784	0.9804	0.4504	0.3678	0.5358
(A AND B) OR C HP	16	3	5	4	0.9500	0.8784	0.9804	0.4962	0.4119	0.5807
(A AND B) OR C HP	16	3	5	5	0.9250	0.8459	0.9652	0.5496	0.4642	0.6322
(A AND B) OR C HP	16	3	5	6	0.8875	0.7998	0.9397	0.6718	0.5875	0.7463
(A AND B) OR C HP	16	3	6	1	0.9875	0.9325	0.9978	0.2519	0.1854	0.3326
(A AND B) OR C HP	16	3	6	2	0.9875	0.9325	0.9978	0.3511	0.2747	0.4361
(A AND B) OR C HP	16	3	6	3	0.9500	0.8784	0.9804	0.4504	0.3678	0.5358
(A AND B) OR C HP	16	3	6	4	0.9500	0.8784	0.9804	0.4962	0.4119	0.5807
(A AND B) OR C HP	16	3	6	5	0.9250	0.8459	0.9652	0.5573	0.4718	0.6395
(A AND B) OR C HP	16	3	6	6	0.8750	0.7850	0.9307	0.6794	0.5953	0.7532
(A AND B) OR C HP	16	4	1	1	0.9875	0.9325	0.9978	0.2290	0.1654	0.3081
(A AND B) OR C HP	16	4	1	2	0.9875	0.9325	0.9978	0.3053	0.2330	0.3888
(A AND B) OR C HP	16	4	1	3	0.9500	0.8784	0.9804	0.3969	0.3172	0.4825
(A AND B) OR C HP	16	4	1	4	0.9500	0.8784	0.9804	0.4427	0.3605	0.5282
(A AND B) OR C HP	16	4	1	5	0.9250	0.8459	0.9652	0.4885	0.4045	0.5732
(A AND B) OR C HP	16	4	1	6	0.8875	0.7998	0.9397	0.5878	0.5022	0.6684
(A AND B) OR C HP	16	4	2	1	0.9875	0.9325	0.9978	0.2366	0.1720	0.3163
(A AND B) OR C HP	16	4	2	2	0.9875	0.9325	0.9978	0.3282	0.2537	0.4125
(A AND B) OR C HP	16	4	2	3	0.9500	0.8784	0.9804	0.4198	0.3388	0.5055
(A AND B) OR C HP	16	4	2	4	0.9500	0.8784	0.9804	0.4656	0.3824	0.5508
(A AND B) OR C HP	16	4	2	5	0.9250	0.8459	0.9652	0.5115	0.4268	0.5955
(A AND B) OR C HP	16	4	2	6	0.8875	0.7998	0.9397	0.6183	0.5329	0.6970
(A AND B) OR C HP	16	4	3	1	0.9875	0.9325	0.9978	0.2443	0.1787	0.3244
(A AND B) OR C HP	16	4	3	2	0.9875	0.9325	0.9978	0.3435	0.2677	0.4282
(A AND B) OR C HP	16	4	3	3	0.9500	0.8784	0.9804	0.4427	0.3605	0.5282
(A AND B) OR C HP	16	4	3	4	0.9500	0.8784	0.9804	0.4885	0.4045	0.5732
(A AND B) OR C HP	16	4	3	5	0.9250	0.8459	0.9652	0.5420	0.4567	0.6249
(A AND B) OR C HP	16	4	3	6	0.8875	0.7998	0.9397	0.6641	0.5796	0.7393
(A AND B) OR C HP	16	4	4	1	0.9875	0.9325	0.9978	0.2519	0.1854	0.3326
(A AND B) OR C HP	16	4	4	2	0.9875	0.9325	0.9978	0.3511	0.2747	0.4361
(A AND B) OR C HP	16	4	4	3	0.9500	0.8784	0.9804	0.4504	0.3678	0.5358
(A AND B) OR C HP	16	4	4	4	0.9500	0.8784	0.9804	0.4962	0.4119	0.5807
(A AND B) OR C HP	16	4	4	5	0.9250	0.8459	0.9652	0.5496	0.4642	0.6322
(A AND B) OR C HP	16	4	4	6	0.8875	0.7998	0.9397	0.6794	0.5953	0.7532
(A AND B) OR C HP	16	4	5	1	0.9875	0.9325	0.9978	0.2519	0.1854	0.3326
(A AND B) OR C HP	16	4	5	2	0.9875	0.9325	0.9978	0.3511	0.2747	0.4361
(A AND B) OR C HP	16	4	5	3	0.9500	0.8784	0.9804	0.4504	0.3678	0.5358
(A AND B) OR C HP	16	4	5	4	0.9500	0.8784	0.9804	0.4962	0.4119	0.5807
(A AND B) OR C HP	16	4	5	5	0.9250	0.8459	0.9652	0.5496	0.4642	0.6322
(A AND B) OR C HP	16	4	5	6	0.8875	0.7998	0.9397	0.6794	0.5953	0.7532
(A AND B) OR C HP	16	4	6	1	0.9875	0.9325	0.9978	0.2519	0.1854	0.3326
(A AND B) OR C HP	16	4	6	2	0.9875	0.9325	0.9978	0.3511	0.2747	0.4361
(A AND B) OR C HP	16	4	6	3	0.9500	0.8784	0.9804	0.4504	0.3678	0.5358
(A AND B) OR C HP	16	4	6	4	0.9500	0.8784	0.9804	0.4962	0.4119	0.5807

		Threshold				95% CI			95% CI	
Combination	EK	A	B	C	Sensitivity	Lower	Upper	Specificity	Lower	Upper
(A AND B) OR C HP	16	4	6	5	0.9250	0.8459	0.9652	0.5573	0.4718	0.6395
(A AND B) OR C HP	16	4	6	6	0.8750	0.7850	0.9307	0.6870	0.6032	0.7601
(A AND B) OR C HP	16	5	1	1	0.9875	0.9325	0.9978	0.2366	0.1720	0.3163
(A AND B) OR C HP	16	5	1	2	0.9875	0.9325	0.9978	0.3130	0.2399	0.3968
(A AND B) OR C HP	16	5	1	3	0.9500	0.8784	0.9804	0.4046	0.3244	0.4902
(A AND B) OR C HP	16	5	1	4	0.9500	0.8784	0.9804	0.4504	0.3678	0.5358
(A AND B) OR C HP	16	5	1	5	0.9250	0.8459	0.9652	0.4962	0.4119	0.5807
(A AND B) OR C HP	16	5	1	6	0.8750	0.7850	0.9307	0.6183	0.5329	0.6970
(A AND B) OR C HP	16	5	2	1	0.9875	0.9325	0.9978	0.2443	0.1787	0.3244
(A AND B) OR C HP	16	5	2	2	0.9875	0.9325	0.9978	0.3359	0.2607	0.4204
(A AND B) OR C HP	16	5	2	3	0.9500	0.8784	0.9804	0.4275	0.3460	0.5131
(A AND B) OR C HP	16	5	2	4	0.9500	0.8784	0.9804	0.4733	0.3898	0.5583
(A AND B) OR C HP	16	5	2	5	0.9250	0.8459	0.9652	0.5191	0.4342	0.6029
(A AND B) OR C HP	16	5	2	6	0.8750	0.7850	0.9307	0.6412	0.5561	0.7183
(A AND B) OR C HP	16	5	3	1	0.9875	0.9325	0.9978	0.2443	0.1787	0.3244
(A AND B) OR C HP	16	5	3	2	0.9875	0.9325	0.9978	0.3435	0.2677	0.4282
(A AND B) OR C HP	16	5	3	3	0.9500	0.8784	0.9804	0.4427	0.3605	0.5282
(A AND B) OR C HP	16	5	3	4	0.9500	0.8784	0.9804	0.4885	0.4045	0.5732
(A AND B) OR C HP	16	5	3	5	0.9250	0.8459	0.9652	0.5420	0.4567	0.6249
(A AND B) OR C HP	16	5	3	6	0.8750	0.7850	0.9307	0.6718	0.5875	0.7463
(A AND B) OR C HP	16	5	4	1	0.9875	0.9325	0.9978	0.2519	0.1854	0.3326
(A AND B) OR C HP	16	5	4	2	0.9875	0.9325	0.9978	0.3511	0.2747	0.4361
(A AND B) OR C HP	16	5	4	3	0.9500	0.8784	0.9804	0.4504	0.3678	0.5358
(A AND B) OR C HP	16	5	4	4	0.9500	0.8784	0.9804	0.4962	0.4119	0.5807
(A AND B) OR C HP	16	5	4	5	0.9250	0.8459	0.9652	0.5496	0.4642	0.6322
(A AND B) OR C HP	16	5	4	6	0.8750	0.7850	0.9307	0.6794	0.5953	0.7532
(A AND B) OR C HP	16	5	5	1	0.9875	0.9325	0.9978	0.2519	0.1854	0.3326
(A AND B) OR C HP	16	5	5	2	0.9875	0.9325	0.9978	0.3511	0.2747	0.4361
(A AND B) OR C HP	16	5	5	3	0.9500	0.8784	0.9804	0.4504	0.3678	0.5358
(A AND B) OR C HP	16	5	5	4	0.9500	0.8784	0.9804	0.4962	0.4119	0.5807
(A AND B) OR C HP	16	5	5	5	0.9250	0.8459	0.9652	0.5496	0.4642	0.6322
(A AND B) OR C HP	16	5	5	6	0.8750	0.7850	0.9307	0.6794	0.5953	0.7532
(A AND B) OR C HP	16	5	6	1	0.9875	0.9325	0.9978	0.2519	0.1854	0.3326
(A AND B) OR C HP	16	5	6	2	0.9875	0.9325	0.9978	0.3511	0.2747	0.4361
(A AND B) OR C HP	16	5	6	3	0.9500	0.8784	0.9804	0.4504	0.3678	0.5358
(A AND B) OR C HP	16	5	6	4	0.9500	0.8784	0.9804	0.4962	0.4119	0.5807
(A AND B) OR C HP	16	5	6	5	0.9250	0.8459	0.9652	0.5573	0.4718	0.6395
(A AND B) OR C HP	16	5	6	6	0.8750	0.7850	0.9307	0.6870	0.6032	0.7601
(A AND B) OR C HP	16	6	1	1	0.9875	0.9325	0.9978	0.2366	0.1720	0.3163
(A AND B) OR C HP	16	6	1	2	0.9875	0.9325	0.9978	0.3206	0.2468	0.4047
(A AND B) OR C HP	16	6	1	3	0.9500	0.8784	0.9804	0.4198	0.3388	0.5055
(A AND B) OR C HP	16	6	1	4	0.9500	0.8784	0.9804	0.4656	0.3824	0.5508
(A AND B) OR C HP	16	6	1	5	0.9250	0.8459	0.9652	0.5267	0.4417	0.6102
(A AND B) OR C HP	16	6	1	6	0.8625	0.7703	0.9215	0.6489	0.5639	0.7253
(A AND B) OR C HP	16	6	2	1	0.9875	0.9325	0.9978	0.2443	0.1787	0.3244
(A AND B) OR C HP	16	6	2	2	0.9875	0.9325	0.9978	0.3435	0.2677	0.4282
(A AND B) OR C HP	16	6	2	3	0.9500	0.8784	0.9804	0.4427	0.3605	0.5282
(A AND B) OR C HP	16	6	2	4	0.9500	0.8784	0.9804	0.4885	0.4045	0.5732
(A AND B) OR C HP	16	6	2	5	0.9250	0.8459	0.9652	0.5496	0.4642	0.6322
(A AND B) OR C HP	16	6	2	6	0.8625	0.7703	0.9215	0.6718	0.5875	0.7463
(A AND B) OR C HP	16	6	3	1	0.9875	0.9325	0.9978	0.2443	0.1787	0.3244
(A AND B) OR C HP	16	6	3	2	0.9875	0.9325	0.9978	0.3511	0.2747	0.4361
(A AND B) OR C HP	16	6	3	3	0.9500	0.8784	0.9804	0.4580	0.3751	0.5433

Combination	EK	Threshold A	B	C	Sensitivity	95% CI Lower	Upper	Specificity	95% CI Lower	Upper
(A AND B) OR C HP	16	6	3	4	0.9500	0.8784	0.9804	0.5038	0.4193	0.5881
(A AND B) OR C HP	16	6	3	5	0.9250	0.8459	0.9652	0.5649	0.4793	0.6467
(A AND B) OR C HP	16	6	3	6	0.8625	0.7703	0.9215	0.6947	0.6112	0.7670
(A AND B) OR C HP	16	6	4	1	0.9875	0.9325	0.9978	0.2519	0.1854	0.3326
(A AND B) OR C HP	16	6	4	2	0.9875	0.9325	0.9978	0.3588	0.2817	0.4439
(A AND B) OR C HP	16	6	4	3	0.9500	0.8784	0.9804	0.4656	0.3824	0.5508
(A AND B) OR C HP	16	6	4	4	0.9500	0.8784	0.9804	0.5115	0.4268	0.5955
(A AND B) OR C HP	16	6	4	5	0.9250	0.8459	0.9652	0.5725	0.4869	0.6540
(A AND B) OR C HP	16	6	4	6	0.8625	0.7703	0.9215	0.7023	0.6191	0.7739
(A AND B) OR C HP	16	6	5	1	0.9875	0.9325	0.9978	0.2519	0.1854	0.3326
(A AND B) OR C HP	16	6	5	2	0.9875	0.9325	0.9978	0.3588	0.2817	0.4439
(A AND B) OR C HP	16	6	5	3	0.9500	0.8784	0.9804	0.4656	0.3824	0.5508
(A AND B) OR C HP	16	6	5	4	0.9500	0.8784	0.9804	0.5115	0.4268	0.5955
(A AND B) OR C HP	16	6	5	5	0.9250	0.8459	0.9652	0.5725	0.4869	0.6540
(A AND B) OR C HP	16	6	5	6	0.8625	0.7703	0.9215	0.7023	0.6191	0.7739
(A AND B) OR C HP	16	6	6	1	0.9875	0.9325	0.9978	0.2519	0.1854	0.3326
(A AND B) OR C HP	16	6	6	2	0.9875	0.9325	0.9978	0.3588	0.2817	0.4439
(A AND B) OR C HP	16	6	6	3	0.9500	0.8784	0.9804	0.4656	0.3824	0.5508
(A AND B) OR C HP	16	6	6	4	0.9500	0.8784	0.9804	0.5115	0.4268	0.5955
(A AND B) OR C HP	16	6	6	5	0.9250	0.8459	0.9652	0.5725	0.4869	0.6540
(A AND B) OR C HP	16	6	6	6	0.8625	0.7703	0.9215	0.7023	0.6191	0.7739
(B AND C) OR A HP	17	1	1	1	0.9875	0.9325	0.9978	0.0992	0.0589	0.1624
(B AND C) OR A HP	17	1	1	2	0.9875	0.9325	0.9978	0.0992	0.0589	0.1624
(B AND C) OR A HP	17	1	1	3	0.9750	0.9134	0.9931	0.1298	0.0826	0.1980
(B AND C) OR A HP	17	1	1	4	0.9750	0.9134	0.9931	0.1298	0.0826	0.1980
(B AND C) OR A HP	17	1	1	5	0.9625	0.8955	0.9872	0.1374	0.0887	0.2068
(B AND C) OR A HP	17	1	1	6	0.9500	0.8784	0.9804	0.1527	0.1011	0.2241
(B AND C) OR A HP	17	1	2	1	0.9625	0.8955	0.9872	0.1145	0.0706	0.1803
(B AND C) OR A HP	17	1	2	2	0.9625	0.8955	0.9872	0.1145	0.0706	0.1803
(B AND C) OR A HP	17	1	2	3	0.9500	0.8784	0.9804	0.1374	0.0887	0.2068
(B AND C) OR A HP	17	1	2	4	0.9500	0.8784	0.9804	0.1374	0.0887	0.2068
(B AND C) OR A HP	17	1	2	5	0.9500	0.8784	0.9804	0.1450	0.0949	0.2154
(B AND C) OR A HP	17	1	2	6	0.9375	0.8619	0.9730	0.1603	0.1073	0.2327
(B AND C) OR A HP	17	1	3	1	0.9625	0.8955	0.9872	0.1374	0.0887	0.2068
(B AND C) OR A HP	17	1	3	2	0.9625	0.8955	0.9872	0.1374	0.0887	0.2068
(B AND C) OR A HP	17	1	3	3	0.9500	0.8784	0.9804	0.1527	0.1011	0.2241
(B AND C) OR A HP	17	1	3	4	0.9500	0.8784	0.9804	0.1527	0.1011	0.2241
(B AND C) OR A HP	17	1	3	5	0.9500	0.8784	0.9804	0.1603	0.1073	0.2327
(B AND C) OR A HP	17	1	3	6	0.9375	0.8619	0.9730	0.1679	0.1136	0.2412
(B AND C) OR A HP	17	1	4	1	0.9500	0.8784	0.9804	0.1527	0.1011	0.2241
(B AND C) OR A HP	17	1	4	2	0.9500	0.8784	0.9804	0.1527	0.1011	0.2241
(B AND C) OR A HP	17	1	4	3	0.9500	0.8784	0.9804	0.1527	0.1011	0.2241
(B AND C) OR A HP	17	1	4	4	0.9500	0.8784	0.9804	0.1527	0.1011	0.2241
(B AND C) OR A HP	17	1	4	5	0.9500	0.8784	0.9804	0.1603	0.1073	0.2327
(B AND C) OR A HP	17	1	4	6	0.9375	0.8619	0.9730	0.1679	0.1136	0.2412
(B AND C) OR A HP	17	1	5	1	0.9375	0.8619	0.9730	0.1527	0.1011	0.2241
(B AND C) OR A HP	17	1	5	2	0.9375	0.8619	0.9730	0.1527	0.1011	0.2241
(B AND C) OR A HP	17	1	5	3	0.9375	0.8619	0.9730	0.1527	0.1011	0.2241
(B AND C) OR A HP	17	1	5	4	0.9375	0.8619	0.9730	0.1527	0.1011	0.2241
(B AND C) OR A HP	17	1	5	5	0.9375	0.8619	0.9730	0.1603	0.1073	0.2327
(B AND C) OR A HP	17	1	5	6	0.9375	0.8619	0.9730	0.1679	0.1136	0.2412
(B AND C) OR A HP	17	1	6	1	0.9250	0.8459	0.9652	0.1603	0.1073	0.2327
(B AND C) OR A HP	17	1	6	2	0.9250	0.8459	0.9652	0.1603	0.1073	0.2327

		Threshold				95% CI			95% CI	
Combination	EK	A	B	C	Sensitivity	Lower	Upper	Specificity	Lower	Upper
(B AND C) OR A HP	17	1	6	3	0.9250	0.8459	0.9652	0.1603	0.1073	0.2327
(B AND C) OR A HP	17	1	6	4	0.9250	0.8459	0.9652	0.1603	0.1073	0.2327
(B AND C) OR A HP	17	1	6	5	0.9250	0.8459	0.9652	0.1603	0.1073	0.2327
(B AND C) OR A HP	17	1	6	6	0.9250	0.8459	0.9652	0.1679	0.1136	0.2412
(B AND C) OR A HP	17	2	1	1	0.9750	0.9134	0.9931	0.1832	0.1263	0.2581
(B AND C) OR A HP	17	2	1	2	0.9750	0.9134	0.9931	0.1985	0.1392	0.2749
(B AND C) OR A HP	17	2	1	3	0.9625	0.8955	0.9872	0.2290	0.1654	0.3081
(B AND C) OR A HP	17	2	1	4	0.9625	0.8955	0.9872	0.2290	0.1654	0.3081
(B AND C) OR A HP	17	2	1	5	0.9500	0.8784	0.9804	0.2443	0.1787	0.3244
(B AND C) OR A HP	17	2	1	6	0.9250	0.8459	0.9652	0.2672	0.1988	0.3488
(B AND C) OR A HP	17	2	2	1	0.9500	0.8784	0.9804	0.2137	0.1522	0.2916
(B AND C) OR A HP	17	2	2	2	0.9500	0.8784	0.9804	0.2290	0.1654	0.3081
(B AND C) OR A HP	17	2	2	3	0.9375	0.8619	0.9730	0.2519	0.1854	0.3326
(B AND C) OR A HP	17	2	2	4	0.9375	0.8619	0.9730	0.2519	0.1854	0.3326
(B AND C) OR A HP	17	2	2	5	0.9375	0.8619	0.9730	0.2595	0.1921	0.3407
(B AND C) OR A HP	17	2	2	6	0.9125	0.8302	0.9570	0.2748	0.2056	0.3568
(B AND C) OR A HP	17	2	3	1	0.9500	0.8784	0.9804	0.2366	0.1720	0.3163
(B AND C) OR A HP	17	2	3	2	0.9500	0.8784	0.9804	0.2519	0.1854	0.3326
(B AND C) OR A HP	17	2	3	3	0.9375	0.8619	0.9730	0.2672	0.1988	0.3488
(B AND C) OR A HP	17	2	3	4	0.9375	0.8619	0.9730	0.2672	0.1988	0.3488
(B AND C) OR A HP	17	2	3	5	0.9375	0.8619	0.9730	0.2748	0.2056	0.3568
(B AND C) OR A HP	17	2	3	6	0.9125	0.8302	0.9570	0.2824	0.2124	0.3649
(B AND C) OR A HP	17	2	4	1	0.9375	0.8619	0.9730	0.2595	0.1921	0.3407
(B AND C) OR A HP	17	2	4	2	0.9375	0.8619	0.9730	0.2672	0.1988	0.3488
(B AND C) OR A HP	17	2	4	3	0.9375	0.8619	0.9730	0.2672	0.1988	0.3488
(B AND C) OR A HP	17	2	4	4	0.9375	0.8619	0.9730	0.2672	0.1988	0.3488
(B AND C) OR A HP	17	2	4	5	0.9375	0.8619	0.9730	0.2748	0.2056	0.3568
(B AND C) OR A HP	17	2	4	6	0.9125	0.8302	0.9570	0.2824	0.2124	0.3649
(B AND C) OR A HP	17	2	5	1	0.9250	0.8459	0.9652	0.2672	0.1988	0.3488
(B AND C) OR A HP	17	2	5	2	0.9250	0.8459	0.9652	0.2672	0.1988	0.3488
(B AND C) OR A HP	17	2	5	3	0.9250	0.8459	0.9652	0.2672	0.1988	0.3488
(B AND C) OR A HP	17	2	5	4	0.9250	0.8459	0.9652	0.2672	0.1988	0.3488
(B AND C) OR A HP	17	2	5	5	0.9250	0.8459	0.9652	0.2748	0.2056	0.3568
(B AND C) OR A HP	17	2	5	6	0.9125	0.8302	0.9570	0.2824	0.2124	0.3649
(B AND C) OR A HP	17	2	6	1	0.9125	0.8302	0.9570	0.2748	0.2056	0.3568
(B AND C) OR A HP	17	2	6	2	0.9125	0.8302	0.9570	0.2748	0.2056	0.3568
(B AND C) OR A HP	17	2	6	3	0.9125	0.8302	0.9570	0.2748	0.2056	0.3568
(B AND C) OR A HP	17	2	6	4	0.9125	0.8302	0.9570	0.2748	0.2056	0.3568
(B AND C) OR A HP	17	2	6	5	0.9125	0.8302	0.9570	0.2748	0.2056	0.3568
(B AND C) OR A HP	17	2	6	6	0.9000	0.8149	0.9485	0.2824	0.2124	0.3649
(B AND C) OR A HP	17	3	1	1	0.9625	0.8955	0.9872	0.2443	0.1787	0.3244
(B AND C) OR A HP	17	3	1	2	0.9625	0.8955	0.9872	0.2672	0.1988	0.3488
(B AND C) OR A HP	17	3	1	3	0.9500	0.8784	0.9804	0.2977	0.2261	0.3809
(B AND C) OR A HP	17	3	1	4	0.9500	0.8784	0.9804	0.3053	0.2330	0.3888
(B AND C) OR A HP	17	3	1	5	0.9375	0.8619	0.9730	0.3359	0.2607	0.4204
(B AND C) OR A HP	17	3	1	6	0.9125	0.8302	0.9570	0.3740	0.2959	0.4594
(B AND C) OR A HP	17	3	2	1	0.9375	0.8619	0.9730	0.2977	0.2261	0.3809
(B AND C) OR A HP	17	3	2	2	0.9375	0.8619	0.9730	0.3206	0.2468	0.4047
(B AND C) OR A HP	17	3	2	3	0.9250	0.8459	0.9652	0.3435	0.2677	0.4282
(B AND C) OR A HP	17	3	2	4	0.9250	0.8459	0.9652	0.3511	0.2747	0.4361
(B AND C) OR A HP	17	3	2	5	0.9250	0.8459	0.9652	0.3740	0.2959	0.4594
(B AND C) OR A HP	17	3	2	6	0.9000	0.8149	0.9485	0.3969	0.3172	0.4825
(B AND C) OR A HP	17	3	3	1	0.9375	0.8619	0.9730	0.3435	0.2677	0.4282

		Threshold				95% CI			95% CI	
Combination	EK	A	B	C	Sensitivity	Lower	Upper	Specificity	Lower	Upper
(B AND C) OR A HP	17	3	3	2	0.9375	0.8619	0.9730	0.3664	0.2888	0.4516
(B AND C) OR A HP	17	3	3	3	0.9250	0.8459	0.9652	0.3817	0.3030	0.4671
(B AND C) OR A HP	17	3	3	4	0.9250	0.8459	0.9652	0.3817	0.3030	0.4671
(B AND C) OR A HP	17	3	3	5	0.9250	0.8459	0.9652	0.4046	0.3244	0.4902
(B AND C) OR A HP	17	3	3	6	0.9000	0.8149	0.9485	0.4198	0.3388	0.5055
(B AND C) OR A HP	17	3	4	1	0.9250	0.8459	0.9652	0.3893	0.3101	0.4748
(B AND C) OR A HP	17	3	4	2	0.9250	0.8459	0.9652	0.3969	0.3172	0.4825
(B AND C) OR A HP	17	3	4	3	0.9250	0.8459	0.9652	0.3969	0.3172	0.4825
(B AND C) OR A HP	17	3	4	4	0.9250	0.8459	0.9652	0.3969	0.3172	0.4825
(B AND C) OR A HP	17	3	4	5	0.9250	0.8459	0.9652	0.4046	0.3244	0.4902
(B AND C) OR A HP	17	3	4	6	0.9000	0.8149	0.9485	0.4198	0.3388	0.5055
(B AND C) OR A HP	17	3	5	1	0.9125	0.8302	0.9570	0.4122	0.3316	0.4978
(B AND C) OR A HP	17	3	5	2	0.9125	0.8302	0.9570	0.4122	0.3316	0.4978
(B AND C) OR A HP	17	3	5	3	0.9125	0.8302	0.9570	0.4122	0.3316	0.4978
(B AND C) OR A HP	17	3	5	4	0.9125	0.8302	0.9570	0.4122	0.3316	0.4978
(B AND C) OR A HP	17	3	5	5	0.9125	0.8302	0.9570	0.4198	0.3388	0.5055
(B AND C) OR A HP	17	3	5	6	0.9000	0.8149	0.9485	0.4275	0.3460	0.5131
(B AND C) OR A HP	17	3	6	1	0.9000	0.8149	0.9485	0.4198	0.3388	0.5055
(B AND C) OR A HP	17	3	6	2	0.9000	0.8149	0.9485	0.4198	0.3388	0.5055
(B AND C) OR A HP	17	3	6	3	0.9000	0.8149	0.9485	0.4198	0.3388	0.5055
(B AND C) OR A HP	17	3	6	4	0.9000	0.8149	0.9485	0.4198	0.3388	0.5055
(B AND C) OR A HP	17	3	6	5	0.9000	0.8149	0.9485	0.4198	0.3388	0.5055
(B AND C) OR A HP	17	3	6	6	0.8875	0.7998	0.9397	0.4275	0.3460	0.5131
(B AND C) OR A HP	17	4	1	1	0.9500	0.8784	0.9804	0.3282	0.2537	0.4125
(B AND C) OR A HP	17	4	1	2	0.9500	0.8784	0.9804	0.3511	0.2747	0.4361
(B AND C) OR A HP	17	4	1	3	0.9375	0.8619	0.9730	0.3817	0.3030	0.4671
(B AND C) OR A HP	17	4	1	4	0.9375	0.8619	0.9730	0.4046	0.3244	0.4902
(B AND C) OR A HP	17	4	1	5	0.9125	0.8302	0.9570	0.4351	0.3533	0.5207
(B AND C) OR A HP	17	4	1	6	0.8750	0.7850	0.9307	0.4885	0.4045	0.5732
(B AND C) OR A HP	17	4	2	1	0.9250	0.8459	0.9652	0.3893	0.3101	0.4748
(B AND C) OR A HP	17	4	2	2	0.9250	0.8459	0.9652	0.4122	0.3316	0.4978
(B AND C) OR A HP	17	4	2	3	0.9125	0.8302	0.9570	0.4351	0.3533	0.5207
(B AND C) OR A HP	17	4	2	4	0.9125	0.8302	0.9570	0.4504	0.3678	0.5358
(B AND C) OR A HP	17	4	2	5	0.9000	0.8149	0.9485	0.4733	0.3898	0.5583
(B AND C) OR A HP	17	4	2	6	0.8625	0.7703	0.9215	0.5115	0.4268	0.5955
(B AND C) OR A HP	17	4	3	1	0.9250	0.8459	0.9652	0.4504	0.3678	0.5358
(B AND C) OR A HP	17	4	3	2	0.9250	0.8459	0.9652	0.4733	0.3898	0.5583
(B AND C) OR A HP	17	4	3	3	0.9125	0.8302	0.9570	0.4885	0.4045	0.5732
(B AND C) OR A HP	17	4	3	4	0.9125	0.8302	0.9570	0.4962	0.4119	0.5807
(B AND C) OR A HP	17	4	3	5	0.9000	0.8149	0.9485	0.5191	0.4342	0.6029
(B AND C) OR A HP	17	4	3	6	0.8625	0.7703	0.9215	0.5496	0.4642	0.6322
(B AND C) OR A HP	17	4	4	1	0.8875	0.7998	0.9397	0.5267	0.4417	0.6102
(B AND C) OR A HP	17	4	4	2	0.8875	0.7998	0.9397	0.5344	0.4492	0.6176
(B AND C) OR A HP	17	4	4	3	0.8875	0.7998	0.9397	0.5344	0.4492	0.6176
(B AND C) OR A HP	17	4	4	4	0.8875	0.7998	0.9397	0.5344	0.4492	0.6176
(B AND C) OR A HP	17	4	4	5	0.8875	0.7998	0.9397	0.5420	0.4567	0.6249
(B AND C) OR A HP	17	4	4	6	0.8625	0.7703	0.9215	0.5649	0.4793	0.6467
(B AND C) OR A HP	17	4	5	1	0.8750	0.7850	0.9307	0.5496	0.4642	0.6322
(B AND C) OR A HP	17	4	5	2	0.8750	0.7850	0.9307	0.5496	0.4642	0.6322
(B AND C) OR A HP	17	4	5	3	0.8750	0.7850	0.9307	0.5496	0.4642	0.6322
(B AND C) OR A HP	17	4	5	4	0.8750	0.7850	0.9307	0.5496	0.4642	0.6322
(B AND C) OR A HP	17	4	5	5	0.8750	0.7850	0.9307	0.5573	0.4718	0.6395
(B AND C) OR A HP	17	4	5	6	0.8625	0.7703	0.9215	0.5725	0.4869	0.6540

		Threshold				95% CI			95% CI	
Combination	EK	A	B	C	Sensitivity	Lower	Upper	Specificity	Lower	Upper
(B AND C) OR A HP	17	4	6	1	0.8625	0.7703	0.9215	0.5573	0.4718	0.6395
(B AND C) OR A HP	17	4	6	2	0.8625	0.7703	0.9215	0.5573	0.4718	0.6395
(B AND C) OR A HP	17	4	6	3	0.8625	0.7703	0.9215	0.5573	0.4718	0.6395
(B AND C) OR A HP	17	4	6	4	0.8625	0.7703	0.9215	0.5573	0.4718	0.6395
(B AND C) OR A HP	17	4	6	5	0.8625	0.7703	0.9215	0.5573	0.4718	0.6395
(B AND C) OR A HP	17	4	6	6	0.8500	0.7559	0.9121	0.5725	0.4869	0.6540
(B AND C) OR A HP	17	5	1	1	0.9500	0.8784	0.9804	0.3817	0.3030	0.4671
(B AND C) OR A HP	17	5	1	2	0.9500	0.8784	0.9804	0.4046	0.3244	0.4902
(B AND C) OR A HP	17	5	1	3	0.9375	0.8619	0.9730	0.4351	0.3533	0.5207
(B AND C) OR A HP	17	5	1	4	0.9375	0.8619	0.9730	0.4580	0.3751	0.5433
(B AND C) OR A HP	17	5	1	5	0.9125	0.8302	0.9570	0.4885	0.4045	0.5732
(B AND C) OR A HP	17	5	1	6	0.8625	0.7703	0.9215	0.5649	0.4793	0.6467
(B AND C) OR A HP	17	5	2	1	0.9250	0.8459	0.9652	0.4580	0.3751	0.5433
(B AND C) OR A HP	17	5	2	2	0.9250	0.8459	0.9652	0.4809	0.3971	0.5658
(B AND C) OR A HP	17	5	2	3	0.9125	0.8302	0.9570	0.5038	0.4193	0.5881
(B AND C) OR A HP	17	5	2	4	0.9125	0.8302	0.9570	0.5191	0.4342	0.6029
(B AND C) OR A HP	17	5	2	5	0.9000	0.8149	0.9485	0.5420	0.4567	0.6249
(B AND C) OR A HP	17	5	2	6	0.8500	0.7559	0.9121	0.5954	0.5098	0.6756
(B AND C) OR A HP	17	5	3	1	0.9250	0.8459	0.9652	0.5420	0.4567	0.6249
(B AND C) OR A HP	17	5	3	2	0.9250	0.8459	0.9652	0.5649	0.4793	0.6467
(B AND C) OR A HP	17	5	3	3	0.9125	0.8302	0.9570	0.5802	0.4945	0.6612
(B AND C) OR A HP	17	5	3	4	0.9125	0.8302	0.9570	0.5878	0.5022	0.6684
(B AND C) OR A HP	17	5	3	5	0.9000	0.8149	0.9485	0.6107	0.5252	0.6899
(B AND C) OR A HP	17	5	3	6	0.8500	0.7559	0.9121	0.6489	0.5639	0.7253
(B AND C) OR A HP	17	5	4	1	0.8750	0.7850	0.9307	0.6260	0.5406	0.7041
(B AND C) OR A HP	17	5	4	2	0.8750	0.7850	0.9307	0.6336	0.5484	0.7112
(B AND C) OR A HP	17	5	4	3	0.8750	0.7850	0.9307	0.6336	0.5484	0.7112
(B AND C) OR A HP	17	5	4	4	0.8750	0.7850	0.9307	0.6336	0.5484	0.7112
(B AND C) OR A HP	17	5	4	5	0.8750	0.7850	0.9307	0.6412	0.5561	0.7183
(B AND C) OR A HP	17	5	4	6	0.8375	0.7416	0.9025	0.6641	0.5796	0.7393
(B AND C) OR A HP	17	5	5	1	0.8625	0.7703	0.9215	0.6489	0.5639	0.7253
(B AND C) OR A HP	17	5	5	2	0.8625	0.7703	0.9215	0.6489	0.5639	0.7253
(B AND C) OR A HP	17	5	5	3	0.8625	0.7703	0.9215	0.6489	0.5639	0.7253
(B AND C) OR A HP	17	5	5	4	0.8625	0.7703	0.9215	0.6489	0.5639	0.7253
(B AND C) OR A HP	17	5	5	5	0.8625	0.7703	0.9215	0.6565	0.5718	0.7323
(B AND C) OR A HP	17	5	5	6	0.8375	0.7416	0.9025	0.6718	0.5875	0.7463
(B AND C) OR A HP	17	5	6	1	0.8375	0.7416	0.9025	0.6565	0.5718	0.7323
(B AND C) OR A HP	17	5	6	2	0.8375	0.7416	0.9025	0.6565	0.5718	0.7323
(B AND C) OR A HP	17	5	6	3	0.8375	0.7416	0.9025	0.6565	0.5718	0.7323
(B AND C) OR A HP	17	5	6	4	0.8375	0.7416	0.9025	0.6565	0.5718	0.7323
(B AND C) OR A HP	17	5	6	5	0.8375	0.7416	0.9025	0.6565	0.5718	0.7323
(B AND C) OR A HP	17	5	6	6	0.8250	0.7274	0.8928	0.6718	0.5875	0.7463
(B AND C) OR A HP	17	6	1	1	0.9500	0.8784	0.9804	0.4198	0.3388	0.5055
(B AND C) OR A HP	17	6	1	2	0.9500	0.8784	0.9804	0.4504	0.3678	0.5358
(B AND C) OR A HP	17	6	1	3	0.9375	0.8619	0.9730	0.4885	0.4045	0.5732
(B AND C) OR A HP	17	6	1	4	0.9375	0.8619	0.9730	0.5115	0.4268	0.5955
(B AND C) OR A HP	17	6	1	5	0.9125	0.8302	0.9570	0.5573	0.4718	0.6395
(B AND C) OR A HP	17	6	1	6	0.8500	0.7559	0.9121	0.6336	0.5484	0.7112
(B AND C) OR A HP	17	6	2	1	0.9250	0.8459	0.9652	0.4962	0.4119	0.5807
(B AND C) OR A HP	17	6	2	2	0.9250	0.8459	0.9652	0.5267	0.4417	0.6102
(B AND C) OR A HP	17	6	2	3	0.9125	0.8302	0.9570	0.5573	0.4718	0.6395
(B AND C) OR A HP	17	6	2	4	0.9125	0.8302	0.9570	0.5725	0.4869	0.6540
(B AND C) OR A HP	17	6	2	5	0.9000	0.8149	0.9485	0.6107	0.5252	0.6899

		Threshold				95% CI			95% CI	
Combination	EK	A	B	C	Sensitivity	Lower	Upper	Specificity	Lower	Upper
(B AND C) OR A HP	17	6	2	6	0.8375	0.7416	0.9025	0.6641	0.5796	0.7393
(B AND C) OR A HP	17	6	3	1	0.9250	0.8459	0.9652	0.5954	0.5098	0.6756
(B AND C) OR A HP	17	6	3	2	0.9250	0.8459	0.9652	0.6260	0.5406	0.7041
(B AND C) OR A HP	17	6	3	3	0.9125	0.8302	0.9570	0.6489	0.5639	0.7253
(B AND C) OR A HP	17	6	3	4	0.9125	0.8302	0.9570	0.6565	0.5718	0.7323
(B AND C) OR A HP	17	6	3	5	0.9000	0.8149	0.9485	0.6870	0.6032	0.7601
(B AND C) OR A HP	17	6	3	6	0.8375	0.7416	0.9025	0.7252	0.6432	0.7944
(B AND C) OR A HP	17	6	4	1	0.8750	0.7850	0.9307	0.6794	0.5953	0.7532
(B AND C) OR A HP	17	6	4	2	0.8750	0.7850	0.9307	0.6947	0.6112	0.7670
(B AND C) OR A HP	17	6	4	3	0.8750	0.7850	0.9307	0.7023	0.6191	0.7739
(B AND C) OR A HP	17	6	4	4	0.8750	0.7850	0.9307	0.7023	0.6191	0.7739
(B AND C) OR A HP	17	6	4	5	0.8750	0.7850	0.9307	0.7176	0.6351	0.7876
(B AND C) OR A HP	17	6	4	6	0.8250	0.7274	0.8928	0.7405	0.6593	0.8079
(B AND C) OR A HP	17	6	5	1	0.8625	0.7703	0.9215	0.7023	0.6191	0.7739
(B AND C) OR A HP	17	6	5	2	0.8625	0.7703	0.9215	0.7099	0.6271	0.7808
(B AND C) OR A HP	17	6	5	3	0.8625	0.7703	0.9215	0.7176	0.6351	0.7876
(B AND C) OR A HP	17	6	5	4	0.8625	0.7703	0.9215	0.7176	0.6351	0.7876
(B AND C) OR A HP	17	6	5	5	0.8625	0.7703	0.9215	0.7328	0.6512	0.8012
(B AND C) OR A HP	17	6	5	6	0.8250	0.7274	0.8928	0.7481	0.6674	0.8146
(B AND C) OR A HP	17	6	6	1	0.8375	0.7416	0.9025	0.7176	0.6351	0.7876
(B AND C) OR A HP	17	6	6	2	0.8375	0.7416	0.9025	0.7252	0.6432	0.7944
(B AND C) OR A HP	17	6	6	3	0.8375	0.7416	0.9025	0.7328	0.6512	0.8012
(B AND C) OR A HP	17	6	6	4	0.8375	0.7416	0.9025	0.7328	0.6512	0.8012
(B AND C) OR A HP	17	6	6	5	0.8375	0.7416	0.9025	0.7328	0.6512	0.8012
(B AND C) OR A HP	17	6	6	6	0.8125	0.7134	0.8829	0.7481	0.6674	0.8146
(A AND C) OR B HP	18	1	1	1	0.9750	0.9134	0.9931	0.1756	0.1199	0.2497
(A AND C) OR B HP	18	1	1	2	0.9750	0.9134	0.9931	0.2290	0.1654	0.3081
(A AND C) OR B HP	18	1	1	3	0.9500	0.8784	0.9804	0.2824	0.2124	0.3649
(A AND C) OR B HP	18	1	1	4	0.9500	0.8784	0.9804	0.3053	0.2330	0.3888
(A AND C) OR B HP	18	1	1	5	0.9500	0.8784	0.9804	0.3206	0.2468	0.4047
(A AND C) OR B HP	18	1	1	6	0.9500	0.8784	0.9804	0.3359	0.2607	0.4204
(A AND C) OR B HP	18	1	2	1	0.9500	0.8784	0.9804	0.2214	0.1588	0.2998
(A AND C) OR B HP	18	1	2	2	0.9500	0.8784	0.9804	0.2901	0.2192	0.3729
(A AND C) OR B HP	18	1	2	3	0.9250	0.8459	0.9652	0.3435	0.2677	0.4282
(A AND C) OR B HP	18	1	2	4	0.9250	0.8459	0.9652	0.3740	0.2959	0.4594
(A AND C) OR B HP	18	1	2	5	0.9250	0.8459	0.9652	0.3969	0.3172	0.4825
(A AND C) OR B HP	18	1	2	6	0.9250	0.8459	0.9652	0.4351	0.3533	0.5207
(A AND C) OR B HP	18	1	3	1	0.9500	0.8784	0.9804	0.2595	0.1921	0.3407
(A AND C) OR B HP	18	1	3	2	0.9500	0.8784	0.9804	0.3359	0.2607	0.4204
(A AND C) OR B HP	18	1	3	3	0.9250	0.8459	0.9652	0.3969	0.3172	0.4825
(A AND C) OR B HP	18	1	3	4	0.9250	0.8459	0.9652	0.4351	0.3533	0.5207
(A AND C) OR B HP	18	1	3	5	0.9250	0.8459	0.9652	0.4656	0.3824	0.5508
(A AND C) OR B HP	18	1	3	6	0.9250	0.8459	0.9652	0.5191	0.4342	0.6029
(A AND C) OR B HP	18	1	4	1	0.9375	0.8619	0.9730	0.3130	0.2399	0.3968
(A AND C) OR B HP	18	1	4	2	0.9375	0.8619	0.9730	0.4046	0.3244	0.4902
(A AND C) OR B HP	18	1	4	3	0.9125	0.8302	0.9570	0.4656	0.3824	0.5508
(A AND C) OR B HP	18	1	4	4	0.9125	0.8302	0.9570	0.5115	0.4268	0.5955
(A AND C) OR B HP	18	1	4	5	0.9000	0.8149	0.9485	0.5573	0.4718	0.6395
(A AND C) OR B HP	18	1	4	6	0.8875	0.7998	0.9397	0.6260	0.5406	0.7041
(A AND C) OR B HP	18	1	5	1	0.9250	0.8459	0.9652	0.3206	0.2468	0.4047
(A AND C) OR B HP	18	1	5	2	0.9250	0.8459	0.9652	0.4198	0.3388	0.5055
(A AND C) OR B HP	18	1	5	3	0.9000	0.8149	0.9485	0.4809	0.3971	0.5658
(A AND C) OR B HP	18	1	5	4	0.9000	0.8149	0.9485	0.5267	0.4417	0.6102

		Threshold				95% CI			95% CI	
Combination	EK	A	B	C	Sensitivity	Lower	Upper	Specificity	Lower	Upper
(A AND C) OR B HP	18	1	5	5	0.8875	0.7998	0.9397	0.5725	0.4869	0.6540
(A AND C) OR B HP	18	1	5	6	0.8750	0.7850	0.9307	0.6489	0.5639	0.7253
(A AND C) OR B HP	18	1	6	1	0.9125	0.8302	0.9570	0.3435	0.2677	0.4282
(A AND C) OR B HP	18	1	6	2	0.9125	0.8302	0.9570	0.4427	0.3605	0.5282
(A AND C) OR B HP	18	1	6	3	0.8875	0.7998	0.9397	0.5038	0.4193	0.5881
(A AND C) OR B HP	18	1	6	4	0.8875	0.7998	0.9397	0.5496	0.4642	0.6322
(A AND C) OR B HP	18	1	6	5	0.8750	0.7850	0.9307	0.6031	0.5175	0.6828
(A AND C) OR B HP	18	1	6	6	0.8500	0.7559	0.9121	0.6794	0.5953	0.7532
(A AND C) OR B HP	18	2	1	1	0.9625	0.8955	0.9872	0.2061	0.1457	0.2833
(A AND C) OR B HP	18	2	1	2	0.9625	0.8955	0.9872	0.2519	0.1854	0.3326
(A AND C) OR B HP	18	2	1	3	0.9500	0.8784	0.9804	0.3053	0.2330	0.3888
(A AND C) OR B HP	18	2	1	4	0.9500	0.8784	0.9804	0.3282	0.2537	0.4125
(A AND C) OR B HP	18	2	1	5	0.9500	0.8784	0.9804	0.3359	0.2607	0.4204
(A AND C) OR B HP	18	2	1	6	0.9500	0.8784	0.9804	0.3511	0.2747	0.4361
(A AND C) OR B HP	18	2	2	1	0.9375	0.8619	0.9730	0.2672	0.1988	0.3488
(A AND C) OR B HP	18	2	2	2	0.9375	0.8619	0.9730	0.3282	0.2537	0.4125
(A AND C) OR B HP	18	2	2	3	0.9250	0.8459	0.9652	0.3817	0.3030	0.4671
(A AND C) OR B HP	18	2	2	4	0.9250	0.8459	0.9652	0.4122	0.3316	0.4978
(A AND C) OR B HP	18	2	2	5	0.9250	0.8459	0.9652	0.4198	0.3388	0.5055
(A AND C) OR B HP	18	2	2	6	0.9250	0.8459	0.9652	0.4504	0.3678	0.5358
(A AND C) OR B HP	18	2	3	1	0.9375	0.8619	0.9730	0.3053	0.2330	0.3888
(A AND C) OR B HP	18	2	3	2	0.9375	0.8619	0.9730	0.3740	0.2959	0.4594
(A AND C) OR B HP	18	2	3	3	0.9250	0.8459	0.9652	0.4351	0.3533	0.5207
(A AND C) OR B HP	18	2	3	4	0.9250	0.8459	0.9652	0.4733	0.3898	0.5583
(A AND C) OR B HP	18	2	3	5	0.9250	0.8459	0.9652	0.4885	0.4045	0.5732
(A AND C) OR B HP	18	2	3	6	0.9250	0.8459	0.9652	0.5344	0.4492	0.6176
(A AND C) OR B HP	18	2	4	1	0.9250	0.8459	0.9652	0.3664	0.2888	0.4516
(A AND C) OR B HP	18	2	4	2	0.9250	0.8459	0.9652	0.4427	0.3605	0.5282
(A AND C) OR B HP	18	2	4	3	0.9125	0.8302	0.9570	0.5038	0.4193	0.5881
(A AND C) OR B HP	18	2	4	4	0.9125	0.8302	0.9570	0.5496	0.4642	0.6322
(A AND C) OR B HP	18	2	4	5	0.9000	0.8149	0.9485	0.5802	0.4945	0.6612
(A AND C) OR B HP	18	2	4	6	0.8875	0.7998	0.9397	0.6412	0.5561	0.7183
(A AND C) OR B HP	18	2	5	1	0.9125	0.8302	0.9570	0.3817	0.3030	0.4671
(A AND C) OR B HP	18	2	5	2	0.9125	0.8302	0.9570	0.4580	0.3751	0.5433
(A AND C) OR B HP	18	2	5	3	0.9000	0.8149	0.9485	0.5191	0.4342	0.6029
(A AND C) OR B HP	18	2	5	4	0.9000	0.8149	0.9485	0.5649	0.4793	0.6467
(A AND C) OR B HP	18	2	5	5	0.8875	0.7998	0.9397	0.5954	0.5098	0.6756
(A AND C) OR B HP	18	2	5	6	0.8750	0.7850	0.9307	0.6641	0.5796	0.7393
(A AND C) OR B HP	18	2	6	1	0.9000	0.8149	0.9485	0.4046	0.3244	0.4902
(A AND C) OR B HP	18	2	6	2	0.9000	0.8149	0.9485	0.4809	0.3971	0.5658
(A AND C) OR B HP	18	2	6	3	0.8875	0.7998	0.9397	0.5420	0.4567	0.6249
(A AND C) OR B HP	18	2	6	4	0.8875	0.7998	0.9397	0.5878	0.5022	0.6684
(A AND C) OR B HP	18	2	6	5	0.8750	0.7850	0.9307	0.6260	0.5406	0.7041
(A AND C) OR B HP	18	2	6	6	0.8500	0.7559	0.9121	0.6947	0.6112	0.7670
(A AND C) OR B HP	18	3	1	1	0.9625	0.8955	0.9872	0.2290	0.1654	0.3081
(A AND C) OR B HP	18	3	1	2	0.9625	0.8955	0.9872	0.2748	0.2056	0.3568
(A AND C) OR B HP	18	3	1	3	0.9500	0.8784	0.9804	0.3206	0.2468	0.4047
(A AND C) OR B HP	18	3	1	4	0.9500	0.8784	0.9804	0.3359	0.2607	0.4204
(A AND C) OR B HP	18	3	1	5	0.9500	0.8784	0.9804	0.3435	0.2677	0.4282
(A AND C) OR B HP	18	3	1	6	0.9500	0.8784	0.9804	0.3588	0.2817	0.4439
(A AND C) OR B HP	18	3	2	1	0.9375	0.8619	0.9730	0.3130	0.2399	0.3968
(A AND C) OR B HP	18	3	2	2	0.9375	0.8619	0.9730	0.3740	0.2959	0.4594
(A AND C) OR B HP	18	3	2	3	0.9250	0.8459	0.9652	0.4198	0.3388	0.5055

		Threshold				95% CI			95% CI	
Combination	EK	A	B	C	Sensitivity	Lower	Upper	Specificity	Lower	Upper
(A AND C) OR B HP	18	3	2	4	0.9250	0.8459	0.9652	0.4427	0.3605	0.5282
(A AND C) OR B HP	18	3	2	5	0.9250	0.8459	0.9652	0.4504	0.3678	0.5358
(A AND C) OR B HP	18	3	2	6	0.9250	0.8459	0.9652	0.4733	0.3898	0.5583
(A AND C) OR B HP	18	3	3	1	0.9375	0.8619	0.9730	0.3740	0.2959	0.4594
(A AND C) OR B HP	18	3	3	2	0.9375	0.8619	0.9730	0.4427	0.3605	0.5282
(A AND C) OR B HP	18	3	3	3	0.9250	0.8459	0.9652	0.4962	0.4119	0.5807
(A AND C) OR B HP	18	3	3	4	0.9250	0.8459	0.9652	0.5191	0.4342	0.6029
(A AND C) OR B HP	18	3	3	5	0.9250	0.8459	0.9652	0.5344	0.4492	0.6176
(A AND C) OR B HP	18	3	3	6	0.9250	0.8459	0.9652	0.5725	0.4869	0.6540
(A AND C) OR B HP	18	3	4	1	0.9250	0.8459	0.9652	0.4580	0.3751	0.5433
(A AND C) OR B HP	18	3	4	2	0.9250	0.8459	0.9652	0.5267	0.4417	0.6102
(A AND C) OR B HP	18	3	4	3	0.9125	0.8302	0.9570	0.5802	0.4945	0.6612
(A AND C) OR B HP	18	3	4	4	0.9125	0.8302	0.9570	0.6107	0.5252	0.6899
(A AND C) OR B HP	18	3	4	5	0.9000	0.8149	0.9485	0.6260	0.5406	0.7041
(A AND C) OR B HP	18	3	4	6	0.8875	0.7998	0.9397	0.6794	0.5953	0.7532
(A AND C) OR B HP	18	3	5	1	0.9125	0.8302	0.9570	0.4885	0.4045	0.5732
(A AND C) OR B HP	18	3	5	2	0.9125	0.8302	0.9570	0.5573	0.4718	0.6395
(A AND C) OR B HP	18	3	5	3	0.9000	0.8149	0.9485	0.6107	0.5252	0.6899
(A AND C) OR B HP	18	3	5	4	0.9000	0.8149	0.9485	0.6412	0.5561	0.7183
(A AND C) OR B HP	18	3	5	5	0.8875	0.7998	0.9397	0.6565	0.5718	0.7323
(A AND C) OR B HP	18	3	5	6	0.8750	0.7850	0.9307	0.7099	0.6271	0.7808
(A AND C) OR B HP	18	3	6	1	0.9000	0.8149	0.9485	0.5115	0.4268	0.5955
(A AND C) OR B HP	18	3	6	2	0.9000	0.8149	0.9485	0.5802	0.4945	0.6612
(A AND C) OR B HP	18	3	6	3	0.8875	0.7998	0.9397	0.6336	0.5484	0.7112
(A AND C) OR B HP	18	3	6	4	0.8875	0.7998	0.9397	0.6641	0.5796	0.7393
(A AND C) OR B HP	18	3	6	5	0.8750	0.7850	0.9307	0.6870	0.6032	0.7601
(A AND C) OR B HP	18	3	6	6	0.8500	0.7559	0.9121	0.7405	0.6593	0.8079
(A AND C) OR B HP	18	4	1	1	0.9500	0.8784	0.9804	0.2824	0.2124	0.3649
(A AND C) OR B HP	18	4	1	2	0.9500	0.8784	0.9804	0.3053	0.2330	0.3888
(A AND C) OR B HP	18	4	1	3	0.9500	0.8784	0.9804	0.3359	0.2607	0.4204
(A AND C) OR B HP	18	4	1	4	0.9500	0.8784	0.9804	0.3511	0.2747	0.4361
(A AND C) OR B HP	18	4	1	5	0.9500	0.8784	0.9804	0.3588	0.2817	0.4439
(A AND C) OR B HP	18	4	1	6	0.9500	0.8784	0.9804	0.3740	0.2959	0.4594
(A AND C) OR B HP	18	4	2	1	0.9250	0.8459	0.9652	0.3740	0.2959	0.4594
(A AND C) OR B HP	18	4	2	2	0.9250	0.8459	0.9652	0.4122	0.3316	0.4978
(A AND C) OR B HP	18	4	2	3	0.9250	0.8459	0.9652	0.4427	0.3605	0.5282
(A AND C) OR B HP	18	4	2	4	0.9250	0.8459	0.9652	0.4580	0.3751	0.5433
(A AND C) OR B HP	18	4	2	5	0.9250	0.8459	0.9652	0.4656	0.3824	0.5508
(A AND C) OR B HP	18	4	2	6	0.9250	0.8459	0.9652	0.4885	0.4045	0.5732
(A AND C) OR B HP	18	4	3	1	0.9250	0.8459	0.9652	0.4504	0.3678	0.5358
(A AND C) OR B HP	18	4	3	2	0.9250	0.8459	0.9652	0.4962	0.4119	0.5807
(A AND C) OR B HP	18	4	3	3	0.9250	0.8459	0.9652	0.5344	0.4492	0.6176
(A AND C) OR B HP	18	4	3	4	0.9250	0.8459	0.9652	0.5496	0.4642	0.6322
(A AND C) OR B HP	18	4	3	5	0.9250	0.8459	0.9652	0.5649	0.4793	0.6467
(A AND C) OR B HP	18	4	3	6	0.9250	0.8459	0.9652	0.6031	0.5175	0.6828
(A AND C) OR B HP	18	4	4	1	0.8875	0.7998	0.9397	0.5649	0.4793	0.6467
(A AND C) OR B HP	18	4	4	2	0.8875	0.7998	0.9397	0.6107	0.5252	0.6899
(A AND C) OR B HP	18	4	4	3	0.8875	0.7998	0.9397	0.6489	0.5639	0.7253
(A AND C) OR B HP	18	4	4	4	0.8875	0.7998	0.9397	0.6641	0.5796	0.7393
(A AND C) OR B HP	18	4	4	5	0.8875	0.7998	0.9397	0.6794	0.5953	0.7532
(A AND C) OR B HP	18	4	4	6	0.8875	0.7998	0.9397	0.7252	0.6432	0.7944
(A AND C) OR B HP	18	4	5	1	0.8750	0.7850	0.9307	0.5954	0.5098	0.6756
(A AND C) OR B HP	18	4	5	2	0.8750	0.7850	0.9307	0.6412	0.5561	0.7183

		Threshold				95% CI			95% CI	
Combination	EK	A	B	C	Sensitivity	Lower	Upper	Specificity	Lower	Upper
(A AND C) OR B HP	18	4	5	3	0.8750	0.7850	0.9307	0.6794	0.5953	0.7532
(A AND C) OR B HP	18	4	5	4	0.8750	0.7850	0.9307	0.6947	0.6112	0.7670
(A AND C) OR B HP	18	4	5	5	0.8750	0.7850	0.9307	0.7099	0.6271	0.7808
(A AND C) OR B HP	18	4	5	6	0.8750	0.7850	0.9307	0.7557	0.6756	0.8213
(A AND C) OR B HP	18	4	6	1	0.8625	0.7703	0.9215	0.6183	0.5329	0.6970
(A AND C) OR B HP	18	4	6	2	0.8625	0.7703	0.9215	0.6641	0.5796	0.7393
(A AND C) OR B HP	18	4	6	3	0.8625	0.7703	0.9215	0.7023	0.6191	0.7739
(A AND C) OR B HP	18	4	6	4	0.8625	0.7703	0.9215	0.7176	0.6351	0.7876
(A AND C) OR B HP	18	4	6	5	0.8625	0.7703	0.9215	0.7405	0.6593	0.8079
(A AND C) OR B HP	18	4	6	6	0.8500	0.7559	0.9121	0.7863	0.7084	0.8478
(A AND C) OR B HP	18	5	1	1	0.9500	0.8784	0.9804	0.2977	0.2261	0.3809
(A AND C) OR B HP	18	5	1	2	0.9500	0.8784	0.9804	0.3206	0.2468	0.4047
(A AND C) OR B HP	18	5	1	3	0.9500	0.8784	0.9804	0.3511	0.2747	0.4361
(A AND C) OR B HP	18	5	1	4	0.9500	0.8784	0.9804	0.3664	0.2888	0.4516
(A AND C) OR B HP	18	5	1	5	0.9500	0.8784	0.9804	0.3740	0.2959	0.4594
(A AND C) OR B HP	18	5	1	6	0.9500	0.8784	0.9804	0.3893	0.3101	0.4748
(A AND C) OR B HP	18	5	2	1	0.9250	0.8459	0.9652	0.4046	0.3244	0.4902
(A AND C) OR B HP	18	5	2	2	0.9250	0.8459	0.9652	0.4427	0.3605	0.5282
(A AND C) OR B HP	18	5	2	3	0.9250	0.8459	0.9652	0.4733	0.3898	0.5583
(A AND C) OR B HP	18	5	2	4	0.9250	0.8459	0.9652	0.4885	0.4045	0.5732
(A AND C) OR B HP	18	5	2	5	0.9250	0.8459	0.9652	0.4962	0.4119	0.5807
(A AND C) OR B HP	18	5	2	6	0.9250	0.8459	0.9652	0.5115	0.4268	0.5955
(A AND C) OR B HP	18	5	3	1	0.9250	0.8459	0.9652	0.5038	0.4193	0.5881
(A AND C) OR B HP	18	5	3	2	0.9250	0.8459	0.9652	0.5496	0.4642	0.6322
(A AND C) OR B HP	18	5	3	3	0.9250	0.8459	0.9652	0.5878	0.5022	0.6684
(A AND C) OR B HP	18	5	3	4	0.9250	0.8459	0.9652	0.6031	0.5175	0.6828
(A AND C) OR B HP	18	5	3	5	0.9250	0.8459	0.9652	0.6183	0.5329	0.6970
(A AND C) OR B HP	18	5	3	6	0.9250	0.8459	0.9652	0.6412	0.5561	0.7183
(A AND C) OR B HP	18	5	4	1	0.8750	0.7850	0.9307	0.6260	0.5406	0.7041
(A AND C) OR B HP	18	5	4	2	0.8750	0.7850	0.9307	0.6718	0.5875	0.7463
(A AND C) OR B HP	18	5	4	3	0.8750	0.7850	0.9307	0.7099	0.6271	0.7808
(A AND C) OR B HP	18	5	4	4	0.8750	0.7850	0.9307	0.7252	0.6432	0.7944
(A AND C) OR B HP	18	5	4	5	0.8750	0.7850	0.9307	0.7405	0.6593	0.8079
(A AND C) OR B HP	18	5	4	6	0.8750	0.7850	0.9307	0.7634	0.6837	0.8280
(A AND C) OR B HP	18	5	5	1	0.8625	0.7703	0.9215	0.6565	0.5718	0.7323
(A AND C) OR B HP	18	5	5	2	0.8625	0.7703	0.9215	0.7023	0.6191	0.7739
(A AND C) OR B HP	18	5	5	3	0.8625	0.7703	0.9215	0.7405	0.6593	0.8079
(A AND C) OR B HP	18	5	5	4	0.8625	0.7703	0.9215	0.7557	0.6756	0.8213
(A AND C) OR B HP	18	5	5	5	0.8625	0.7703	0.9215	0.7710	0.6919	0.8346
(A AND C) OR B HP	18	5	5	6	0.8625	0.7703	0.9215	0.7939	0.7167	0.8543
(A AND C) OR B HP	18	5	6	1	0.8375	0.7416	0.9025	0.6794	0.5953	0.7532
(A AND C) OR B HP	18	5	6	2	0.8375	0.7416	0.9025	0.7252	0.6432	0.7944
(A AND C) OR B HP	18	5	6	3	0.8375	0.7416	0.9025	0.7634	0.6837	0.8280
(A AND C) OR B HP	18	5	6	4	0.8375	0.7416	0.9025	0.7786	0.7002	0.8412
(A AND C) OR B HP	18	5	6	5	0.8375	0.7416	0.9025	0.8015	0.7251	0.8608
(A AND C) OR B HP	18	5	6	6	0.8375	0.7416	0.9025	0.8244	0.7503	0.8801
(A AND C) OR B HP	18	6	1	1	0.9500	0.8784	0.9804	0.3282	0.2537	0.4125
(A AND C) OR B HP	18	6	1	2	0.9500	0.8784	0.9804	0.3511	0.2747	0.4361
(A AND C) OR B HP	18	6	1	3	0.9500	0.8784	0.9804	0.3740	0.2959	0.4594
(A AND C) OR B HP	18	6	1	4	0.9500	0.8784	0.9804	0.3740	0.2959	0.4594
(A AND C) OR B HP	18	6	1	5	0.9500	0.8784	0.9804	0.3817	0.3030	0.4671
(A AND C) OR B HP	18	6	1	6	0.9500	0.8784	0.9804	0.3969	0.3172	0.4825
(A AND C) OR B HP	18	6	2	1	0.9250	0.8459	0.9652	0.4351	0.3533	0.5207

		Threshold				95% CI			95% CI	
Combination	EK	A	B	C	Sensitivity	Lower	Upper	Specificity	Lower	Upper
(A AND C) OR B HP	18	6	2	2	0.9250	0.8459	0.9652	0.4733	0.3898	0.5583
(A AND C) OR B HP	18	6	2	3	0.9250	0.8459	0.9652	0.4962	0.4119	0.5807
(A AND C) OR B HP	18	6	2	4	0.9250	0.8459	0.9652	0.4962	0.4119	0.5807
(A AND C) OR B HP	18	6	2	5	0.9250	0.8459	0.9652	0.5038	0.4193	0.5881
(A AND C) OR B HP	18	6	2	6	0.9250	0.8459	0.9652	0.5191	0.4342	0.6029
(A AND C) OR B HP	18	6	3	1	0.9250	0.8459	0.9652	0.5496	0.4642	0.6322
(A AND C) OR B HP	18	6	3	2	0.9250	0.8459	0.9652	0.5954	0.5098	0.6756
(A AND C) OR B HP	18	6	3	3	0.9250	0.8459	0.9652	0.6260	0.5406	0.7041
(A AND C) OR B HP	18	6	3	4	0.9250	0.8459	0.9652	0.6260	0.5406	0.7041
(A AND C) OR B HP	18	6	3	5	0.9250	0.8459	0.9652	0.6336	0.5484	0.7112
(A AND C) OR B HP	18	6	3	6	0.9250	0.8459	0.9652	0.6565	0.5718	0.7323
(A AND C) OR B HP	18	6	4	1	0.8750	0.7850	0.9307	0.6718	0.5875	0.7463
(A AND C) OR B HP	18	6	4	2	0.8750	0.7850	0.9307	0.7176	0.6351	0.7876
(A AND C) OR B HP	18	6	4	3	0.8750	0.7850	0.9307	0.7481	0.6674	0.8146
(A AND C) OR B HP	18	6	4	4	0.8750	0.7850	0.9307	0.7481	0.6674	0.8146
(A AND C) OR B HP	18	6	4	5	0.8750	0.7850	0.9307	0.7557	0.6756	0.8213
(A AND C) OR B HP	18	6	4	6	0.8750	0.7850	0.9307	0.7786	0.7002	0.8412
(A AND C) OR B HP	18	6	5	1	0.8625	0.7703	0.9215	0.7023	0.6191	0.7739
(A AND C) OR B HP	18	6	5	2	0.8625	0.7703	0.9215	0.7481	0.6674	0.8146
(A AND C) OR B HP	18	6	5	3	0.8625	0.7703	0.9215	0.7786	0.7002	0.8412
(A AND C) OR B HP	18	6	5	4	0.8625	0.7703	0.9215	0.7786	0.7002	0.8412
(A AND C) OR B HP	18	6	5	5	0.8625	0.7703	0.9215	0.7863	0.7084	0.8478
(A AND C) OR B HP	18	6	5	6	0.8625	0.7703	0.9215	0.8092	0.7334	0.8673
(A AND C) OR B HP	18	6	6	1	0.8375	0.7416	0.9025	0.7328	0.6512	0.8012
(A AND C) OR B HP	18	6	6	2	0.8375	0.7416	0.9025	0.7786	0.7002	0.8412
(A AND C) OR B HP	18	6	6	3	0.8375	0.7416	0.9025	0.8092	0.7334	0.8673
(A AND C) OR B HP	18	6	6	4	0.8375	0.7416	0.9025	0.8092	0.7334	0.8673
(A AND C) OR B HP	18	6	6	5	0.8375	0.7416	0.9025	0.8168	0.7419	0.8737
(A AND C) OR B HP	18	6	6	6	0.8375	0.7416	0.9025	0.8397	0.7673	0.8927

Note. EK = Evaluation key; CI = Confidence interval; HP = Headphones.

The file `HALT_all_tests_and_combinations_CHARACTERISTICS.txt` at https://osf.io/43gva/ contains a comma-seperated version of this table. In addition, the table is available as a data frame in the HALT R-package.

Figure S1

Sequence Plan of the Pre-Study

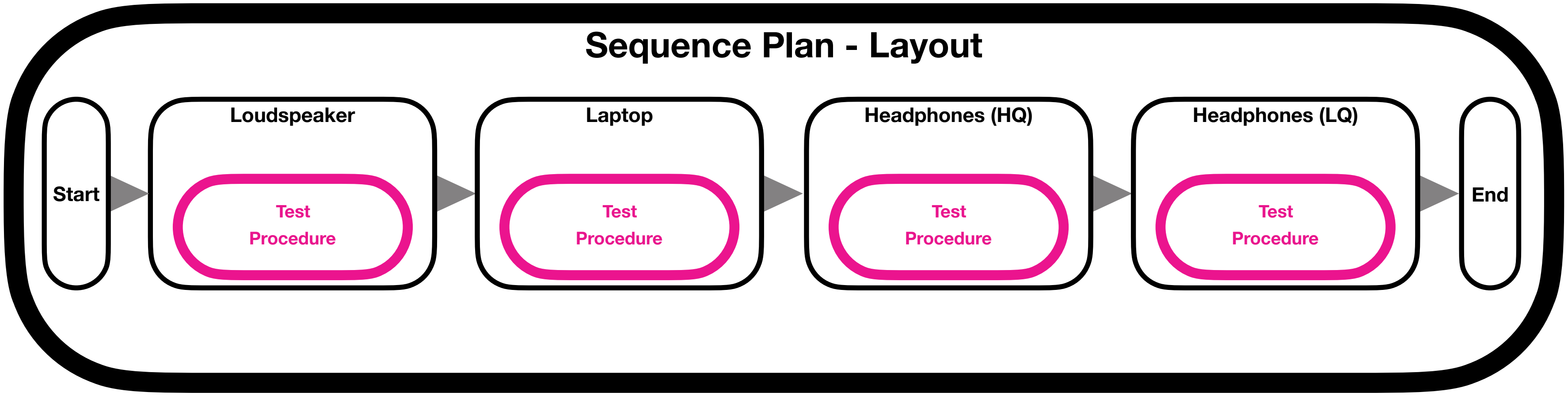

Figure S2

Flow Chart – Participant Exclusion in the Main Study

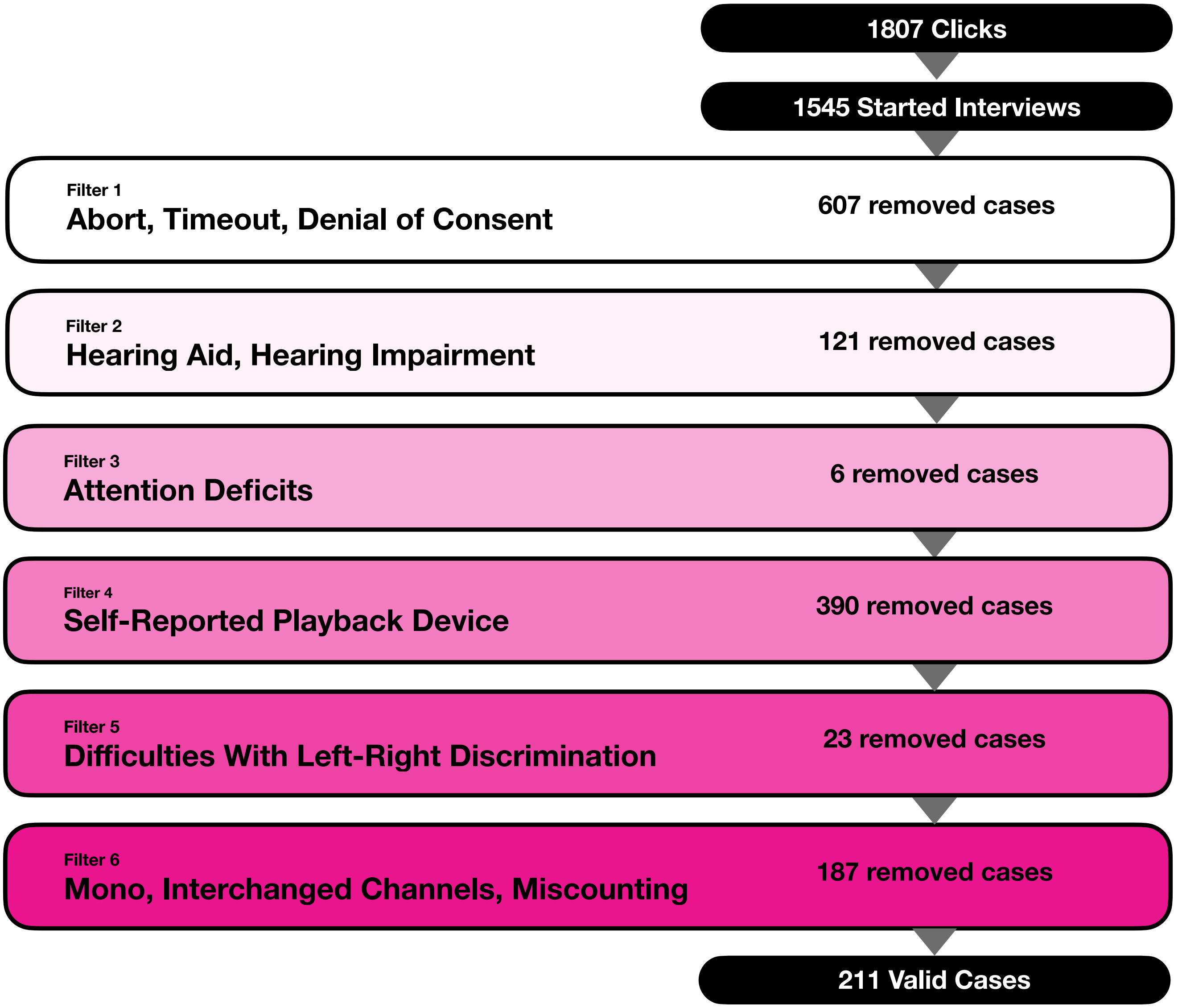

Figure S3

Sequence Plan for the Complete HALT Procedure

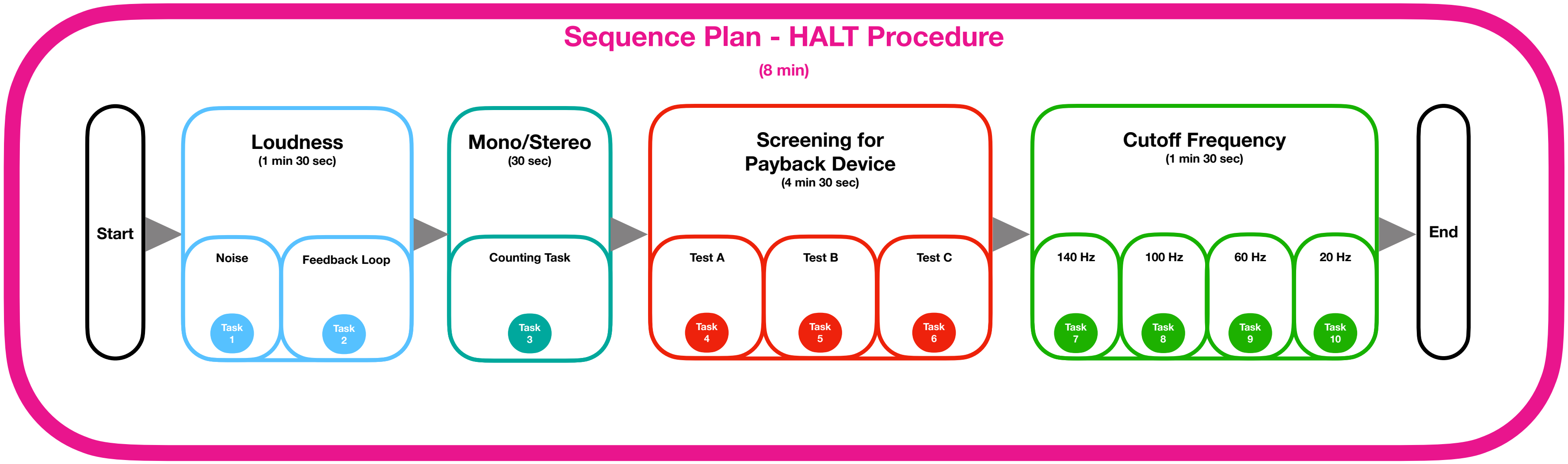

Figure S4

Tree Diagram for SCC with Headphones as Target Device

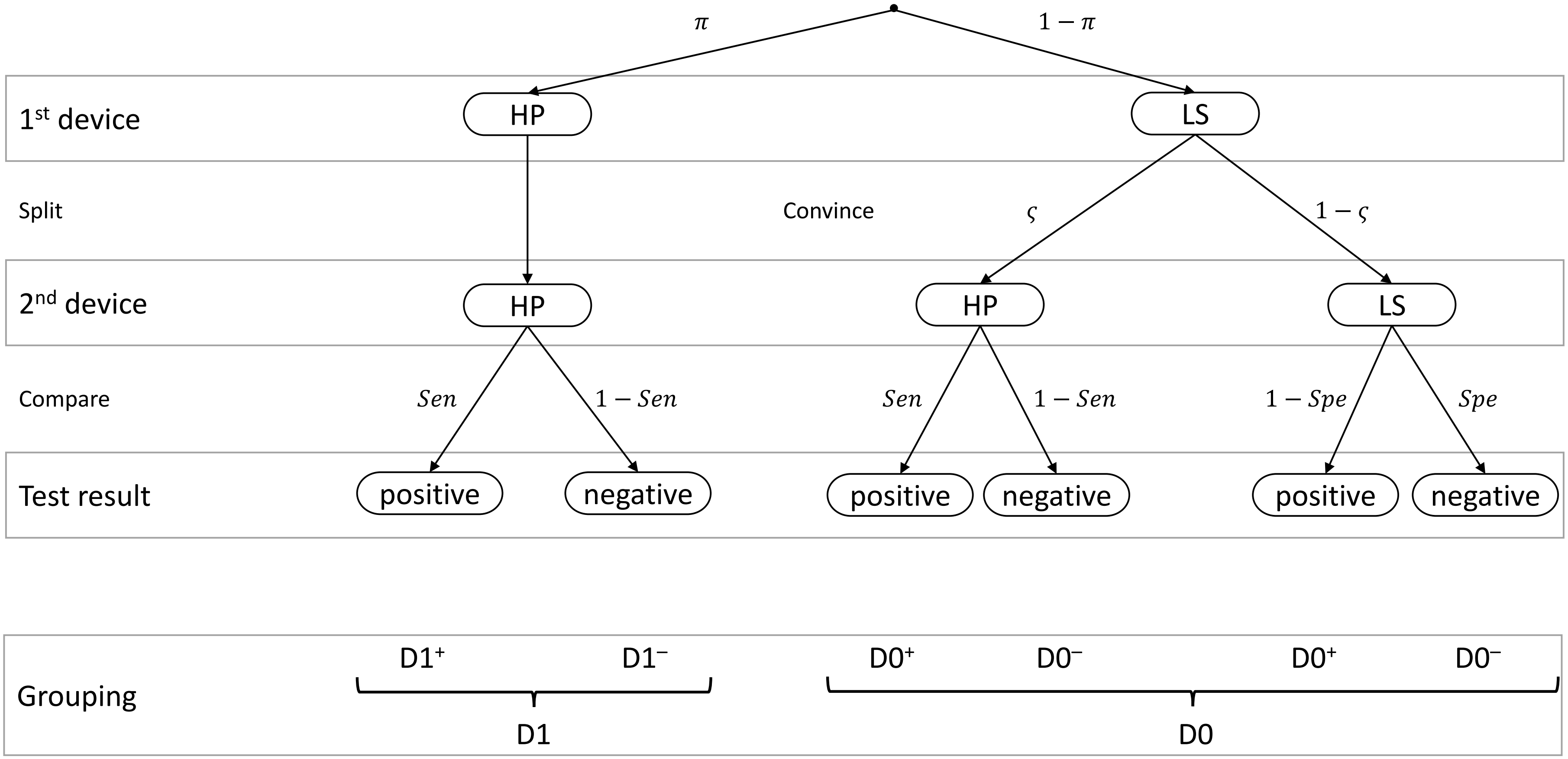

Note. π denotes the unbiased prevalence of headphones in the target population. ς denotes the switching prevalence in the target population, i.e. the probability that a participant switches to headphones after being prompted to do so. Sen = Sensitivity; Spe = Specificity.

Section S1 Normal Approximation of the Binomial Distribution of True Headphone Users for a priori Considerations for FWR and FAR

Let H denote the number of true headphone users in a sample of n persons with a positive result from a screening procedure with known sensitivity and specificity. When the population from which the sample is drawn is not too small we can conceptualize H as a random variable following a Binomial distribution with size n and a probability of success, i.e. a true headphone user, equaling the PPV for a prevalence estimate $\hat{\pi}$ and the screening test used. For $k \in \mathbb{N}$, the probability that the true number of headphone users equals k is given by

$$\mathbb{P}(H = k) = \binom{n}{k} PPV^k (1 - PPV)^{n-k}$$

(see for example Jacod & Protter, 2004, pp. 23, 24, 30). Then the probability that k or more persons were using headphones is

$$\mathbb{P}(H \geq k) = \sum_{i=k}^{n} \binom{n}{i} PPV^k (1 - PPV)^{n-i}.$$

The expectation of this Binomial distribution is $n \cdot PPV$ and its variance is given by $n \cdot PPV \cdot (1 - PPV)$ (see for example Jacod & Protter, 2004, pp. 30, 119).

The De Moivre–Laplace theorem states for $n, k \in \mathbb{N}, k \leq n, 0 < p < 1$ and a random variable Y following a Binomial distribution of size n and probability of success p that

$$\lim_{n \to \infty} \left(\mathbb{P}(Y \leq k) - \phi\left(\frac{k - np}{\sqrt{np(1-p)}} \right) \right) = 0$$

when ϕ denotes the cumulative distribution function of the standard normal distribution (see for example Georgii, 2004, pp 129–135). For $k \leq l \leq n$ we get with a continuity correction that

$$\mathbb{P}(k \leq Y \leq l) \approx \phi\left(\frac{l + \frac{1}{2} - np}{\sqrt{np(1-p)}} \right) - \phi\left(\frac{k - \frac{1}{2} - np}{\sqrt{np(1-p)}} \right).$$

Equivalently, we could say that Y is approximated by S when S is a Normal distribution with expectation np and variance $np(1-p)$.

Let $\vartheta := \mathbb{P}(H \geq k)$. Since $\mathbb{P}(H \geq k) = 1 - \mathbb{P}(H < k)$ the following holds approximately for $p = PPV$

$$\begin{aligned}
\mathbb{P}(H < k) = 1 - \vartheta &= \phi\left(\frac{k - \frac{1}{2} - np}{\sqrt{np(1-p)}} \right) \\
\phi^{-1}(1 - \vartheta) &= \frac{k - \frac{1}{2} - np}{\sqrt{np(1-p)}} \\
\sqrt{np(1-p)} \cdot \phi^{-1}(1 - \vartheta) &= k - \frac{1}{2} - np \\
np(1-p) \cdot \left(\phi^{-1}(1 - \vartheta)\right)^2 &= \left(k - \frac{1}{2}\right)^2 - 2\left(k - \frac{1}{2}\right) np + n^2 p^2
\end{aligned}$$

$$\begin{aligned}
\Rightarrow 0 &= \left(k - \frac{1}{2}\right)^2 - 2\left(k - \frac{1}{2}\right) np + n^2p^2 - np(1-p) \cdot \left(\phi^{-1}(1 - \vartheta)\right)^2 \\
0 &= n^2p^2 - n\left((2k-1)p + p(1-p)\left(\phi^{-1}(1-\vartheta)\right)\right) + \left(k - \frac{1}{2}\right) \\
0 &= n^2 - n\frac{1}{p^2}\left((2k-1)p + p(1-p)\left(\phi^{-1}(1-\vartheta)\right)\right) + \frac{1}{p^2}\left(k - \frac{1}{2}\right) \\
0 &= n^2 + n\underbrace{\left(-\frac{1}{p}\left(2k - 1 + (1-p)\left(\phi^{-1}(1-\vartheta)\right)^2\right)\right)}_{=:a} + \underbrace{\left(\frac{k - \frac{1}{2}}{p}\right)^2}_{=:b}
\end{aligned}$$

$$\Rightarrow n_{1,2} = -\frac{a}{2} \pm \sqrt{\left(\frac{a}{2}\right)^2 - b}$$

For a given $\vartheta \in \left]\frac{1}{2}, 1\right[$ and a given $k \in \mathbb{N}$ the probability that at least k out of n persons were using headphones is approximately ϑ, i. e. $\mathbb{P}\left(H \geq k\right)$, when

$$n = n_1 = -\frac{a}{2} + \sqrt{\left(\frac{a}{2}\right)^2 - b}$$

Section S2 Probabilities and a priori sample size-based estimation for the Split–Convince–Compare (SCC) Strategy

The final sample for SCC consists of the groups D1 and D0$^+$. D1 consist of participants who indicated use of headphones. Participants who indicated use of a device other than the target device and got a positive test result are in D0$^+$. Let H denote the number of true headphone users in the final sample and n be the final sample size, i.e. the number of participants in D1 and D0$^+$. We conceptualize H as random variable following a Binomial distribution with size n and probability of success $\tilde{p}$. $\tilde{p}$ is the probability that a participant was using headphones given that he is in D1 or D0$^+$. As can be seen in Figure S4, a participant is in group D1 with a probability of π and in group D0$^+$ with a probability of $(1-\pi)\left(\varsigma \cdot Sen + (1-\varsigma)(1-Spe)\right)$. The probability for a participant using headphones and being in group D0$^+$ is $(1-\pi)\cdot\varsigma\cdot Sen$. Therefore, the probability that a participant was using headphones given that he is in D1 or D0$^+$ is

$$\begin{aligned}\tilde{p} \coloneqq \mathbb{P}\left(\text{headphones} \,\middle|\, \text{D1 or D0}^+\right) &= \frac{\pi + (1-\pi)\cdot\varsigma\cdot Sen}{\pi + (1-\pi)\cdot\varsigma\cdot Sen + (1-\pi)(1-\varsigma)(1-Spe)} \\ &= \frac{\pi + (1-\pi)\cdot\varsigma\cdot Sen}{\pi + (1-\pi)\left(\varsigma\cdot Sen + (1-\varsigma)(1-Spe)\right)}\end{aligned}$$

For $k \in \mathbb{N}$, the probability that the true number of headphone users equals k is given by

$$\mathbb{P}\left(H = k\right) = \binom{n}{k}\tilde{p}^k(1-\tilde{p})^{n-k}$$

(see for example Jacod & Protter, 2004, pp. 23, 24, 30) and the probability that k or more persons were using headphones, which is denoted by ϑ is

$$\mathbb{P}\left(H \geq k\right) = \sum_{i=k}^{n}\binom{n}{i}\tilde{p}^i(1-\tilde{p})^{n-i}.$$

After a Normal approximation (for details, see Section S1), the number of participants in D1 and D0$^+$, the sample size n, can be calculated for a given $\vartheta \in \left]\frac{1}{2}, 1\right[$ and a given $k \in \mathbb{N}$:

$$n = -\frac{a}{2} + \sqrt{\left(\frac{a}{2}\right)^2 - b}$$

with $a = -\frac{1}{\tilde{p}}\left(2k - 1 + (1-\tilde{p})\left(\phi^{-1}\left(1-\vartheta\right)\right)^2\right)$ and $b = \left(\frac{k-\frac{1}{2}}{\tilde{p}}\right)^2$.
